# Vertical support use and primate origins

**DOI:** 10.1038/s41598-019-48651-x

**Published:** 2019-08-26

**Authors:** Gabriel S. Yapuncich, Henry J. Feng, Rachel H. Dunn, Erik R. Seiffert, Doug M. Boyer

**Affiliations:** 1 Department of Evolutionary Anthropology, Duke University, Durham, NC 27708 USA; 2 Department of Anatomy, Des Moines University, Des Moines, IA 50312 USA; 3 Department of Integrative Anatomical Sciences, Keck School of Medicine, University of Southern California, Los Angeles, CA 90033 USA

**Keywords:** Biological anthropology, Palaeontology, Biomechanics

## Abstract

Adaptive scenarios of crown primate origins remain contentious due to uncertain order of acquisition and functional significance of the clade’s diagnostic traits. A feature of the talus bone in the ankle, known as the posterior trochlear shelf (PTS), is well-regarded as a derived crown primate trait, but its adaptive significance has been obscured by poorly understood function. Here we propose a novel biomechanical function for the PTS and model the talus as a cam mechanism. By surveying a large sample of primates and their closest relatives, we demonstrate that the PTS is most strongly developed in extant taxa that habitually grasp vertical supports with strongly dorsiflexed feet. Tali of the earliest fossils likely to represent crown primates exhibit more strongly developed PTS cam mechanisms than extant primates. As a cam, the PTS may increase grasping efficiency in dorsiflexed foot postures by increasing the path length of the flexor fibularis tendon, and thus improve the muscle’s ability to maintain flexed digits without increasing energetic demands. Comparisons are made to other passive digital flexion mechanisms suggested to exist in other vertebrates. These results provide robust anatomical evidence that the habitual vertical support use exerted a strong selective pressure during crown primate origins.

## Introduction

**Figure 1:**
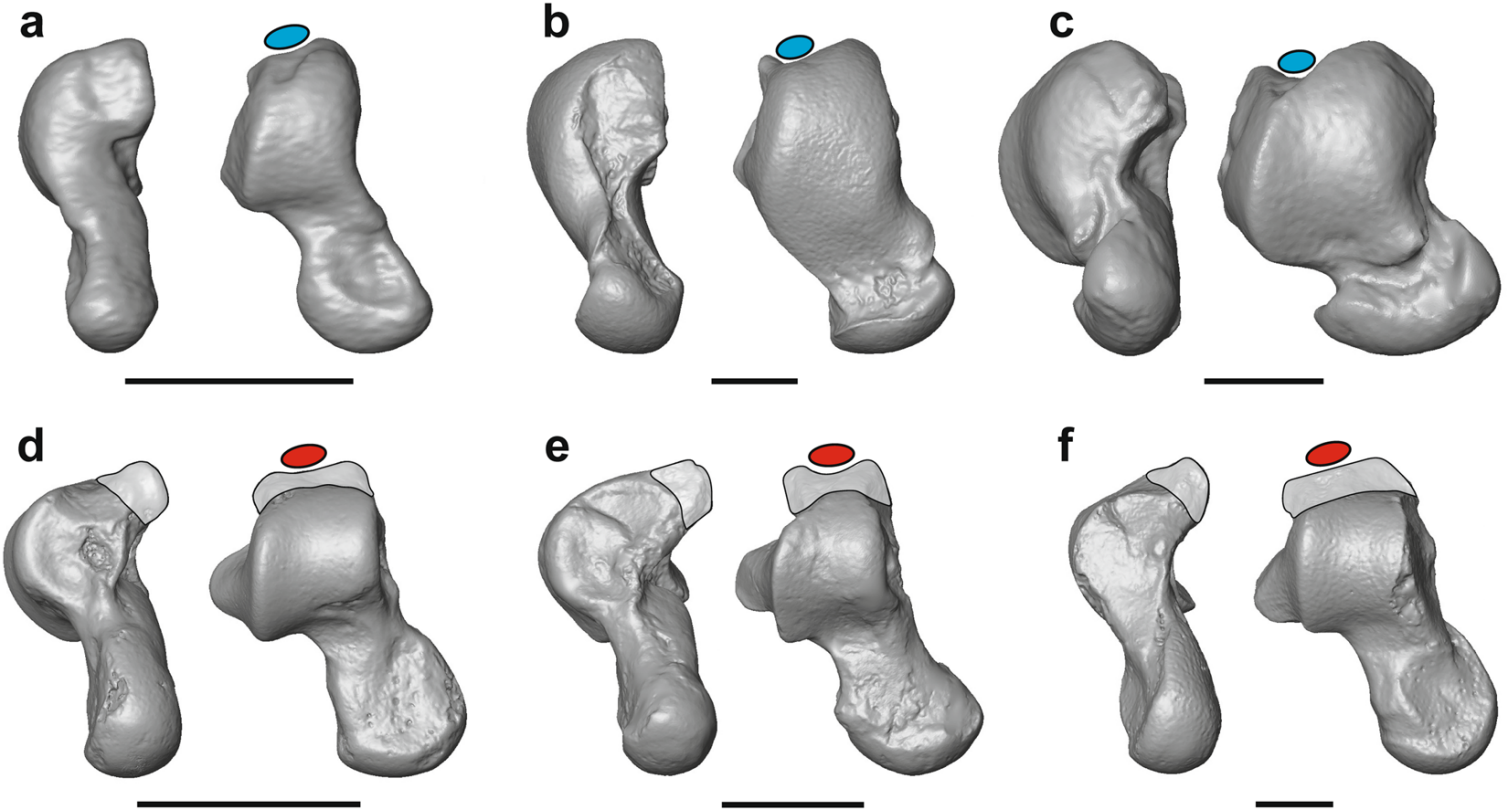
Presence and absence of the posterior trochlear shelf (PTS) in euarchontans. PTS indicated by shading. Ellipses represent flexor fibularis tendon; tendons in red are expected to experience cam effect, while tendons in blue are not. Non-crown primates include (**a**) *Ptilocercus lowii* (USNM 488072), (**b**) *Galeopterus variegatus* (USNM 317118), and (**c**) ^†^*Plesiadapis rex* (UM 94816; reversed for consistency). Extant and likely crown primates include (**d**) ^†^*Teilhardina belgica* (IRSNB M1235), (**e**) ^†^*Donrussellia provincialis* (MNHN RI 428), and (**f**) *Lepilemur mustelinus* (AMNH 170556). Institution abbreviations and expanded comparative plates are provided in Supplementary Material. Scale bars equal 3 mm.

The talus is an important element for reconstructing positional behavior throughout primate evolution because the bone’s morphology correlates well with locomotor and postural behaviors of living euarchontans (the mammalian clade including Primates, Scandentia, Dermoptera) and it is frequently preserved in fossil assemblages^1–4^. However, despite a long history of study^1,4–7^, debate remains concerning the talar morphology of the common ancestor of crown primates and the positional behavior implied by this morphology. The posterior trochlear shelf (PTS) – a bony extension protruding from the posterior aspect of the talar body (Fig. 1) – is a prime example of a conspicuous but confounding talar feature: while PTS hypertrophy unequivocally diagnoses a crown primate talus^1^, there is no consensus regarding the feature’s functional significance^2,7^. Several biomechanical roles have been suggested, including creating a bony stop during plantarflexion^1,2^, supporting an elongated posterior calcaneal facet^1,4^, or redirecting stress within the talus^4^. The ambiguous function of the PTS means it is often characterized but rarely emphasized in the description of fossil tali^3,7^. Resolving this gap in understanding of primate functional anatomy can provide novel insights into the origin of our order.

**Figure 2:**
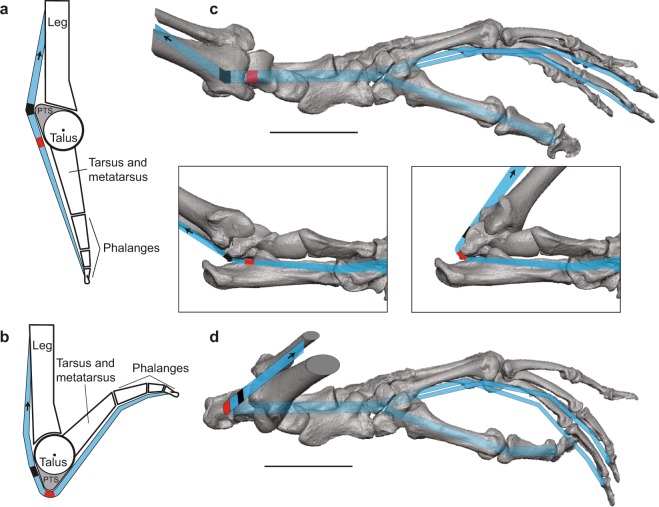
The posterior trochlear shelf (PTS) as a cam mechanism. Line drawings of cam mechanism of the PTS in a plantarflexed (**a**) and dorsiflexed (**b**) foot. Cam mechanism modeled in plantarflexed (**c**) and dorsiflexed (**d**) foot of *Mirza zaza* (DLC 315 m), dorsal view. Insets show plantarflexed and dorsiflexed foot in medioplantar view. Blue lines indicate paths of the tendons of flexor fibularis, with black and red marks indicating theoretical contact points between PTS and tendon during plantarflexion and dorsiflexion respectively. Insertion patterns follow Langdon^12^. Arrow indicated by F shows direction of force generated by flexor fibularis.

From a biomechanical perspective, PTS hypertrophy accentuates an asymmetry around the axis of the talocrural joint. As a hinge joint, the talocrural joint is primarily involved in dorsi- and plantarflexion of the foot as the talus rotates sagittally around a largely transverse axis^8^ (although more complex movements in other anatomical planes occur at the joint as well^9,10^). A rotating, asymmetrical body can function as a cam mechanism (Fig. S1a), converting rotational into translational motion^11^. Cams consist of two moving elements: a driver, which is generally fixed at a rotational axis and has an asymmetry known as the rise, and a follower, which contacts the driver and follows a path dictated by the contour of the rise (Fig. S1a). We hypothesize that the talus functions as a driver rotating about the talocrural joint axis, the PTS serves as the rise, and the tendon of the flexor fibularis muscle (transmitted through a groove on the posterior aspect of the talus) as the follower. Rotation of the driver (dorsi- and plantarflexion of the foot) would “translate” the tendon proximally by increasing its path length, and a larger rise (i.e., PTS hypertrophy) would increase the effect. Since the PTS is located on the posterior aspect of the talus, the tendon’s path length would be maximized when the PTS is most distal to the origin of the flexor fibularis (i.e., when the foot is dorsiflexed) and the tendon would be “translated” toward the muscle’s origin, effectively contracting the muscle (Fig. 2).

In non-human primates, the flexor fibularis typically inserts on the distal phalanges of the first, third, and fourth rays^12^, so the PTS cam mechanism could confer several biomechanical benefits in taxa with grasping feet. Given the muscle’s insertion patterns and assuming muscular contraction remains constant, translation of the tendon may induce additional flexion of digits critical for pedal grasping (Fig. 2). Alternatively, increasing the path length of the tendon during dorsiflexion could permit reduced muscular activity without a concomitant decrease in grasping ability. Finally, the PTS cam could increase the passive tension within the muscle: when stretched to a certain length, the actin-myosin interaction of sarcomeres is maximized, permitting increased force generation^13^. If the flexor fibularis can stretch the additional distance necessitated by a hypertrophied PTS, the cam mechanism could shift the muscle to a more efficient position along its length-tension curve. In the discussion, we examine the likelihood of each of these alternatives, but in all cases, a hypertrophied PTS would increase grasping efficiency (increased work for equivalent energy or equivalent work for reduced energy) in a dorsiflexed foot posture.

To model the PTS as a cam mechanism, our measurements capture the size of the rise relative to the base circle of the driver, a ratio termed the PTS index (Fig. S1b). PTS indices > 1 indicate strongly developed cam mechanisms (Materials and Methods). We compute PTS indices for a large and comprehensive sample of euarchontans (Materials and Methods) and test for significant differences between clades as well as significant differences from a PTS index = 1 (a null hypothesis indicating the rise is equivalent to the driver’s base circle). Finally, we evaluate the evolution of the PTS through time using Bayesian ancestral state reconstruction and multiple-regime Ornstein-Uhlenbeck models (Materials and Methods).

## Results

**Figure 3:**
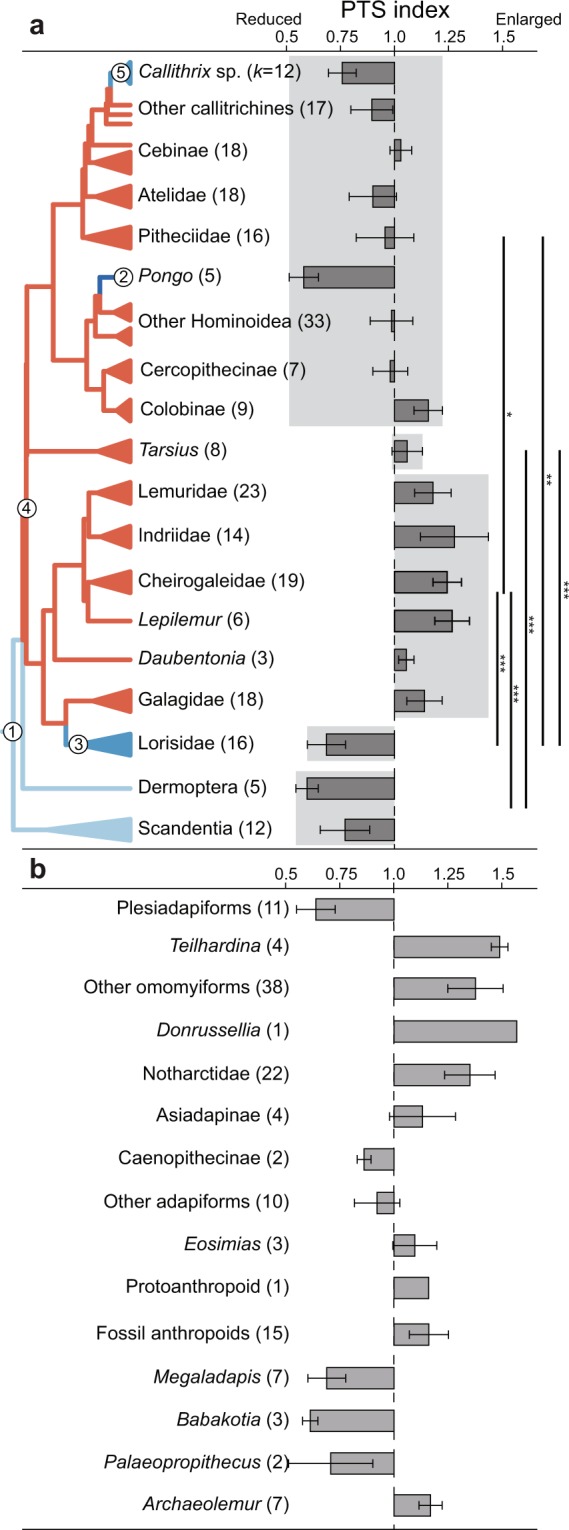
Bar charts of PTS indices, clade-level ANOVA results, and adaptive regime shifts in extant (**a**) and extinct (**b**) euarchontans. Whiskers indicate standard deviation. Individual sample sizes indicated in parentheses. Pairwise ANOVAs (F = 27.65, df = 4,68) were computed between groups shaded in light gray using species means and are reported in full in Table S7. Asterisks denote p-values ***p < 0.001, **p < 0.01, *p < 0.05. SURFACE regimes are mapped onto extant phylogeny. Nodes with regime shifts are numbered and detailed in Table S21. Branch colors indicate regime shifts (red: Ɵ > 1.0, blue Ɵ < 1.0); color intensity indicates rank order of Ɵ values (darker colors indicate more extreme optima). Boxplots of PTS indices for all species are shown in Fig. S6.

Among extant primates, the highest PTS indices are exhibited by indriids and *Lepilemur* (Fig. 3a; Table S1; Fig. S6). Four extant primate families (Indriidae, Lemuridae, Galagidae, and Tarsiidae), as well as a non-natural group of the remaining lemuriform taxa (Cheirogaleidae, *Lepilemur*, and *Daubentonia*) have mean PTS indices significantly greater than 1 (Table S8). Of these taxa, indriids, galagids, and tarsiers habitually grasp vertically oriented supports with strongly dorsiflexed feet^14–16^, and vertical support use is a component of the positional behavior for many lemuriforms^17^. As a group, strepsirrhines (excluding Lorisidae) have significantly greater PTS indices than anthropoids, lorisids, and other euarchontans (dermopterans and scandentians) (Fig. 3a; Table S7; Fig. S5).

**Figure 4:**
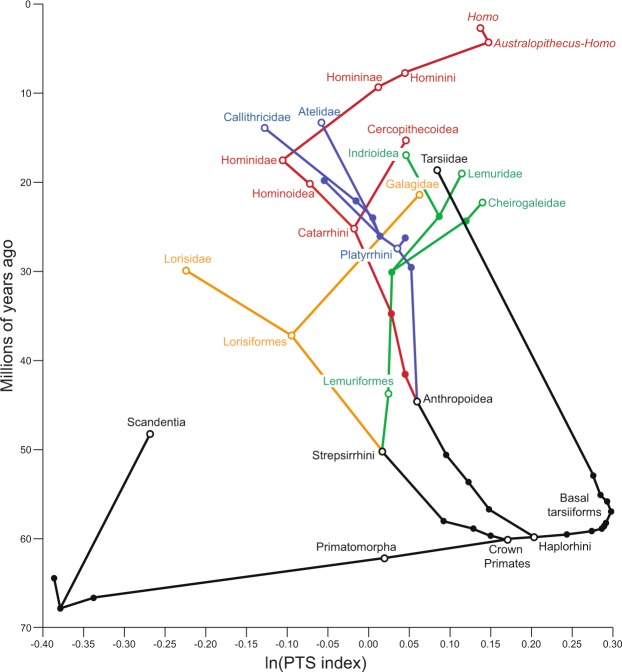
Ancestral state reconstruction (delta model with random walk) of PTS index for select nodes of the euarchontan tree. Branches are colored by clade to improve readability, internal nodes of interest (open circles) are labeled. Mean estimates and confidence intervals for each node are presented in Table S13. Additional discussion of PTS evolution within hominins is presented in Supplementary Material.

The high PTS indices of extant vertical clinging taxa are exceeded by those of several fossil groups. Members of both adapiforms and omomyiforms, two Eocene groups likely to represent crown primates, including the earliest represented members (*Donrussellia* and *Teilhardina* respectively) (Fig. 3b; Table S2) have greater PTS indices than those observed in extant taxa. The best supported model for Bayesian ancestral state reconstruction (Fig. 4, Table S13) utilizes a delta parameter <1 (mean δ = 0.544) and estimates that the ancestral crown primate possessed a well-developed PTS (1.19), in stark contrast to all other euarchontan groups, including plesiadapiforms (0.68).

Evolutionary models generated with stepwise-fitting of Ornstein-Uhlenbeck adaptive regimes accord well with the evolutionary pattern implied by ancestral state reconstruction. For extant taxa (Fig. 3a; Table S21), the base of the euarchontan tree is characterized by a low adaptive optimum for the PTS index (0.74) which is maintained in living dermopterans and scandentians. A shift to a higher optimum (1.59) occurs at the node representing the common ancestor of crown primates. Including fossils generates a best-fit model (Fig. S13; Table S17; Table S20) with a high optimum (1.11) at a more basal node (Primatomorpha = crown primates and dermopterans). While the crown primate node maintains the same high optimum as Primatomorpha, the dermopteran lineage immediately shifts to a lower optimum (0.61).

Several euarchontans exhibit mean PTS indices significantly <1 and thus experience no cam effect in dorsiflexed foot postures (Fig. 3; Table S8). These taxa fall into two functional groups: claw-clinging taxa (dermopterans, scandentians, callitrichines) and taxa that grasp substrates over large ankle excursion angles (lorisids, atelids and *Pongo pygmaeus*) (Fig. 3a; Fig. S6). Ornstein-Uhlenbeck modeling recovers regime shifts to lower optima for members of both groups (Fig. 3a; Fig. S13). Though members of the first group often use vertical supports^18–20^, claws fundamentally change how the animal engages with the substrate^21^, so maintaining vertical postures does not require a strong pedal grasp between opposing digits. Fossil taxa with claws also have low PTS indices, as vertical clinging plesiadapiforms^22^ have PTS indices <1 (Fig. 3b; Table S2). In lorisids, atelids, and *Pongo*, low PTS indices mean the muscle tendon passes closer to the talocrural joint axis, so muscle path length does not change substantially as foot position changes. Eliminating the PTS cam would have the benefit of reducing variability in the muscular effort experienced as a result of these taxa using a wide range of foot postures while grasping. Among examined fossil taxa, adapines, caenopithecines, and sloth and koala lemurs, which have been compared to slow-climbing or suspensory taxa^23–25^, exhibit PTS indices <1 (Fig. 3b).

In most extant anthropoid families, the talus does not exhibit a pronounced cam mechanism, and PTS indices are not significantly different from 1 (Fig. 3a; Table S8). Compared to strepsirrhines, anthropoids utilize more horizontal supports^26^ with less hallucal grasping^27^, so anthropoids may not experience the same selective pressures as strepsirrhines to maintain a hypertrophied PTS. Only two anthropoid groups have PTS indices notably different from 1. First, as discussed above, atelids and callitrichines have mean PTS indices significantly <1 (Fig. 3a; Table S8). Second, cercopithecoids, particularly the Asian colobines in our sample, exhibit high PTS values (Fig. 3a). Though data is limited, vertical support use appears to be an important component of positional behavior for some colobines^28^. Most fossil anthropoids have PTS indices slightly greater than 1, consistent with the gradual PTS reduction suggested by Ornstein-Uhlenbeck modeling (Fig. 3a) and ancestral state reconstructions (Fig. 4). Reduced PTS indices may reflect allometric constraints, as larger taxa tend to have lower PTS indices (Supplementary Tables S5–S6) and there have been independent increases in body mass within several primate lineages^25,29^.

## Discussion

Our finding that the highest PTS indices are observed in euarchontan species that habitually grasp vertical supports provides evidence for the hypothesis that the PTS can function as a cam mechanism when hypertrophied. The effect of the cam mechanism would be most pronounced in highly dorsiflexed foot postures typical of those observed in primates while grasping vertical supports^17,30,31^. However, the interpretation of PTS hypertrophy as a feature related to pedal grasping efficiency is complicated by the strong correlation between vertical support use and leaping behaviors in extant primates^14^, which makes it difficult to disentangle features that improve leaping performance from those that improve pedal grasping efficiency. Indeed, despite the ambiguity of the functional significance of PTS hypertrophy, several authors have linked PTS development with leaping^1,2^. Feasibly, the PTS cam mechanism could function as a power amplifier by storing elastic energy in the flexor fibularis muscle during ankle dorsiflexion, similar to the power amplifying properties of the vastus aponeurosis during knee flexion in galagos^32^.

Two sets of observations provide indirect arguments against the possibility that the PTS cam mechanism functions as a power amplifier during leaping. First, while the flexor fibularis could potentially plantarflex the foot at the ankle, primate electromyography studies^33,34^ show the muscle is most active during grasping. PTS hypertrophy likely impacts the primary action of the flexor fibularis muscle more than any auxiliary actions. Second, given tarsiers’ proficiency for leaping and grasping vertical supports^14–16^, it is counterintuitive that tarsiers do not exhibit a level of PTS hypertrophy comparable to other taxa with similar positional behaviors, such as indriids and galagids. Presumably a feature that functions as a power amplifier during leaping would also be advantageous for tarsiers. However, the modest PTS hypertrophy seen in tarsiers is more readily explained by differences in musculature associated with pedal grasping than differences in leaping, as tarsiers exhibit a relatively smaller flexor fibularis muscle^35^ and more developed intrinsic musculature within their foot than other strepsirrhines^36^. While it remains possible that the PTS cam mechanism functions as a power amplifier, the relatively modest PTS in tarsiers and the activation pattern of the flexor fibularis make the possibility less likely than the relationship with improved pedal grasping efficiency proposed here.

Provided PTS hypertrophy increases pedal grasping efficiency, it is important to recognize that the exact mode by which efficiency increases cannot be deduced from the feature’s distribution. Based on research of pedal grasping in other vertebrates, there are three possible alternatives for how the PTS could increase grasping efficiency.

1. *Translation of the tendon induces additional digital flexion* (Fig. 2). This mode would be analogous to the automatic digital flexor mechanism (ADFM) proposed to exist in some birds^37–40^. The ADFM purportedly enhances pedal grasping through passive flexion of the digits as the hindlimb flexes and the tendons of the extrinsic digital flexors (flexor hallucis longus and flexor digitorum longus) are drawn taut around the intertarsal joint. While dorsiflexion of the intertarsal joint in bird cadavers does induce digital flexion^40^, this mechanism has not been observed in experimental work with pigeons and crows^41^ or starlings^42^. The additional distance created during dorsiflexion may not exceed the passive excursion length of the muscle^41,42^, so that the muscles simply stretch, rather than induce digital flexion in birds. For primates, it is possible that the flexor fibularis could stretch to accommodate the additional travel distance caused by the PTS cam. However, when clinging to vertical supports, primates use deep crouching postures^30^ with more acute ankle joint angles (~56° for six strepsirrhine species^31^) than those observed at the intertarsal joint in starlings during perching^42^ (~120°). Furthermore, species with higher PTS indices such as *Propithecus verreauxi* (mean PTS index = 1.22) exhibit more acute ankle angles than species with lower PTS indices such as *Nycticebus coucang* (mean PTS index = 0.71) while vertical clinging (~39° and ~89° respectively)^31^. It remains possible that increased dorsiflexion in primates, combined with the PTS cam mechanism, causes tendon travel to exceed the distance achievable through passive excursion of the flexor fibularis, leading to digital flexion.
2. *Proximal translation of the tendon permits reduced muscular activity*. For extant primates and their close fossil relatives, the benefit of the PTS may be analogous to the tendon-locking mechanisms (TLM) that have convergently evolved along the tendons of pedal digital flexors in birds^43^, bats^44,45^, dermopterans^45^, and some climbing rodents^46^. TLMs are ratchet-like arrangements between the digital flexor tendons and flexor sheathes that can maintain a degree of digital flexion without constant muscular effort^43^. While all examined primates lack TLMs^45^, PTS hypertrophy may confer a similar benefit while grasping. This alternative could explain results from primate electromyography studies^33,34^ which do not detect increased flexor fibularis activity on vertical supports relative to horizontal substrates.
3. *The PTS cam mechanism shifts the flexor fibularis to a more efficient position on its length-tension curve*. This alternative is best supported by experimental work showing that above-branch perching does not involve digital flexion in several bird species^41,42^, thus questioning the existence of the ADFM. Rather, these authors suggest that the additional distance created during dorsiflexion of the foot could increase grasping efficiency by shifting muscles to a more efficient position of their length-tension curves. For primates, the PTS cam mechanism could increase grasping efficiency in a similar manner, leading to the prediction that differences in PTS hypertrophy correlate with differences in flexor fibularis length-tension curves across primates. This alternative could explain the widespread convergence of low PTS indices (reflecting deep flexor fibularis grooves) across primates (e.g., lorisids, atelids, *Pongo*, adapines, caenopithecines, and several subfossil lemurs) (Fig. 3). If these taxa habitually use hallucal grasps over a large range of ankle joint angles, flexor fibularis may already be in an optimal part of the length-tension curve, and it would be beneficial to minimize change to the tendon path length throughout joint excursion by keeping the tendon as close to the joint axis as possible.

Although the specific biomechanical mechanism by which PTS hypertrophy increases pedal grasping efficiency remains an open question, each alternative generates specific and testable predictions, so they can be investigated experimentally. It also is important to note additional characteristics of primate tali could increase or decrease the PTS cam effect. For example, we use a static model of the talocrural joint axis that reflects the curvature of the lateral tibial facet of the talus, rather than a dynamic model reflecting interactions between the distal tibia and talar trochlea. Dynamic modeling could potentially change expression of the cam mechanism. Additionally, dorsiflexion of the primate foot involves a degree of medial rotation of the talus^8,9^, so that a lateral position for the flexor fibularis groove^47,48^ or “twisting” of the PTS^35^ could enhance the cam effect. These additional factors could be incorporated into future studies.

While the specific mode is not resolved by this study, the evolution of the PTS and its implications for the adaptive origins of crown primates are less ambiguous. Both ancestral state reconstruction (Fig. 4) and SURFACE analyses (Fig. 3) reveal rapid development of the PTS during crown primate origins. The delta model favored by ancestral state reconstruction suggests that PTS hypertrophy occurred rapidly at the base of crown primates and then stabilized, as expected with an adaptive radiation^49^. These results suggest that the lineage leading to the ancestral crown primate experienced strong selective pressure to maintain pedal grasps in dorsiflexed foot postures. Since these foot postures are most pronounced during vertical support use^17,30,31^, the high PTS indices observed among Eocene primates and reconstructed for the ancestral crown primate strongly suggest that vertical support use was important selective component of positional behavior in the lineage leading to the ancestral crown primate.

Overall, our results align well with scenarios of crown primate origins that emphasize vertical supports^14,50^ or lemuriform-like positional behaviors^51^ that rely on strong hallucal grasps. Vertical support use in the ancestral crown primate is supported by other quantitative analyses of euarchontan tali^48,52,53^ and potentially the extremely elongated manual digits of early crown primates^54^. The prevalence of vertical postures among other euarchontans^19–21^ strongly suggests these postures were an inherited component of the ancestral crown primate’s positional behavior. Thus, PTS hypertrophy would not reflect a change in positional behavior in the lineage leading to the ancestral crown primate but would serve as a novel mechanism for maintaining a reliance on vertical supports.

## Methods

### Sample

The sample includes 388 individuals representing 126 extant and extinct euarchontan species (Supplementary Data S1 and S2). Institutional abbreviations are included in the Supplementary Material. All measurements were taken on 3D digital surface models generated with a variety of scanning modalities. Documentation for each specimen can be found on Morphosource.org^55^, an online repository for 3D data.

### Measurement protocol

To model the posterior trochlear shelf as a cam, we calculated a ratio of two linear measurements (Fig. S1b). These measurements capture (1) the radius of the cam’s base circle, representing the distance between the axis of the talocrural joint and the articular surface of the lateral tibial facet, and (2) the distance between the camshaft and the follower, representing the maximum distance between the axis of the talocrural joint and the flexor fibularis (=flexor hallucis longus) tendon. All measurements were taken in Geomagic Studio^56^.

To calculate the radius of the cam’s base circle, we first highlighted the lateral tibial articular facet (LTF) using the selection tool in Geomagic Studio (Fig. S1b). Extension of the LTF onto the talar neck was excluded from the selection to ensure the base circle followed the curvature of the trochlea. Next, the talocrural joint was modeled using the best-fit cylinder function in Geomagic (Features- > Cylinder- > Best-fit- > do not contact feature), with the axis of the best-fit cylinder representing the axis of the talocrural joint. The radius of the cylinder was recorded (“Radius” in Supplementary Data S1 and S2).

To calculate the distance between the axis of the cam and the follower, we placed a landmark in the groove for the flexor fibularis (FFG). In nearly all examined specimens, FFG was saddle-shaped: concave mediolaterally and convex dorsoplantarly. In these cases, a landmark was placed at the saddle point of the FFG after rotating the talus so that the main axis of the FFG was orthogonal to the viewing plane, as in Yapuncich *et al*.^48^. The FFG of one *Avahi* specimen (USNM 83652) was convex mediolaterally, so the landmark was placed at the point of maximal curvature in both directions. We then measured the distance between this landmark and the best-fit cylinder (“Groove to Cylinder” in Supplementary Data S1 and S2). Negative distances were possible if the landmark was within the best-fit cylinder. Radius and Groove to Cylinder were summed as a measure of the distance between the joint axis and the FFG (“Axis to Groove” in Supplementary Data S1 and S2).

Our metric for quantifying the size of the cam was generated from the measurements Axis to Groove and Radius: PTS Index = Axis to Groove/Radius. This index is dimensionless, with values > 1 representing strong development of the PTS, values < 1 representing no development of the PTS, and values = 1 indicating that the saddle point of the FFG lies on curvature ascribed by the lateral tibial facet. For certain analyses (see below), the PTS index was natural-log transformed after computation. Measurement protocol and calculation of the PTS index are illustrated in Supplementary Fig. S1b. Based on qualitative descriptions of the PTS in fossil primates^23,57^, we are confident that the PTS index captures the relevant morphology.

### Statistical analyses

We performed phylogenetic generalized least squares (PGLS) regressions, ordinary least squares (OLS) regressions, ANOVAs, one sample t-tests, and principal component analyses (PCA). All statistical analyses were conducted using species means (Supplementary Data S2) and protocols have been described in previous publications^48,53,58^. PGLS regressions were performed in R with the caper package^59^. OLS regressions, ANOVAs, one sample t-tests, and PCAs were performed in PAST 3.07^60^. Values for all analyzed variables are available for individuals in Supplementary Data S1 and for species means in Supplementary Data S2. Summary statistics for PTS indices and component measurements are available in Tables S1–S2.

### Phylogenetic tree construction

For PGLS regressions of extant taxa, the phylogenetic tree (Tree S1) was downloaded from the 10 K Trees^61^ and edited in Mesquite^62^ to include non-primate euarchontans. Branch lengths for dermopterans and scandentians came from Janečka *et al*.^63^ and Roberts *et al*.^64^ respectively. For regressions with fossil taxa, we used two different tree topologies: Tree S2, a supertree based largely on Gunnell *et al*.^65^, which recovers polyphyly among Adapiformes, and Tree S3, a modified tree from Yapuncich *et al*.^48^ (with the addition of *Donrussellia provincialis*), which positions all adapiforms as basal strepsirrhines and omomyiforms as basal tarsiiforms. The process for generating these trees and nexus files of the trees themselves are available in the Supplementary Material.

### Ancestral state reconstruction

Ancestral reconstructions of PTS indices were carried out using BayesTraits v3^66.^ We used the stepping-stone sampler to estimate log marginal likelihoods under both random walk and directional models, and, using comparisons of log Bayes Factors, tested whether addition of phylogenetic scaling parameters (delta, kappa, lambda) to either model provided positive evidence (i.e., log Bayes Factors of >2) for a more likely model of evolution. For each combination of random walk model + scaling parameter and directional model + scaling parameter, we ran two simultaneous stepping-stone analyses, with 1000 stones; each stone was run for 10,000 generations. Similar estimated marginal likelihoods from each independent run (mean difference of 0.06 between the estimated marginal likelihoods of paired runs) suggested that the number of generations was sufficient to allow for meaningful Bayes Factor comparisons. Ancestral reconstructions employed the model files from MCMC runs of the data with random walk model + delta parameter and random walk model + kappa combinations (i.e., the model + scaling parameter combinations that yielded the highest marginal likelihoods estimated by the stepping-stone sampler) for 20,050,000 generations (first 50,000 generations discarded as burn-in). Ancestral reconstructions were also run for 20,050,000 generations, with two independent runs, from which means for each ancestral reconstruction were calculated. Means and 95% highest posterior densities for ancestral reconstructions were calculated in Tracer v1.6.0^67^.

### SURFACE analyses

We used the *surface* package^68^ in R^69^ to detail the pattern of diversification for the PTS index within the euarchontans. Given continuous phenotypic data, the SURFACE function fits an Ornstein-Uhlenbeck (OU) evolutionary model to a given phylogeny^70^. Starting from an initial model with a single adaptive optimum (ϴ), SURFACE uses stepwise model selection to map additional adaptive optima to the phylogeny. These additional optima are interpreted as new adaptive regimes, reflecting shifts in the selective pressures faced by members of a particular lineage. Once a maximally complex model is estimated, SURFACE then evaluates whether model fit improves when different regimes are combined and share an adaptive optimum. Lineages that share an adaptive optimum are considered convergent.

Simulation studies comparing the accuracy of SURFACE and ℓ1ou^71^, another phylogenetic method for detecting evolutionary shifts, have demonstrated that SURFACE is prone to overfitting a tree with regime shifts^71^. This is largely due to SURFACE’s use of the Akaike information criterion to evaluate model goodness-of-fit^72^. However, SURFACE is preferred in analyses that included fossils, as it can run with non-ultrametric trees. To reduce the likelihood of recovering false positives, we conducted our analyses using modified versions of the phylogenies used for PGLS regressions and ancestral state reconstruction. In the first version, we consolidated all species to the genus level (except for *Tarsius*) to remove short branches. Genus-mean PTS indices were calculated as averages weighted by species representation. The second version was created after the initial SURFACE analyses identified several fossils with taxon-specific adaptive optima (e.g., *Purgatorius*, *Donrussellia*, and *Australopithecus*). In the second version, we removed all genera with taxon-specific adaptive regimes. For the third version, we resolved the polytomy at the base of euarchontans between scandentians, the paromomyid plesiadapiform *Ignacius*, and other plesiadapiforms by removing all plesiadapiforms (so that scandentians are the most basal members of our phylogenetic sample). Although relationships between the three extant euarchontan orders are not fully resolved^73,74^, a sister-taxon relationship between primates and dermopterans is supported by molecular analyses^63,75^.

As with other phylogenetic analyses, we evaluated two different tree topologies, one based on the topology recovered by Gunnell *et al*.^65^ (modified from Tree S2), and a more “traditional” topology that positions notharctids as basal strepsirrhines (modified from Tree S3) for all versions. Regime shifts and adaptive optima of the SURFACE analyses are presented in Tables S17–S21 and further detail is presented in the Supplementary Material.

## Supplementary information

Supplementary Material

Database S1

Database S2

## Data Availability

The authors declare that all data supporting the findings of this study are available within the paper and its supplementary information files. Digital surface models measured for this study are available at http://morphosource.org.
