# Supplementary material for: Vertical support use and primate origins
Source: Sci Rep. 2019 Aug 26;9:12341. doi: 10.1038/s41598-019-48651-x (PMC6710261; doi:10.1038/s41598-019-48651-x)
Supplement: Supplementary file 1 — Supplementary Material [file 41598_2019_48651_MOESM1_ESM.pdf]

Yapuncich et al. 2019 – Vertical support use and primate origins

Supplementary Information

Gabriel S. Yapuncich, Henry J. Feng, Rachel H. Dunn, Erik R. Seiffert, Doug M. Boyer

Corresponding author: Gabriel S. Yapuncich
Email: gabriel.yapuncich@duke.edu

Contents

Supplementary Materials and Methods
- Institutional abbreviations ……………………………………………………….. 3
- Phylogenetic tree construction ………………………………………………… 3

Supplementary Results
- Scaling relationships of PTS index and body mass …………………………… 6
- Correlations between PTS index and other talar metrics ……………………… 6
- ANOVA and one-way t-tests ……………………………………………………. 6
- Principal component analyses of talar metrics …………………………………7
- Ancestral state reconstruction ……………………………………………………9
  - PTS hypertrophy in the hominin lineage……………………………………… 9
  - Tree S1 ………………………………………………………………….... 10
  - Tree S2 ………………………………………………………………….... 13
  - Tree S3 …………………………………………………………………… 17
- SURFACE analyses ……………………………………………………………… 21

Supplementary Figures
- Fig. S1. Cam mechanism schematic and measurement protocol …………………... 24
- Figs. S2-S4. Comparative plates ……………………………………………………27
- Fig. S5. Boxplots of group means for PTS index ………………………………... 31
- Fig. S6. Boxplots of species means for PTS index ……………………………………32
- Fig. S7. Principal components analysis of four talar variables ………………………...34
- Fig. S8. Principal components analysis of five talar variables ……………………... 35
- Fig. S9. Phylogenetic tree (Tree S2) with ASR node numbers ……………………….. 36
- Fig. S10. Boxplots of estimated adaptive optima (ϴ) for the PTS index ……………... 37
- Fig. S11-S14. Adaptive regimes identified with SURFACE………………………….. 38

Supplementary Tables
- Table S1. Species mean PTS measurements in extant taxa …………………………… 42
- Table S2. Species mean PTS measurements in extinct taxa …………………………...44
- Table S3. PGLS and OLS regressions of PTS index and body mass ………………… 45
- Table S4. PGLS regressions of PTS index components and body mass ……………… 45
- Table S5. PGLS regressions of PTS index, components, and trochlear width ……….. 45
- Table S6. PGLS regressions for PTS index and other talar metrics …………………... 46

Table S7. ANOVA and post hoc comparison tests for PTS index 46
Table S8. PTS index one-way t-tests 47
Table S9. Summary of four variable PCA 47
Table S10. PC correlations for four variable PCA 47
Table S11. Summary of four variable PCA 48
Table S12. PC correlations for four variable PCA 48
Table S13. Delta model ASR summary statistics 49
Table S14. Kappa model ASR summary statistics 51
Table S15. Estimated marginal likelihoods for ASR models 53
Table S16. Contrasts of ASR values recovered with Tree S2 and Tree S3 54
Table S17. SURFACE summary statistics 55
Table S18. SURFACE regime shifts and adaptive optima for all taxa 56
Table S19. SURFACE regime shifts and adaptive optima without singular lineages ... 57
Table S20. SURFACE regime shifts and adaptive optima without plesiadapiforms 58
Table S21. SURFACE regime shifts and adaptive optima for extant taxa 59

References 61

<u>**Other supplementary materials for this manuscript**</u>

Database S1. Individual specimen data table
Database S2. Species mean data table

Supplementary Materials and Methods

Institutional Abbreviations. AMNH, American Museum of Natural History, New York, NY, USA; CGM, Egyptian Geological Museum, Cairo, Egypt; DLC, Duke Lemur Center, Durham, NC, USA; DPC, Duke Lemur Center Division of Fossil Primates, Durham, NC, USA; CM, Carnegie Museum of Natural History, Pittsburgh, PA, USA; GU, H.N.B. Garhwal University, Srinagar, Uttarakhand, India; HTB, Cleveland Museum of Natural History, Hamann-Todd non-human primate osteological collection, Cleveland, Ohio, USA; ISE-M, Institut des Sciences de l'Evolution de Montpellier, Montpellier, France; IRSNB, Institut Royal des Sciences Naturelles de Belgique, Brussels, Belgium; IVPP, Institute of Vertebrate Paleontology and Paleoanthropology, Chinese Academy of Sciences, Beijing, China; MACN, Museo Nacional de Ciencias Naturales, Buenos Aires, Argentina; MCZ, Museum of Comparative Zoology, Harvard University, Cambridge, MA, USA; MNHN, Muséum National d'Histoire Naturelle, Paris, France; NMB, Naturhistorisches Museum Basel, Basel, Switzerland; NMMP, National Museum of Myanmar Primates, Yangon, Myanmar; NMNH, Smithsonian Institution National Museum of Natural History, Washington, DC, USA; NYCEP, New York Consortium in Evolutionary Primatology, New York, NY, USA; SBU, Stony Brook University, Stony Brook, NY, USA; SDNHM, San Diego Natural History Museum, San Diego, CA, USA; UCM, University of Colorado Museum of Natural History, Boulder, CO, USA; UF, University of Florida, Florida Museum of Natural History, Gainesville, FL, USA; UM, University of Michigan, Ann Arbor, MI, USA; USGS, United States Geological Survey, Denver, CO, USA; UNSM, University of Nebraska Science Museum, Lincoln, NE, USA; USNM, United States National Museum, Smithsonian Institute, Washington, DC, USA.

Phylogenetic tree construction. To construct a composite supertree of living and extinct primates with divergence dates, we combined results from Bayesian tip-dating analyses of the matrices of 1) Gunnell et al. (1), a combined molecular/morphological dataset that includes several living and extinct strepsirrhines and haplorhines and non-primate euarchontan outgroups, with an emphasis on Paleogene species; 2) Herrera and Dávalos (2), a combined molecular/morphological dataset that includes all of the living and extinct crown strepsirrhines sampled for the current study, including subfossil species; and 3) Kay (3), a morphological dataset that includes all of the living and extinct platyrrhines that were sampled for our study. We attempted to integrate a tip-dating analysis of a matrix of notharctine adapiforms (4), but that analysis failed to produce a resolved "allcompat" tree, so we used the topology derived from Bayesian non-clock analysis of that matrix, with taxa separated by at least 1 million years. All Bayesian phylogenetic analyses were run in the MPI version of MrBayes 3.2.6 (5).

Bayesian tip-dating analysis of the matrix used by Gunnell et al. (1) provided the phylogenetic structure for non-primate euarchontans and most haplorhines aside from crown platyrrhines and crown catarrhines. We closely followed the methods of Gunnell et al. (1) when analyzing this matrix, but used the brlenspr=clock:uniform command instead of the brlenspr=clock:fossilization command and its associated parameters. The analysis was run for 75 million generations, with the first 10 million generations discarded as burn-in, and trees were summarized as an "allcompat" (majority-rule plus compatible groups) tree.

From the study of Herrera and Dávalos (2), which provided the topology for all living and extinct crown strepsirrhines, we chose to use the allcompat tree derived from analysis of their full morphological dataset with the fossilized birth-death prior on branch lengths. For the strepsirrhine nodes that overlap with those in the tree derived from analysis of the Gunnell et al. (1) matrix, those in the latter tree are, on average, 77.8% the age of those in the Herrera and Dávalos tree, consistent with Herrera and Dávalos' (recovery of a much more ancient "euprimate" node (68.04 Ma versus 60.11 Ma). To maintain consistency with the Gunnell et al. tree, we reduced the depth of all of the lemuriform branches in Herrera and Dávalos' tree by multiplying them by 0.778.

For our Bayesian tip-dating analysis of the platyrrhine matrix of Kay (3), we first converted polymorphisms into their own discrete states and characters with <6 states were ordered if they were ordered in Kay's analyses (characters with more states cannot be ordered due to software limitations). The value for the clockratepr parameter was derived from an R script written by S. Heritage, which uses the pathlengths from the allcompat tree output by a non-clock Bayesian analysis, and the ages of living and extinct taxa, to calculate a mean and standard deviation for the rate of character evolution (see Gunnell et al. [1] for additional details). The non-clock analysis that was run for this purpose imposed partial constraints that enforced a "molecular scaffold"; this analysis was run for 50 million generations, and the first 25% of sampled trees were discarded as burn-in. The resulting allcompat consensus was used to determine the values for the clockratepr parameter. In order to keep the platyrrhine divergences broadly consistent with Gunnell et al. (1) (which employed the molecular supermatrix of Springer et al. [6]), divergences within crown Atelinae, crown Callithricidae, crown Pitheciinae, and crown Catarrhini were fixed to fit the means of the four different divergence estimates for each node provided by Springer et al. (6), while all other nodes were free to vary. These nodes were selected because no fossil species in the Kay matrix (3) have been placed within these clades in recent parsimony or Bayesian phylogenetic analyses of morphological data. In addition, a truncated normal calibration was used for the age of the entire platyrrhine clade, based on the age of *Branisella* (27.2 Ma), the oldest platyrrhine in the Kay matrix (3).

We also ran a Bayesian tip-dating analysis of Gunnell's matrix (4) of Eocene notharctines. We assigned polymorphisms in the matrix intermediate states, maintained the same ordering scheme that was used by Gunnell (4), and ran a non-clock Bayesian analysis to obtain values for the clockratepr parameter (20 million generations, first 25% of sampled trees discarded as burn-in). The runs from the subsequent Bayesian tip-dating analysis of the matrix failed to converge, however, and did not produce a resolved allcompat tree, so we used the topology from the non-clock Bayesian analysis and used the '1-Ma rule' to assign divergence dates.

Other modifications to the resulting supertree include: 1) the divergence between *Ptilocercus* and *Tupaia* was based on the morphological clock-based divergence of *Ptilocercus kylin* from *Tupaia* (48.24 Ma); 2) *Ignacius* was placed into a polytomy with non-purgatoriid plesiadapiforms; 3) *Plesiadapis rex* and *Plesiadapis cookei* were grafted onto the *Plesiadapis* branch following Yapuncich et al. (7); 4) *Teilhardina brandti* was grafted mid-way along the branch between *Teilhardina belgica* and *Steinius*, following previous papers (7-9); 5) the omomyiform *Ourayia* was grafted onto the tree as the sister taxon of *Hemiacodon* following Tornow (10); 6) the species of *Eosimias* in the Gunnell et al. matrix (1) is *Eosimias centennicus*, but the tali measured

for this study are from older *E. sinensis*, so the tip of the terminal branch for *Eosimias* was placed at 45 Ma (11); 7) the hominoid-cercopithecoid divergence, placed at 21.7 Ma in the Gunnell et al. matrix (1), was placed at 25.2 Ma following Stevens et al. (12), and all internal branches separating extant crown catarrhines were lengthened by 1.162% to reflect this change; 8) *Australopithecus afarensis*, which was not sampled in any of the morphological matrices that were used to create the composite supertree, was given a 1-Ma terminal branch, placing it along the hominin stem lineage at 4.3 Ma; the same arrangement was used for the 1.7 Ma East Turkana hominins sampled here, which joined the hominin stem lineage at 2.7 Ma.

The resulting supertree is presented below as Tree S2. Because this tree recovers polyphyly among Adapiformes, we wanted to evaluate alternative topology that maintained monophyly of this group. To construct this alternative tree (Tree S3), we added *Donrussellia provincialis* to the tree used in Yapuncich et al. (7) following protocols detailed in that paper.

Supplementary Results

Comparative plates showing variation of PTS index among extant and extinct taxa are shown in Figs. S2-S4.

Scaling relationships of posterior trochlear shelf and body size. There were no significant relationships observed between body mass and the PTS index and significant autocorrelation was detected within all groups except lemuriforms (Table S3). Among the components of the PTS index, significant positive allometry was recovered between lnRadius and lnBM, which indicates that the base circle of the cam (i.e., the curvature of the lateral tibial facet) increases more quickly than body mass increases (Table S4). Essentially, the joint surface becomes flatter as body mass increases; this finding is consistent with previous assessments of allometric scaling of articular surfaces in primates (13, 14).

When trochlear width is used as a body size proxy, the PTS index exhibits a significant negative relationship, indicating that the cam effect decreases as body size increases. This finding is consistent with observed postural differences among euarchontans: larger-bodied taxa are more likely to employ above-branch quadrupedal or suspensory behaviors (15, 16), rather than postures that require habitually dorsiflexed feet. There were also significant relationships recovered between each of the component distances and trochlear width, but the slope of these regressions did not deviate from the expectations of isometry (Table S5).

Correlations between PTS cam size and other talar metrics. All regressions exhibited significant phylogenetic autocorrelation, as detected by Pagel's lambda. Only FFG Ellipse showed a significant (positive) correlation with the PTS index (Table S6). FFG Ellipse models the groove for the flexor fibularis as an ellipse by calculating the ratio of the semi-major (mediolateral) axis to the semi-minor (anteroposterior) axis (7). The significant positive correlation between these two variables indicates that the groove becomes mediolaterally wider and anteroposteriorly shallower as the PTS is more strongly developed. Given that the measurement protocol used to quantify the development of the PTS involved placing a landmark at the saddle point of the groove for the flexor fibularis, the significant positive correlation observed between the PTS index and FFG Ellipse is highly intuitive.

ANOVA and one-way t-tests. The average intraspecific range of the PTS index (0.17) is 14% of range observed across the entire sample (1.20). As assessed by ANOVAs and *post hoc* comparisons, there are significant differences between several groups (Table S7) at the level of species means. Among extant strepsirrhines, lorisids exhibit PTS indices that are significantly lower than other strepsirrhine groups (Figs. S5-S6, Table S7). Additionally, indriids exhibit PTS indices that are significantly higher than galagids. There is broad overlap among anthropoid groups, largely as a result of high variation within hominoids (*Pongo* as a very low PTS index, while *Homo* exhibits a high PTS index). Our sample of cercopithecoids, which exhibit high PTS indices, are the only examined anthropoid group that is statistically differentiated (from hominoids, atelids, and callitrichines) (Figs. S5-S6, Table S7).

In comparisons of all major euarchontan groups (Figs. S5-S6, Table S7), we consolidated all strepsirrhine and anthropoid OTUs that were not significantly different from all other OTUs in the initial comparisons. Strepsirrhines were represented by lorisids and non-lorisids, while all anthropoid groups were combined. In the euarchontan-wide analysis, the mean PTS index of non-lorisid strepsirrhines was significantly higher than that of all other groups except tarsiers. The mean PTS index of lorisids was significantly lower than that of all other groups except non-primates. Finally, *post hoc* differences reveal significant differences between tarsiers and non-primates.

Results of the one-way t-tests are shown in Table S8. The mean PTS indices for all strepsirrhine clades except lorisids are significantly greater than 1, indicating that the distance from the joint axis to the FHL groove is greater than the circle ascribed by the curvature of the lateral tibial facet (i.e., the PTS will function as a cam during dorsiflexion). The mean PTS index of lorisids is significantly less than 1, indicating that the distance from the joint axis to the FHL groove is less than the circle ascribed by the curvature of the lateral tibial facet (i.e., no cam effect). Tarsiers also exhibit a mean PTS index that is significantly greater than 1. Among anthropoids, atelids and callitrichines have mean PTS indices that are significantly less than 1, but there are no significant differences among any other anthropoids.

Principal component analyses of talar metrics. We used principal components analysis (PCA) to visualize the morphospace created within Euarchonta by the PTS index and several previously published metrics of talar morphology. Analyzed metrics include PTS index, fibular facet angle (FFA) (8), the first principal component of three metrics of medial tibial facet morphology (MTF PC1) (9), and the natural logs of the position (FFG Position) and depth (FFG Ellipse) of the flexor fibular groove (7). PCAs were conducted in PAST (17) with species mean values for 123 taxa; values can be found in Data S2. The ancestral crown primate is also represented in these analyses using the ASR values of the talar variables reported by Boyer and Seiffert (8), Boyer et al. (9), and Yapuncich et al. (7). Two PCAs were generated using the correlation matrices: the first PCA does not include FFG Ellipse, while the second does.

The four-variable PCA is shown in Fig. S7 and eigenvalues and explained variance are reported in Table S9. Together, the first two principal components explain 78.5% of the total variance. Loadings of talar metrics onto each principal component are reported in Table S10. The first principal component is strongly positively correlated with FFA (0.80) and FFG Position (0.77), while strongly negatively correlated with medial tibial facet morphology (-0.84). PTS index has a strong negative correlation (-0.90) with the second principal component; all the other variables should modest positive correlations with PC2.

The five-variable PCA is shown in Fig. S8 and eigenvalues and explained variance are reported in Table S11. The first two principal components explain 75.1% of the total variance. Loadings of talar metrics onto each principal component are reported in Table S12. The first principal component has a strongly negative correlation with MTF morphology (-0.83), and moderate positive correlations with the remaining four variables. The second principal component has a strong positive correlation with PTS index (0.69), a moderate positive correlation with FFG Ellipse, and moderate negative correlations with FFA (-0.55) and FFG Position (-0.55).

In both PCA plots, taxonomic groups are well differentiated from each other, continuing a trend of increasing discriminatory power as euarchontan talar morphology has been placed into a more comprehensive and quantitative framework (7-9).

Two important points are revealed by these principal component analyses. First, these talar metrics partition euarchontans in the manner expected from previous discussion of these features (18-20). The first principal components of both PCAs are strongly correlated with three talar metrics (fibular facet angle, medial tibial facet morphology, position of the flexor fibularis groove) and neatly divide strepsirrhine and haplorhine primates. Thus, these PCAs confirm the initial description by Gebo (20) that these three features contribute to the primary axis of talar morphological variation separating primate suborders. This is particularly evident in the loadings of the first PCA (Table S12). Subsequent research has used these features to determine the taxonomic affinities of enigmatic fossil crown primates including eosimiids (21, 22) and amphipithecids (23, 24). While the three features with the strongest loadings on the first PCs do not necessarily discriminate crown primates from other euarchontans, taxonomic separation along the second principal components suggest that development of the posterior trochlear shelf is useful for distinguishing crown primates from other euarchontans. Again, these results confirm early descriptions of the posterior trochlear shelf as a crown primate feature (25).

Second, the PCAs reveal the strong morphological similarities in the tali of the earliest crown primates. In both analyses, omomyiforms have positive loadings on PC1 (representing a more sloping fibular facet, an expanded medial tibial facet, and a more laterally positioned flexor fibularis groove) and strong negative loadings on PC2 (representing a strongly developed PTS cam) and plot closer to notharctids than tarsiers within these morphospaces. Though omomyiforms and notharctids are thought to represent the initial radiations of haplorhines and strepsirrhines respectively, they are not as distinctly partitioned as extant taxa by the set of talar features proposed by Gebo (20) to distinguish these groups. Indeed, reduced discriminatory ability should be expected close to the base of the order. This expectation has provided justification for several recent studies of talar morphology (7-9) to quantify these talar features in comparative samples that include other euarchontans. Fossil evidence suggests that the Haplorhini-Strepsirrhini suborder split occurred very closely in time with the origin of crown primates, so extending the framework established by Gebo (20) has the potential to reveal morphological changes in the ankle associated with crown primate origins.

These PCAs also confirm previous analyses or clarify outstanding issues for other fossil taxa. Dermopterans and plesiadapiforms (except *Carpolestes*) are well separated from crown primates along the second principal component in both analyses. Lorisids and the subfossil lemurs *Palaeopropithecus*, *Babakotia,* and *Megaladapis* exhibit PC2 values like those of non-crown primate taxa; this morphological similarity is also evidenced in the SURFACE analyses below. *Tupaia* and *Carpolestes* both exhibit low PC1 values and modest PC2 values, similar to the nail-bearing callitrichines. This similarity is most evident in Fig. S8 and is detected in the SURFACE analyses below. *Ptilocercus*, the pen-tailed tree shrew, has been suggested as a model for the positional behaviors of the ancestral crown primate (26-29); in both PCAs, *Ptilocercus* is more similar to the earliest crown primates than any other non-crown primate taxon.

When Yapuncich et al. (7) performed a similar PCA analysis without the PTS index, ASR values for ancestral crown primate plotted within the polygon containing other euarchontans (their Figure 12). In the talar metric PCA of Boyer et al. (30), *Donrussellia*, the earliest known adapiform, plotted in very similar position within the non-crown primate euarchontan polygon (their Figure 5a). Here, when the PTS index is included, both *Donrussellia* and the ancestral crown primate plot much more closely to other crown primates (and fall within the lemuriform polygon in Fig. S8). Compared to earlier analyses, the position of *Eosimias*, the earliest known anthropoid (21, 22), also changes substantially when the PTS index is included. In the PCA of Yapuncich et al. (7), *Eosimias* plotted within the non-crown primate euarchontan polygon; here, the taxon occupies a much more central position in both PCAs. These results reinforce the uniqueness of the PTS cam mechanism among crown primates, as the tali of all three taxa (the ancestral crown primate, *Donrussellia*, and *Eosimias*) become more crown primate-like when the PTS index is included.

Among other crown primate fossil taxa, the talar morphology captured by these metrics suggest that later occurring adapiforms (e.g., adapines and caenopithecines) are more similar to slow-climbing lorisids, confirming previous assessments of the positional behaviors of these taxa (31-33). Asiadapines, adapiforms from the early Eocene of India, are well-represented by postcrania and have been interpreted as generalized arboreal quadrupeds (34, 35). However, Boyer et al. (36) and Yapuncich et al. (7) noted some similarities in the talus to slow-climbing taxa such as lorisids. Here, particularly in Fig. S8, the asiadapines *Asiadapis* and *Marcgodinotius* are more similar to extant lemuriforms than lorisids (adapines plot between asiadapines and lorisids). This suggests that asiadapine tali are not particularly specialized for slow-climbing (at least in a manner homologous to extant lorisids). The results from the SURFACE analyses below complicate this interpretation but underscore that asiadapines exhibit a mosaic of postcranial adaptations and remain an important group for further study.

Ancestral state reconstruction. Ancestral states values are summarized in Table S13 for the delta model and Table S14 for the kappa model. Estimated marginal likelihoods for all models are presented in Table S15, and contrasts between values reconstructed with Tree S2 and Tree S3 with delta model are presented in Table S16. Text versions of all phylogenetic trees used in this study are provided on the following pages. Node numbers for Tree S2 are shown in Fig. S9.

PTS hypertrophy within the hominin lineage. The estimated node values of the PTS index increase substantially within the hominin lineage (Figure 4; Table S13; Table S14), a finding at odds with our functional interpretation of PTS hypertrophy as a mechanism to increase pedal grasping efficiency. The ASR values are driven by high PTS index values in the only two fossil hominin specimens included in this study (*Australopithecus afarensis* [PTS index = 1.35] and *Homo* sp. KNM ER 1464 [1.18]), so some caution is warranted when interpreting the results. Our hominin sample is not sufficient to thoroughly examine PTS development within the clade, but further study of this feature in a broader comparative sample of fossil tali would be enlightening.

It is possible that PTS hypertrophy has some functional advantages for bipedalism as well. Namely, the PTS cam mechanism could increase tension in the flexor fibularis tendon to concentrically flex (and thus shorten) the toes. Shorter effective digits would be better able to resist high bending moments induced by ground reaction forces during bipedal locomotion (37, 38). Electromyography of the flexor fibularis shows the muscle is most active during the middle of the support phase as the foot transitions from heel strike to toe-off (39). Furthermore, the lengths of the pedal intermediate and proximal phalanges are reduced in humans and fossil hominins relative to those of chimpanzees and gorillas (40), which may indicate that resisting bending moments was a strong adaptive pressure during the evolution of obligate bipedalism. It is notable that digits 4 and 5 are particularly reduced in humans (41), as the flexor fibularis (=flexor hallucis longus in human anatomy) typically does not insert on the lateral digits (42, 43), and thus could not be recruited to resist bending moments.

Tree S1. Phylogenetic tree of extant taxa used for body mass PGLS regressions (Table S3). To generate .nex file, paste the following text into a WordPad or TextEdit document and save with ".nex" extension.

```
#NEXUS

BEGIN TAXA;
	TITLE Untitled_Block_of_Taxa;
	DIMENSIONS NTAX=73;
	TAXLABELS
		Alouatta_caraya Aotus_azarae Aotus_infulatus Aotus_nancymaae Aotus_trivirgatus
Arctocebus_calabarensis Ateles_belzebuth Ateles_fusciceps Ateles_geoffroyi Avahi_laniger
Brachyteles_arachnoides Hoolock_hoolock Cacajao_calvus Callicebus_donacophilus Callicebus_moloch
Callimico_goeldii Callithrix_jacchus Callithrix_penicillata Callithrix_pygmaea Cebus_apella Cheirogaleus_major
Cheirogaleus_medius Chiropotes_sp. Daubentonia_madagascariensis Eulemur_collaris Eulemur_fulvus
Eulemur_mongoz Euoticus_elegantulus Galago_moholi Galago_senegalensis Galagoides_demidoff Gorilla_gorilla
Hapalemur_griseus Prolemur_simus Homo_sapiens Hylobates_lar Indri_indri Lagothrix_lagotricha Lemur_catta
Leontopithecus_rosalia Lepilemur_leucopus Loris_tardigradus Macaca_fascicularis Macaca_nemestrina
Microcebus_griseorufus Mirza_coquereli Nasalis_larvatus Nycticebus_coucang Otolemur_crassicaudatus
Pan_troglodytes Perodicticus_potto Pithecia_sp. Pongo_pygmaeus Presbytis_melalophos Propithecus_diadema
Propithecus_verreauxi Saguinus_midas Saguinus_mystax Saguinus_oedipus Saimiri_boliviensis Saimiri_sciureus
Symphalangus_syndactylus Tarsius_bancanus Tarsius_spectrum Tarsius_syrichta Trachypithecus_cristata
Trachypithecus_obscurus Varecia_variegata Cynocephalidae Tupaia_sp. Ptilocercus_lowii Phaner_furcifer
Eulemur_albifrons
	;

END;


BEGIN TREES;
	Title 'Trees from "FFGTree.nex"';
	LINK Taxa = Untitled_Block_of_Taxa;
	TRANSLATE
		1 Alouatta_caraya,
		2 Aotus_azarae,
		3 Aotus_infulatus,
		4 Aotus_nancymaae,
		5 Aotus_trivirgatus,
```

6 Arctocebus_calabarensis,
7 Ateles_belzebuth,
8 Ateles_fusciceps,
9 Ateles_geoffroyi,
10 Avahi_laniger,
11 Brachyteles_arachnoides,
12 Hoolock_hoolock,
13 Cacajao_calvus,
14 Callicebus_donacophilus,
15 Callicebus_moloch,
16 Callimico_goeldii,
17 Callithrix_jacchus,
18 Callithrix_penicillata,
19 Callithrix_pygmaea,
20 Cebus_apella,
21 Cheirogaleus_major,
22 Cheirogaleus_medius,
23 Chiropotes_sp.,
24 Daubentonia_madagascariensis,
25 Eulemur_collaris,
26 Eulemur_fulvus,
27 Eulemur_mongoz,
28 Euoticus_elegantulus,
29 Galago_moholi,
30 Galago_senegalensis,
31 Galagoides_demidoff,
32 Gorilla_gorilla,
33 Hapalemur_griseus,
34 Prolemur_simus,
35 Homo_sapiens,
36 Hylobates_lar,
37 Indri_indri,
38 Lagothrix_lagotricha,
39 Lemur_catta,
40 Leontopithecus_rosalia,
41 Lepilemur_leucopus,
42 Loris_tardigradus,
43 Macaca_fascicularis,
44 Macaca_nemestrina,
45 Microcebus_griseorufus,
46 Mirza_coquereli,
47 Nasalis_larvatus,
48 Nycticebus_coucang,
49 Otolemur_crassicaudatus,
50 Pan_troglodytes,
51 Perodicticus_potto,
52 Pithecia_sp.,
53 Pongo_pygmaeus,
54 Presbytis_melalophos,
55 Propithecus_diadema,
56 Propithecus_verreauxi,
57 Saguinus_midas,
58 Saguinus_mystax,
59 Saguinus_oedipus,
60 Saimiri_boliviensis,
61 Saimiri_sciureus,

62 Symphalangus_syndactylus,
63 Tarsius_bancanus,
64 Tarsius_spectrum,
65 Tarsius_syrichta,
66 Trachypithecus_cristata,
67 Trachypithecus_obscurus,
68 Varecia_variegata,
69 Cynocephalidae,
70 Tupaia_sp.,
71 Ptilocercus_lowii,
72 Phaner_furcifer,
73 Eulemur_albifrons;

TREE consensus_68species =
((((((((44:6.877996,43:6.877995):14.532379,((47:12.784647,(66:4.015007,67:4.015007):8.769641):0.523455,54:13.308102):8.102273):8.589626,((12:8.241196,(36:6.598361,62:6.598362):1.642834):11.36475,((32:8.652233,(35:6.17588,50:6.175879):2.476353):6.480222,53:15.132455):4.473491):10.394055):16.811821,(((1:14.76024,((7:2.029549,(8:1.493361,9:1.493361):0.536187):7.117843,(11:2.35377,38:2.35377):6.793621):5.612849):6.56106,((((2:0.471942,3:0.471942):3.116193,4:3.588135,5:3.588134):15.899387,(((16:13.232628,(19:7.02913,(17:0.880798,18:0.880798):6.148332):6.203497):1.797437,40:15.030065):0.682187,((57:8.361426,59:8.361427):1.302045,58:9.663472):6.048781):3.775269):0.605006,(20:18.569905,(60:2.120959,61:2.120959):16.448946):1.522623):1.228773):1.411478,(((13:2.274028,23:2.274028):7.368851,52:9.642879):9.753209,(14:3.913001,15:3.913001):15.483086):3.336691):24.079043):22.028211,((63:16.314276,65:16.314276):7.983376,64:24.297652):44.542381):4.162986,(((((((45:14.271122,46:14.271123):8.292266,(21:11.388139,22:11.388139):11.17525):5.865706,(72:25.689024,41:25.689024):2.740071):2.51681,(37:20.909032,(10:16.5026,(56:25.689024,55:6.555554):9.947046):4.406432):10.036873):2.318811,(((((26:0.762196,73:0.762196):1.168775,25:1.930972):2.222732,27:4.153705):10.865892,((33:8.200839,34:8.200838):1.079679,39:9.280517):5.739079):5.523206,68:20.542802):12.721914):17.859349,24:51.124065):11.612487,(((6:18.73455,51:18.73455):15.900706,(42:24.057527,48:24.057528):10.577729):3.364744,(((28:5.384501,(29:1.19471,30:1.19471):4.189791):7.979917,49:13.364418):3.766768,31:17.131185):20.868815):24.736553):10.266465):13.196981,69:86.2):1.7,(71:63.4,70:63.4):24.5)[%] [%] [% setBetweenBits = selected];

END;

Tree S2. Phylogenetic tree of extant and extinct taxa based on Gunnell et al. (1) used for talar feature PGLS regressions, ancestral state reconstructions, and SURFACE analyses (with genera as OTUs).

```
#NEXUS
[written Wed Nov 08 13:34:10 EST 2017 by Mesquite version 3.31 (build 859)]

BEGIN TAXA;
	TITLE Taxa;
	DIMENSIONS NTAX=123;
	TAXLABELS
		Ptilocercus_lowii Tupaia_sp Saxonella_sp Carpolestes_simpsoni Nannodectes_gidleyi
Plesiadapis_cookei Plesiadapis_rex Ignacius_graybullianus Purgatorius_sp Cynocephalidae Asiadapis_cambayensis
Marcgodinotius_indicus Anchomomys_frontanyensis Djebelemur_martinezi Adapis_parisiensis Leptadapis_magnus
Afradapis_longicristatus Caenopithecus_lemuroides Arctocebus_calabarensis Perodicticus_potto Loris_tardigradus
Nycticebus_coucang Euoticus_elegantulus Galagoides_demidoff Otolemur_crassicaudatus Galago_moholi
Galago_senegalensis Daubentonia_madagascariensis Megaladapis_sp Lepilemur_leucopus Phaner_furcifer
Cheirogaleus_major Cheirogaleus_medius Microcebus_griseorufus Mirza_coquereli Archaeolemur_edwardsi
Indri_indri Babakotia_radofilai Palaeopropithecus_sp Avahi_laniger Propithecus_verreauxi Propithecus_diadema
Varecia_variegata Lemur_catta Hapalemur_griseus Prolemur_simus Eulemur_mongoz Eulemur_collaris
Eulemur_albifrons Eulemur_fulvus Donrussellia_provincialis Cantius_ralstoni Cantius_abditus Cantius_trigonodus
Smilodectes_gracilis Notharctus_tenebrosus Notharctus_venticolus Teilhardina_belgica Teilhardina_brandti
Steinius_sp Absarokius_sp Tetonius_homunculus Anemorhysis_sp Shoshonius_sp Washakius_sp
Necrolemur_antiquus Hemiacodon_gracilis Ourayia_uintensis Omomys_carteri Tarsius_spectrum Tarsius_bancanus
Tarsius_syrichta Eosimias_sp Pondaungia Parapithecid Proteopithecus_sylviae Catopithecus_browni
Aegyptopithecus_zeuxis Macaca_fascicularis Macaca_nemestrina Presbytis_melalophos Nasalis_larvatus
Trachypithecus_cristata Trachypithecus_obscurus Hoolock_hoolock Hylobates_lar Symphalangus_syndactylus
Pongo_pygmaeus Gorilla_gorilla Pan_troglodytes Australopithecus_afarensis Homo_sp_KNM_ER_1464
Homo_sapiens Dolichocebus_gaimanensis Callicebus_donacophilus Callicebus_moloch Proteropithecia_neuquensis
Cebupithecia_sarmientoi Pithecia_sp Cacajao_calvus Chiropotes_sp Alouatta_caraya Brachyteles_arachnoides
Lagothrix_lagotricha Ateles_fusciceps Ateles_belzebuth Ateles_geoffroyi Cebus_apella Neosaimiri_fieldsi
Saimiri_boliviensis Saimiri_sciureus Aotus_trivirgatus Aotus_nancymaae Aotus_azarae Aotus_infulatus
Saguinus_mystax Saguinus_midas Saguinus_oedipus Leontopithecus_rosalia Callimico_goeldii Callithrix_pygmaea
Callithrix_jacchus Callithrix_penicillata
	;

END;


BEGIN TREES;
	Title 'Trees from "PTS_nexus_names.nex"';
	ID 015f9ce3cd791;
	LINK Taxa = Taxa;
	TRANSLATE
		1 Ptilocercus_lowii,
		2 Tupaia_sp,
		3 Saxonella_sp,
		4 Carpolestes_simpsoni,
		5 Nannodectes_gidleyi,
		6 Plesiadapis_cookei,
		7 Plesiadapis_rex,
		8 Ignacius_graybullianus,
		9 Purgatorius_sp,
		10 Cynocephalidae,
```

11 Asiadapis_cambayensis,
12 Marcgodinotius_indicus,
13 Anchomomys_frontanyensis,
14 Djebelemur_martinezi,
15 Adapis_parisiensis,
16 Leptadapis_magnus,
17 Afradapis_longicristatus,
18 Caenopithecus_lemuroides,
19 Arctocebus_calabarensis,
20 Perodicticus_potto,
21 Loris_tardigradus,
22 Nycticebus_coucang,
23 Euoticus_elegantulus,
24 Galagoides_demidoff,
25 Otolemur_crassicaudatus,
26 Galago_moholi,
27 Galago_senegalensis,
28 Daubentonia_madagascariensis,
29 Megaladapis_sp,
30 Lepilemur_leucopus,
31 Phaner_furcifer,
32 Cheirogaleus_major,
33 Cheirogaleus_medius,
34 Microcebus_griseorufus,
35 Mirza_coquereli,
36 Archaeolemur_edwardsi,
37 Indri_indri,
38 Babakotia_radofilai,
39 Palaeopropithecus_sp,
40 Avahi_laniger,
41 Propithecus_verreauxi,
42 Propithecus_diadema,
43 Varecia_variegata,
44 Lemur_catta,
45 Hapalemur_griseus,
46 Prolemur_simus,
47 Eulemur_mongoz,
48 Eulemur_collaris,
49 Eulemur_albifrons,
50 Eulemur_fulvus,
51 Donrussellia_provincialis,
52 Cantius_ralstoni,
53 Cantius_abditus,
54 Cantius_trigonodus,
55 Smilodectes_gracilis,
56 Notharctus_tenebrosus,
57 Notharctus_venticolus,
58 Teilhardina_belgica,
59 Teilhardina_brandti,
60 Steinius_sp,
61 Absarokius_sp,
62 Tetonius_homunculus,
63 Anemorhysis_sp,
64 Shoshonius_sp,
65 Washakius_sp,
66 Necrolemur_antiquus,

67 Hemiacodon_gracilis,
68 Ourayia_uintensis,
69 Omomys_carteri,
70 Tarsius_spectrum,
71 Tarsius_bancanus,
72 Tarsius_syrichta,
73 Eosimias_sp,
74 Pondaungia,
75 Parapithecid,
76 Proteopithecus_sylviae,
77 Catopithecus_browni,
78 Aegyptopithecus_zeuxis,
79 Macaca_fascicularis,
80 Macaca_nemestrina,
81 Presbytis_melalophos,
82 Nasalis_larvatus,
83 Trachypithecus_cristata,
84 Trachypithecus_obscurus,
85 Hoolock_hoolock,
86 Hylobates_lar,
87 Symphalangus_syndactylus,
88 Pongo_pygmaeus,
89 Gorilla_gorilla,
90 Pan_troglodytes,
91 Australopithecus_afarensis,
92 Homo_sp_KNM_ER_1464,
93 Homo_sapiens,
94 Dolichocebus_gaimanensis,
95 Callicebus_donacophilus,
96 Callicebus_moloch,
97 Proteropithecia_neuquensis,
98 Cebupithecia_sarmientoi,
99 Pithecia_sp,
100 Cacajao_calvus,
101 Chiropotes_sp,
102 Alouatta_caraya,
103 Brachyteles_arachnoides,
104 Lagothrix_lagotricha,
105 Ateles_fusciceps,
106 Ateles_belzebuth,
107 Ateles_geoffroyi,
108 Cebus_apella,
109 Neosaimiri_fieldsi,
110 Saimiri_boliviensis,
111 Saimiri_sciureus,
112 Aotus_trivirgatus,
113 Aotus_nancymaae,
114 Aotus_azarae,
115 Aotus_infulatus,
116 Saguinus_mystax,
117 Saguinus_midas,
118 Saguinus_oedipus,
119 Leontopithecus_rosalia,
120 Callimico_goeldii,
121 Callithrix_pygmaea,
122 Callithrix_jacchus,

123 Callithrix_penicillata;

TREE tree_1 =

((((1:48.24211,2:48.24211):19.50704,(3:5.4208538,(4:7.008804,(5:1,(6:3,7:1):2.0899932):3.4208538):1):3.3283262999999996):0.0,8:10.8121308):0.0,(9:2.022822,(10:62.18668,(((11:2.902362,12:2.902361):2.245213,((13:14.643401,14:8.779499000000001):2.7737,(((15:5.855566,16:5.822832):11.1308101,(17:9.460258,18:3.369313):8.699993):2.569207,((((19:13.93605,20:13.93605):15.97362,(21:16.29131,22:16.291308):13.61836):7.284126,(23:21.4,(24:15.47688,(25:9.971729,(26:1.24,27:1.24):8.731729):5.50515):5.923095):15.793821):13.016722000000001,(28:43.71649599999999,(29:30.09,((30:24.35,(31:22.27,((32:11,33:11):6.44,(34:11.12,35:11.12):6.32):4.83):2.08):2.46,((36:19.86234,(37:16.95,((38:12.94,39:12.94):3.1,(40:15.05,(41:8.35,42:8.35):6.7):0.99):0.91):2.91234):3.97256,(43:19.02,((44:10.93,(45:8.1,46:8.1):2.83):4.95996,(47:5.94,(48:3.36,(49:1.24,50:1.24):2.12):2.58):9.95):3.13):4.8149):2.9751):3.28):13.62652):6.494023):7.7960329999999995):0.8389107):0.8020849):0.4629504,(((51:3.162769,(52:1.690993,((53:1.7495,54:1):1,(55:4.625,(56:3.625,57:1):1):2.35):0.8738124):1.781058):0.9575645,(58:3.5346693,(59:3.15608935,(60:5.429974,(((61:3.760715,62:1.349415):1,63:2.93):1.285447,((64:1.7054008,65:5.727):3.5976,(66:17.602026,((67:1,68:2.8748):7.205935,(69:7.89576,(70:18.64,(71:6.84,72:6.84):11.8):34.2658):2.174949):0.7283733):1.135447):1.272311):0.3672465):0.27190405):0.27190405):0.3844415):0.3079055,(73:11.6913,(74:15.193781000000001,((75:17.778756,76:12.892187):3.215939,((77:7.090118,(78:5.163017,(((79:3.68,80:3.68):11.62,(81:7.77,(82:7.53,(83:1.88,84:1.88):5.65):0.24):7.53):9.9,((85:7.76,(86:6.05,87:6.05):1.71):12.41,(88:17.53,(89:9.33,(90:7.74,(91:4.3,(92:1,93:2.7):1.6):3.44):1.59):8.2):2.64):5.03):9.54438):6.792588):3.066181,(94:9.08,(((95:3.61,96:3.61):22.63,(97:6.9995,(98:6.745,(99:13.3,(100:6.4,101:6.4):6.9):6.74):2.31):3.89):1.19,((102:19.8,((103:9.9,104:9.9):1.4,(105:2.26,(106:1.48,107:1.48):0.78):9.04):8.5):6.24,((108:20.63,(109:2.07,(110:2.06,111:2.06):12.55):6.02):3.36,((112:3.9,(113:3.54,(114:0.47,115:0.47):3.07):0.36):18.19,((116:6.68,(117:5.51,118:5.51):1.17):7.22,(119:13.2,(120:11.3,(121:5.48,(122:0.71,123:0.71):4.77):5.82):1.9):0.7):8.19):1.9):2.05):1.39):2.12):15.0531):5.983788):3.041283):3.063109):3.128986):0.2901816):2.076183):4.446108):1.1163671);

END;

Tree S3. Phylogenetic tree of extant and extinct taxa used for ancestral state reconstructions and SURFACE analyses (with genera as OTUs). Modified from Yapuncich et al. (7).

```
#NEXUS
[written Tue Jul 31 21:07:49 EDT 2018 by Mesquite version 3.51 (build 898)]

BEGIN TAXA;
	TITLE Untitled_Block_of_Taxa;
	DIMENSIONS NTAX=123;
	TAXLABELS
		Ptilocercus_lowii Tupaia_sp Saxonella_sp Carpolestes_simpsoni Nannodectes_gidleyi Plesiadapis_cookei Plesiadapis_rex Ignacius_graybullianus Purgatorius_sp Cynocephalidae Asiadapis_cambayensis Marcgodinotius_indicus Anchomomys_frontanyensis Djebelemur_martinezi Adapis_parisiensis Leptadapis_magnus Afradapis_longicristatus Caenopithecus_lemuroides Arctocebus_calabarensis Perodicticus_potto Loris_tardigradus Nycticebus_coucang Euoticus_elegantulus Galagoides_demidoff Otolemur_crassicaudatus Galago_moholi Galago_sp Daubentonia_madagascariensis Megaladapis_sp Lepilemur_leucopus Phaner_furcifer Cheirogaleus_major Cheirogaleus_sp Microcebus_griseorufus Mirza_coquereli Archaeolemur_edwardsi Indri_indri Babakotia_radofilai Palaeopropithecus_sp Avahi_laniger Propithecus_verreauxi Propithecus_diadema Varecia_variegata Lemur_catta Hapalemur_griseus Prolemur_simus Eulemur_mongoz Eulemur_collaris Eulemur_albifrons Eulemur_fulvus Donrussellia_provincialis Cantius_ralstoni Cantius_abditus Cantius_trigonodus Smilodectes_gracilis Notharctus_tenebrosus Notharctus_venticolus Teilhardina_belgica Teilhardina_brandti Steinius_sp Absarokius_sp Tetonius_homunculus Anemorhysis_sp Shoshonius_sp Washakius_sp Necrolemur_antiquus Hemiacodon_gracilis Ourayia_uintensis Omomys_carteri Tarsius_spectrum Tarsius_bancanus Tarsius_syrichta Eosimias_sp Pondaungia Parapithecid Proteopithecus_sylviae Catopithecus_browni Aegyptopithecus_zeuxis Macaca_fascicularis Macaca_nemestrina Presbytis_melalophos Nasalis_larvatus Trachypithecus_sp Trachypithecus_obscurus Hoolock_hoolock Hylobates_lar Symphalangus_syndactylus Pongo_pygmaeus Gorilla_gorilla Pan_troglodytes Australopithecus_afarensis 'Homo_sp_KNM_ER_1464' Homo_sapiens Dolichocebus_gaimanensis Callicebus_donacophilus Callicebus_moloch Proteropithecia_neuquensis Cebupithecia_sarmientoi Pithecia_sp Cacajao_calvus Chiropotes_sp Alouatta_caraya Brachyteles_arachnoides Lagothrix_lagotricha Ateles_sp Ateles_belzebuth Ateles_geoffroyi Cebus_apella Neosaimiri_fieldsi Saimiri_boliviensis Saimiri_sciureus Aotus_sp Aotus_nancymaae Aotus_azarae Aotus_infulatus Saguinus_sp Saguinus_midas Saguinus_oedipus Leontopithecus_rosalia Callimico_goeldii Callithrix_pygmaea Callithrix_jacchus Callithrix_penicillata
	;

END;


BEGIN TREES;
	Title 'Trees from "PTS_tree_old.tre"';
	ID 0164f2f725bb1;
	LINK Taxa = Untitled_Block_of_Taxa;
	TRANSLATE
		1 Ptilocercus_lowii,
		2 Tupaia_sp,
		3 Saxonella_sp,
		4 Carpolestes_simpsoni,
		5 Nannodectes_gidleyi,
		6 Plesiadapis_cookei,
		7 Plesiadapis_rex,
		8 Ignacius_graybullianus,
		9 Purgatorius_sp,
		10 Cynocephalidae,
		11 Asiadapis_cambayensis,
```

12 Marcgodinotius_indicus,
13 Anchomomys_frontanyensis,
14 Djebelemur_martinezi,
15 Adapis_parisiensis,
16 Leptadapis_magnus,
17 Afradapis_longicristatus,
18 Caenopithecus_lemuroides,
19 Arctocebus_calabarensis,
20 Perodicticus_potto,
21 Loris_tardigradus,
22 Nycticebus_coucang,
23 Euoticus_elegantulus,
24 Galagoides_demidoff,
25 Otolemur_crassicaudatus,
26 Galago_moholi,
27 Galago_sp,
28 Daubentonia_madagascariensis,
29 Megaladapis_sp,
30 Lepilemur_leucopus,
31 Phaner_furcifer,
32 Cheirogaleus_major,
33 Cheirogaleus_sp,
34 Microcebus_griseorufus,
35 Mirza_coquereli,
36 Archaeolemur_edwardsi,
37 Indri_indri,
38 Babakotia_radofilai,
39 Palaeopropithecus_sp,
40 Avahi_laniger,
41 Propithecus_verreauxi,
42 Propithecus_diadema,
43 Varecia_variegata,
44 Lemur_catta,
45 Hapalemur_griseus,
46 Prolemur_simus,
47 Eulemur_mongoz,
48 Eulemur_collaris,
49 Eulemur_albifrons,
50 Eulemur_fulvus,
51 Donrussellia_provincialis,
52 Cantius_ralstoni,
53 Cantius_abditus,
54 Cantius_trigonodus,
55 Smilodectes_gracilis,
56 Notharctus_tenebrosus,
57 Notharctus_venticolus,
58 Teilhardina_belgica,
59 Teilhardina_brandti,
60 Steinius_sp,
61 Absarokius_sp,
62 Tetonius_homunculus,
63 Anemorhysis_sp,
64 Shoshonius_sp,
65 Washakius_sp,
66 Necrolemur_antiquus,
67 Hemiacodon_gracilis,

68 Ourayia_uintensis,
69 Omomys_carteri,
70 Tarsius_spectrum,
71 Tarsius_bancanus,
72 Tarsius_syrichta,
73 Eosimias_sp,
74 Pondaungia,
75 Parapithecid,
76 Proteopithecus_sylviae,
77 Catopithecus_browni,
78 Aegyptopithecus_zeuxis,
79 Macaca_fascicularis,
80 Macaca_nemestrina,
81 Presbytis_melalophos,
82 Nasalis_larvatus,
83 Trachypithecus_sp,
84 Trachypithecus_obscurus,
85 Hoolock_hoolock,
86 Hylobates_lar,
87 Symphalangus_syndactylus,
88 Pongo_pygmaeus,
89 Gorilla_gorilla,
90 Pan_troglodytes,
91 Australopithecus_afarensis,
92 'Homo_sp_KNM_ER_1464',
93 Homo_sapiens,
94 Dolichocebus_gaimanensis,
95 Callicebus_donacophilus,
96 Callicebus_moloch,
97 Proteropithecia_neuquensis,
98 Cebupithecia_sarmientoi,
99 Pithecia_sp,
100 Cacajao_calvus,
101 Chiropotes_sp,
102 Alouatta_caraya,
103 Brachyteles_arachnoides,
104 Lagothrix_lagotricha,
105 Ateles_sp,
106 Ateles_belzebuth,
107 Ateles_geoffroyi,
108 Cebus_apella,
109 Neosaimiri_fieldsi,
110 Saimiri_boliviensis,
111 Saimiri_sciureus,
112 Aotus_sp,
113 Aotus_nancymaae,
114 Aotus_azarae,
115 Aotus_infulatus,
116 Saguinus_sp,
117 Saguinus_midas,
118 Saguinus_oedipus,
119 Leontopithecus_rosalia,
120 Callimico_goeldii,
121 Callithrix_pygmaea,
122 Callithrix_jacchus,
123 Callithrix_penicillata;

TREE tree_1 =
((1:48.24211,2:48.24211):19.50704,(3:5.4208538,(4:7.008804,(5:1,(6:3,7:1):2.0899932):3.4208538):1):3.32832629
99999996,8:10.8121308,(9:2.022822,(10:62.18668,((((11:2.902362,12:2.902361):2.245213,((13:14.643401,14:8.77
9499000000001):2.7737,(((15:5.855566,16:5.822832):11.1308101,(17:9.460258,18:3.369313):8.699993):2.569207,
((((19:13.93605,20:13.93605):15.97362,(21:16.29131,22:16.291308):13.61836):7.284126,(23:21.4,(24:15.47688,(2
5:9.971729,(26:1.24,27:1.24):8.731729):5.50515):5.923095):15.793821):13.016722000000001,(28:43.7164959999
9999,(29:30.09,((30:24.35,(31:22.27,((32:11,33:11):6.44,(34:11.12,35:11.12):6.32):4.83):2.08):2.46,((36:19.86234,(
37:16.95,((38:12.94,39:12.94):3.1,(40:15.05,(41:8.35,42:8.35):6.7):0.99):0.91):2.91234):3.97256,(43:19.02,((44:10.
93,(45:8.1,46:8.1):2.83):4.95996,(47:5.94,(48:3.36,(49:1.24,50:1.24):2.12):2.58):9.95):3.13):4.8149):2.9751):3.28):
13.62652):6.494023):7.7960329999999995):0.8389107):0.8020849):0.2314752,(51:3.162769,(52:1.690993,((53:1.7
495,54:1):1,(55:4.625,(56:3.625,57:1):1):2.35):0.8738124):1.781058):1.3241645):0.2314752,((58:3.5346693,(59:3.
15608935,(60:5.429974,(((61:3.760715,62:1.349415):1,63:2.93):1.285447,((64:1.7054008,65:5.727):3.5976,(66:17.
602026,((67:1,68:2.8748):7.205935,(69:7.89576,(70:18.64,(71:6.84,72:6.84):11.8):34.2658):2.174949):0.7283733):
1.135447):1.272311):0.3672465):0.27190405):0.27190405):0.692347,(73:11.6913,(74:15.193781000000001,((75:1
7.778756,76:12.892187):3.215939,((77:7.090118,(78:5.163017,(((79:3.68,80:3.68):11.62,(81:7.77,(82:7.53,(83:1.88
,84:1.88):5.65):0.24):7.53):9.9,((85:7.76,(86:6.05,87:6.05):1.71):12.41,(88:17.53,(89:9.33,(90:7.74,(91:4.3,(92:1,93:
2.7):1.6):3.44):1.59):8.2):2.64):5.03):9.54438):6.792588):3.066181,(94:9.08,(((95:3.61,96:3.61):22.63,(97:6.9995,(9
8:6.745,(99:13.3,(100:6.4,101:6.4):6.9):6.74):2.31):3.89):1.19,((102:19.8,((103:9.9,104:9.9):1.4,(105:2.26,(106:1.48
,107:1.48):0.78):9.04):8.5):6.24,((108:20.63,(109:2.07,(110:2.06,111:2.06):12.55):6.02):3.36,((112:3.9,(113:3.54,(1
14:0.47,115:0.47):3.07):0.36):18.19,((116:6.68,(117:5.51,118:5.51):1.17):7.22,(119:13.2,(120:11.3,(121:5.48,(122:0
.71,123:0.71):4.77):5.82):1.9):0.7):8.19):1.9):2.05):1.39):2.12):15.0531):5.983788):3.041283):3.063109):3.128986):
0.2901816):2.076183):4.446108):1.1163671);

SURFACE analyses. Summaries of all SURFACE analyses are presented in Table S17. The range of adaptive regimes for select clades are shown in Fig. S10. Adaptive regimes are detailed in Tables S18-21 and plotted on the phylogenies in Figs. S11-S14.

Version 1 – all taxa included: Adaptive optimum and convergent clades are detailed in Table S18 and adaptive regimes are shown for both topologies in Fig. S11.

With the Gunnell et al. (1) topology, the PTS index adaptive optimum (ϴ) at the ancestral node is close to 1 (0.97). Major regime shifts throughout euarchontan evolutionary history include substantial decreases in ϴ for all plesiadapiform lineages (-6.41 for *Purgatorius*, 0.59 for *Carpolestes*, -0.59 for all other plesiadapiforms) and dermopterans (0.59). Scandentians, basal strepsirrhine taxa (asiadapines, *Djebelemur*, and *Anchomomys*), and most anthropoids share the adaptive optimum of the ancestral node. The adaptive optimum for PTS index increases among notharctids and most omomyiforms (1.43), while ϴ actually decreases in the strepsirrhine lineage (an optimum of 0.59 is shared by adapines, caenopithecines, lorisids, and *Megaladapis*). Galagids exhibit an increased adaptive optimum of galagids increases (1.20) and most extant lemuriforms converge toward the high ϴ of notharctids and omomyiforms (1.43). Within their respective lineages, *Daubentonia*, tarsiers, and three late-occurring omomyiform taxa (*Omomys, Hemiacodon*, and *Ourayia*) converge on the adaptive optimum of the ancestral node (0.97). The subfossil lemuriforms *Palaeopropithecus* and *Babakotia* share a very low PTS index adaptive optimum with *Pongo* (0.14). The convergence between these three large-bodied and potentially suspensory taxa remains constant across all SURFACE analyses.

Fewer regime shifts and convergent lineages are recovered with the "traditional" topology. The PTS index adaptive optimum at the ancestral node is 0.93, and is maintained throughout the tree, including scandentians, basal strepsirrhine taxa (notharctids, *Djebelemur*, and *Anchomomys*), galagids, *Megaladapis*, *Daubentonia*, and most haplorhines. Increased ϴ estimates are rare: only lemuriforms exhibit an increased adaptive optimum (1.39) that is well-estimated. There are several adaptive regimes with ϴ values lower than the ancestral node, including a convergent regime comprised of dermopterans, lorisids, adapines, caenopithecines, and *Carpolestes* (0.34). Asiadapines share a very low PTS index adaptive optimum with *Palaeopropithecus*, *Babakotia* and *Pongo* (-0.39), though this optimum is problematic since a negative PTS index is mathematically impossible[1] as neither Radius nor Axis to Groove can be negative values.

Extreme optima are estimated in both topologies, including those for *Purgatorius* (-6.41 with the Gunnell et al. [1] topology; -11.41 with the "traditional" topology), *Australopithecus* and *Donrussellia* (2.43; 3.40), and all other plesiadapiforms except *Carpolestes* (-0.59; -1.53). These optima fall well outside the observed range for the sampled taxa and justify the exclusion of these problematic lineages in subsequent versions.

---

[1]A negative PTS index would indicate that the axis of the talocrural joint was posterior to the saddle point of the groove for flexor fibularis (FFG), which may be biologically possible. Because our measurement protocol finds the absolute shortest distance between the saddle point and the cylinder fit to the joint surface, it would not distinguish those specimens with this morphology. However, when taking measurements, we observed no specimens for which the joint axis was posterior to the saddle point of the FFG. In all specimens, the joint axis was anterior to the saddle point of the FFG.

Version 2 – Fossil taxa with extreme taxon-specific regimes (Purgatorius*,* Donrussellia*, and* Australopithecus) *removed:* Adaptive optimum and convergent clades are detailed in Table S19 and adaptive regimes are shown for both topologies in Fig. S12.

With the Gunnell et al. (1) topology, the PTS index adaptive optimum at the ancestral node is much lower than those previously recovered (0.69) and is shared by all non-crown primate euarchontans (scandentians, dermopterans, and all plesiadapiforms). Lorisids and *Megaladapis* exhibit convergence with this adaptive regime, and palaeopropithecids (*Babakotia* and *Palaeopropithecus*) and *Pongo* share a lower adaptive optimum (0.33). All other crown primate groups have higher adaptive optima than the ancestral node. The highest optimum (1.35) is shared by most lemuriforms, notharctids, most omomyiforms, *Anchomomys*, and *Djebelemur*. Galagids have an adaptive optimum of 1.19. The remaining taxa (most anthropoids, tarsiers, *Omomys*, *Ourayia*, *Hemiacodon*, *Daubentonia*, adapines, and caenopithecines) share an adaptive optimum close to 1 (0.98). With this topology, there is only one problematic regime shift, occurring along a very short branch at the base of Haplorhini (Shift 8, $\Theta = 45.12$).

Using the reduced taxonomic sample, there are also fewer regime shifts and convergent lineages are recovered with the "traditional" topology. The PTS index adaptive optimum at the ancestral node is close to 1 (0.98) and is widely maintained throughout the tree (including scandentians, galagids, *Daubentonia*, *Djebelemur*, *Anchomomys*, notharctids, tarsiers, omomyiforms, and most anthropoids. Only lemuriforms exhibit an increased adaptive optimum (1.39). Dermopterans, lorisids, adapines, caenopithecines, *Carpolestes*, and *Megaladapis* all share a decreased adaptive optimum of 0.40. The remaining taxa fall into two regimes with poorly estimated adaptive optima: asiadapines, palaeopropithecids, and *Pongo* are convergent ($\Theta = -0.27$), while all plesiadapiforms except *Carpolestes* have an optimum of -1.23.

Version 3 – Plesiadapiforms removed: Adaptive optimum and convergent clades are detailed in Table S20 and adaptive regimes are shown for both topologies in Fig. S13.

The final version excluded all plesiadapiform taxa in order to resolve the at the base of Euarchonta. Only four non-convergent regimes are identified with the Gunnell et al. (1) topology. The ancestral node has an adaptive optimum of 0.98, which is maintained widely across euarchontans (scandentians, galagids, notharctids, omomyiforms, *Daubentonia,* and all haplorhines except *Pongo*). The only lineage with an increased adaptive optimum (1.29) are lemuriforms. Dermopterans, lorisids, adapines, caenopithecines, and *Megaladapis* converge toward an adaptive optimum of 0.41. Finally, asiadapines, palaeopropithecids, and *Pongo* share a problematic optimum of -0.24.

Seven regimes are identified with the "traditional" topology and no problematic (i.e., negative) optima estimated. As opposed to all other versions, the ancestral node for euarchontans has a high adaptive optimum for the PTS index (1.61); the microchoerine omomyiform *Necrolemur* converges to this high optimum. Non-primate euarchontans are members of separate convergent regimes with adaptive optima less than 1. Scandentians are convergent with callitrichines ($\Theta = 0.78$), and dermopterans are convergent with lorisids, adapines, caenopithecines, and *Megaladapis* (0.61). A regime shift occurs at the base of Primatomorpha (primates + dermopterans) ($\Theta = 1.11$). While there is another regime shift within the dermopteran lineage,

crown primates (including tarsiers, galagids, notharctids, most omomyiforms, *Djebelemur*, *Anchomomys*, and *Daubentonia*) maintain the higher optima of 1.11. As with other versions and topologies, palaeoprothecids and *Pongo* share a low adaptive optimum of 0.27 with asiadapines. Most anthropoids (except *Pongo* and callitrichines) have a PTS index optimum of 0.98, while most lemuriforms (except *Daubentonia* and *Megaladapis*) share an optimum of 1.24.

Version 4 – Extant taxa only: Adaptive optimum and convergent clades are detailed in Table S21 and adaptive regimes are shown in Fig. S14. Portions of these results are presented in Fig. 2a but are also presented here to facilitate comparison.

When considering extant taxa, there are no differences between the Gunnell et al. (1) and "traditional" topologies. There are four adaptive regimes identified with SURFACE. The ancestral node, scandentians, and dermopterans share an adaptive optimum of 0.74. A regime shift to a higher adaptive optimum (1.59) occurs at the ancestral primate node, which is maintained across most extant primates. *Pongo* has a unique (though problematic) adaptive regime (Θ = -1.58), while lorisids and *Callithrix* converge on an optimum of -0.08.

Summary of SURFACE analyses: In the main text, we presented results from the SURFACE analysis of extant taxa for simplicity (Fig. 2a). The regime shifts identified in the extant only analysis align very well with the ancestral state reconstruction (ASR) results (Fig. 3, Table S13), as the SURFACE analysis recovers a substantial increase in the PTS index adaptive optimum at the base of Primates (Fig. S14, Table S21). However, the inclusion of fossil taxa in SURFACE analyses adds complexity to the evolution of the feature within Euarchonta. When modeling both extant and extinct taxa, two factors suggest that Version 3 with the "traditional" topology should be the preferred model. First, this version does not estimate any optima that are outside the PTS values observed in our sample (Table S1-S2, Table S20), so all regimes seem reasonably estimated. Second, this version has the lowest AICc of all examined versions, indicating that it is the best-fit model (Table S17) (although we note that sample sizes are not equivalent across all versions).

The regimes identified by Version 3 with the "traditional" topology (Fig. S13, Table S20) align moderately well with the ancestral state reconstructions (Table S13), recovering a regime shift with an adaptive optimum of 1.11 at the node representing Primatomorpha (Dermoptera + Primates). Dermopterans exhibit another regime shift to a lower optimum (0.61), while notharctids, omomyiforms (except *Necrolemur*), tarsiers, galagids, *Anchomomys*, and *Djebelemur* maintain the higher optimum of the Primatomorpha node (Fig. S13).

The key difference between ASR and SURFACE results from Version 3 concerns the direction of change deep in the tree. ASR results estimate that the PTS index at the ancestral euarchontan node was quite low and subsequently increased (Fig. 3, Table S13). In contrast, the best-fit SURFACE model recovers a high adaptive optimum at the base of Euarchonta (1.61), with a shift to a reduced optimum at the Primatomorpha node (1.11). Optima are reduced independently in the lineages of Scandentia (0.78) and Dermoptera (0.61). Thus, the best-fit SURFACE model suggests the selective pressure to develop a pronounced posterior trochlear shelf decreased from the ancestral euarchontan node to the Primatomorpha node. Given the absence of PTS

development in any other mammalian group, we do not consider this scenario likely. Future analyses should include non-euarchontan outgroups to address issue.

Overall, SURFACE analyses detect regime shifts that decrease adaptive optima more frequently than shifts that increase optima, which may be due to widespread homoplasy of reduced PTS indices across Euarchonta. In all analyses, dermopterans, plesiadapiforms, lorisids, adapines, and caenopithecines exhibit (and often converge toward) adaptive optima substantially less than 1. Several taxa within this set have been compared favorably with one another by previous authors. Beard (44-46) and Bloch and Boyer (47) suggest some similarities in the postural behaviors of claw-bearing plesiadapiforms and dermopterans, though these similarities do not necessarily extend to locomotor behaviors such as mitten-gliding (29, 48-52). Based on comparisons of multiple postcranial elements, Dagosto (31, 53) and Gebo (54) have favorably compared the postural behaviors of lorisids and adapines. Similarities in tarsal morphology led Boyer et al. (33) to suggest that the caenopithecine *Afradapis* had a locomotor profile like those of extant lorises. Seiffert et al. (32) found phenetic similarities between the caenopithecine *Caenopithecus* and extant lorisids for both the talus and calcaneus.

In every analysis, palaeopropithecids and *Pongo* converge toward a very low (occasionally negative) optimum for the PTS index. Morphological similarities between these taxa have long been recognized, ranging from strongly curved proximal phalanges (55), lumbar vertebrae morphology (56), and long bone cross-sectional dimensions (57). SURFACE analyses conducted here lend further support to the hypothesis that palaeopropithecids were large-bodied suspensory taxa, with an overall positional behavior similar to *Pongo*.

Across all analyses, few regimes exhibit increased adaptive optima (Fig. S10). Among extant taxa, lemuriforms (except *Daubentonia*) consistently exhibit increased optima ranging from 1.24 to 1.43, while galagids often (but not always) have optima greater than 1 (0.93 to 1.2). Notharctids and omomyiforms share adaptive optima that are generally greater than 1 (0.93 to 1.43), similar to those of galagids. The only other taxa that consistently have optima greater than 1 are the adapiforms *Anchomomys* and *Djebelemur* (0.93 to 1.35).

Comparisons of optima estimated across SURFACE analyses reveal that the differences in the included sample and topology do not have a strong impact on the estimated adaptive optima for most groups (Fig. S10). Notable exceptions include asiadapines, which exhibit the most variation in their estimated adaptive optima, and plesiadapiforms, which were excluded from Version 3 in order to resolve the polytomy at the base of Euarchonta. It is likely that short branch lengths and shifting phylogenetic position explain the high variance in adaptive optima for both taxa.

Based on analyses of multiple postcranial elements, asiadapines have been reconstructed as generalized arboreal quadrupeds (34, 35), though some tarsal features are similar to those observed in slow-climbing taxa (7, 8). The four specimens examined for this study have modest PTS indices (ranging from 0.91 to 1.25; Table S2; Fig. S6) but the clade has adaptive optima that range from -0.39 to 2.30. It is possible that short branch lengths and shifting phylogenetic position may explain the variability in estimated adaptive optima for asiadapines. In the Gunnell et al. (1) topology, asiadapines are the most basal strepsirrhines, a position which is occupied by notharctids in the "traditional" topology. It is worth noting that this region of the phylogeny also

generates large contrasts when comparing estimated node values of the two versions of ancestral state reconstruction (Table S16). Using the Gunnell et al. (1) topology, SURFACE tends to estimate multiple regimes within the strepsirrhine lineage, and asiadapines are one of several regimes with high Θ values. In contrast, when asiadapines are bracketed by the taxa with high PTS indices (notharctids and *Djebelemur* + *Anchomomys*) in the "traditional" topology, their modest PTS indices necessitate very low optima (Versions 1 and 2). In the best-fit model (Version 3 with the "traditional" topology), asiadapines share a low adaptive optimum with palaeopropithecids and *Pongo*, suggesting strong selective pressures to reduce the PTS index with the group. Given the observed PTS index values for the group, asiadapines are quite distant from their adaptive optimum. Overall, the estimated Θ values of Version 3 are consistent with interpretations of asiadapines as generalized quadrupeds (34, 35), while remaining concordant with studies that have noted tarsal features that are shared with slow-climbing taxa (7, 8).

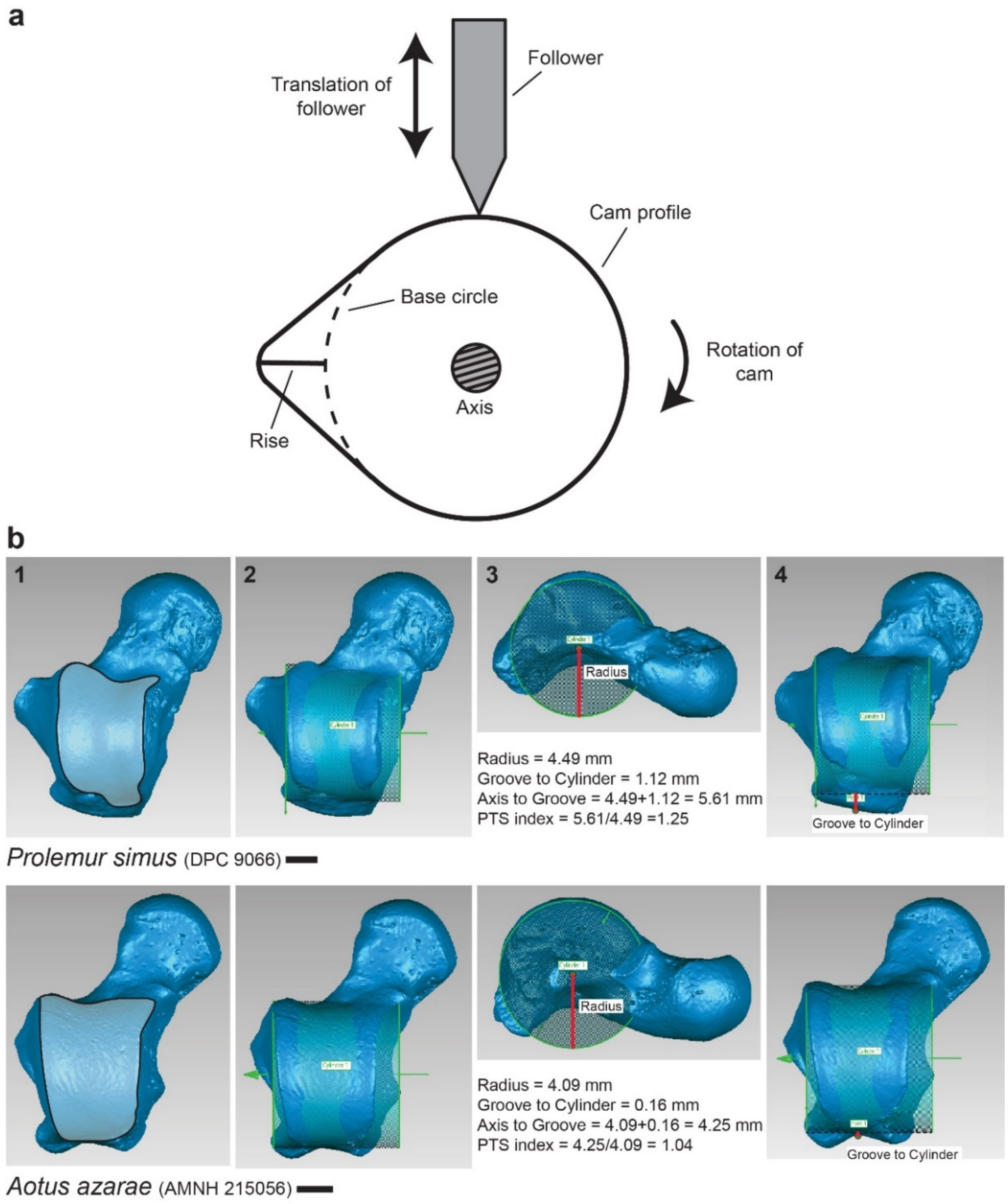

Fig. S1.

Schematic of cam mechanism (a) and measurement protocol (b) to quantify development of the posterior trochlear shelf in two primate taxa. 1) The articular surface of the lateral tibial facet is highlighted in Geomagic Studio. 2) As an approximation of the base circle of the cam, a best-fit cylinder is fit to the selected area. 3) The radius of the best-fit cylinder is calculated. 4) A landmark is placed at the saddle point of the groove for the tendon of the flexor fibularis muscle. The groove to cylinder distance is measured with the "measure distance to feature" function of Geomagic Studio. PTS index is calculated as the ratio of the distance between the groove and the axis of the base circle (=Radius + Groove to Cylinder) relative to the radius of the base circle (=Radius).

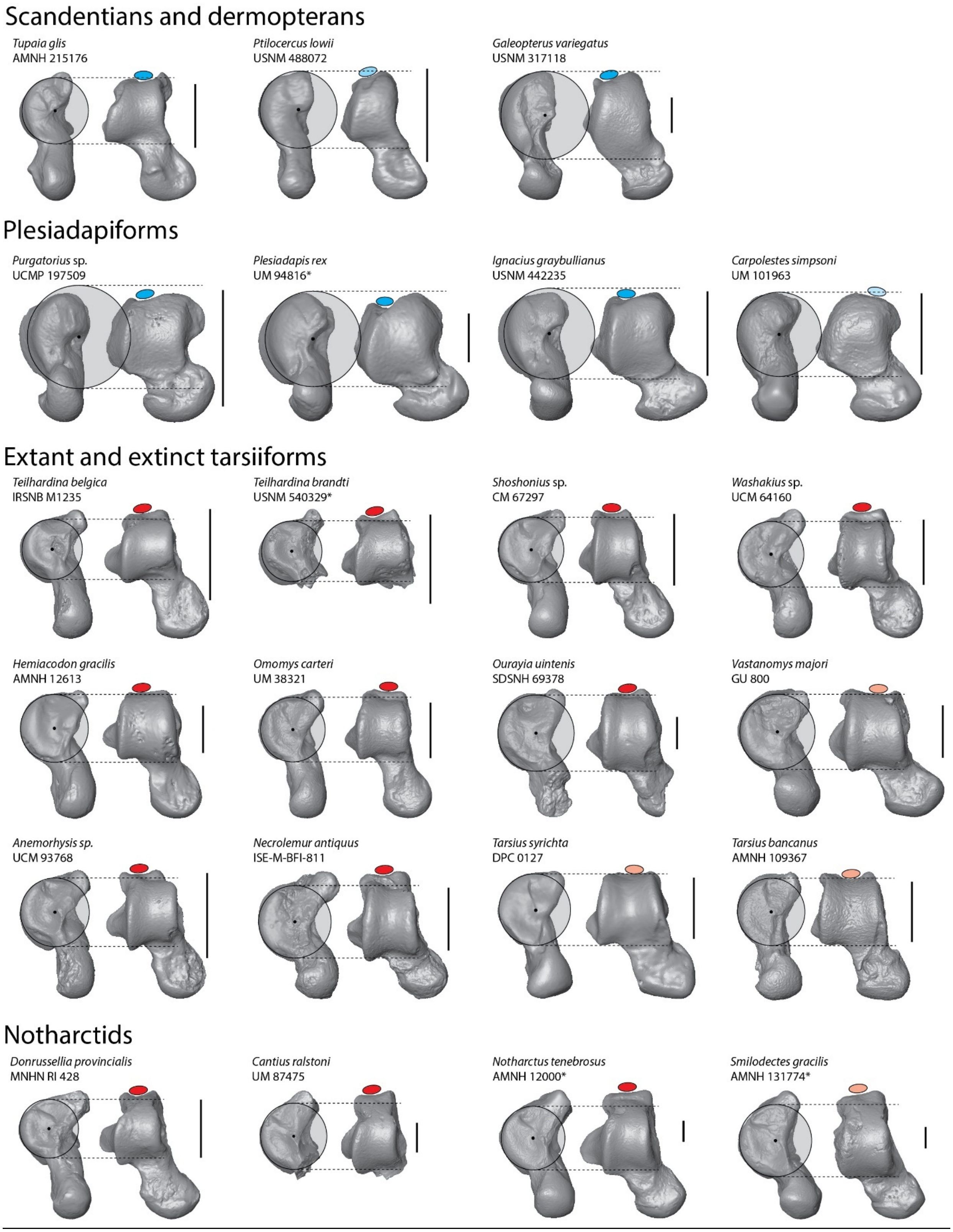
Scandentians and dermopterans
Tupaia glis
AMNH 215176
Ptilocercus lowii
USNM 488072
Galeopterus variegatus
USNM 317118
Plesiadapiforms
Purgatorius sp.
UCMP 197509
Plesiadapis rex
UM 94816*
Ignacius graybullianus
USNM 442235
Carpolestes simpsoni
UM 101963
Extant and extinct tarsiiforms
Teilhardina belgica
IRSNB M1235
Teilhardina brandti
USNM 540329*
Shoshonius sp.
CM 67297
Washakius sp.
UCM 64160
Hemiacodon gracilis
AMNH 12613
Omomys carteri
UM 38321
Ourayia uintenis
SDSNH 69378
Vastanomys majori
GU 800
Anemorhysis sp.
UCM 93768
Necrolemur antiquus
ISE-M-BFI-811
Tarsius syrichta
DPC 0127
Tarsius bancanus
AMNH 109367
Notharctids
Donrussellia provincialis
MNHN RI 428
Cantius ralstoni
UM 87475
Notharctus tenebrosus
AMNH 12000*
Smilodectes gracilis
AMNH 131774*

Fig. S2. (previous page)

Comparative plates of posterior trochlear shelf morphology for scandentians, dermopterans, plesiadapiforms, tarsiiforms, and notharctids. These plates provide a guideline for development of PTS; actual measurements were taken following protocols detailed in the methods section and shown in Fig. S1. Circle approximates curvature of the lateral tibial facet (i.e., the base circle of the cam); dotted lines indicate anterior and posterior extent of the base circle. Ellipses approximate tendon of the *flexor fibularis* muscle and are color-coded by species mean PTS index. Blue: PTS index < 0.75; light blue: 0.75-1.00; light red: 1.00-1.25; red: >1.25. Views for each specimen are medial (left) and dorsal (right). * indicates chirality has been reversed for consistency. Scale bars equal 3 mm.

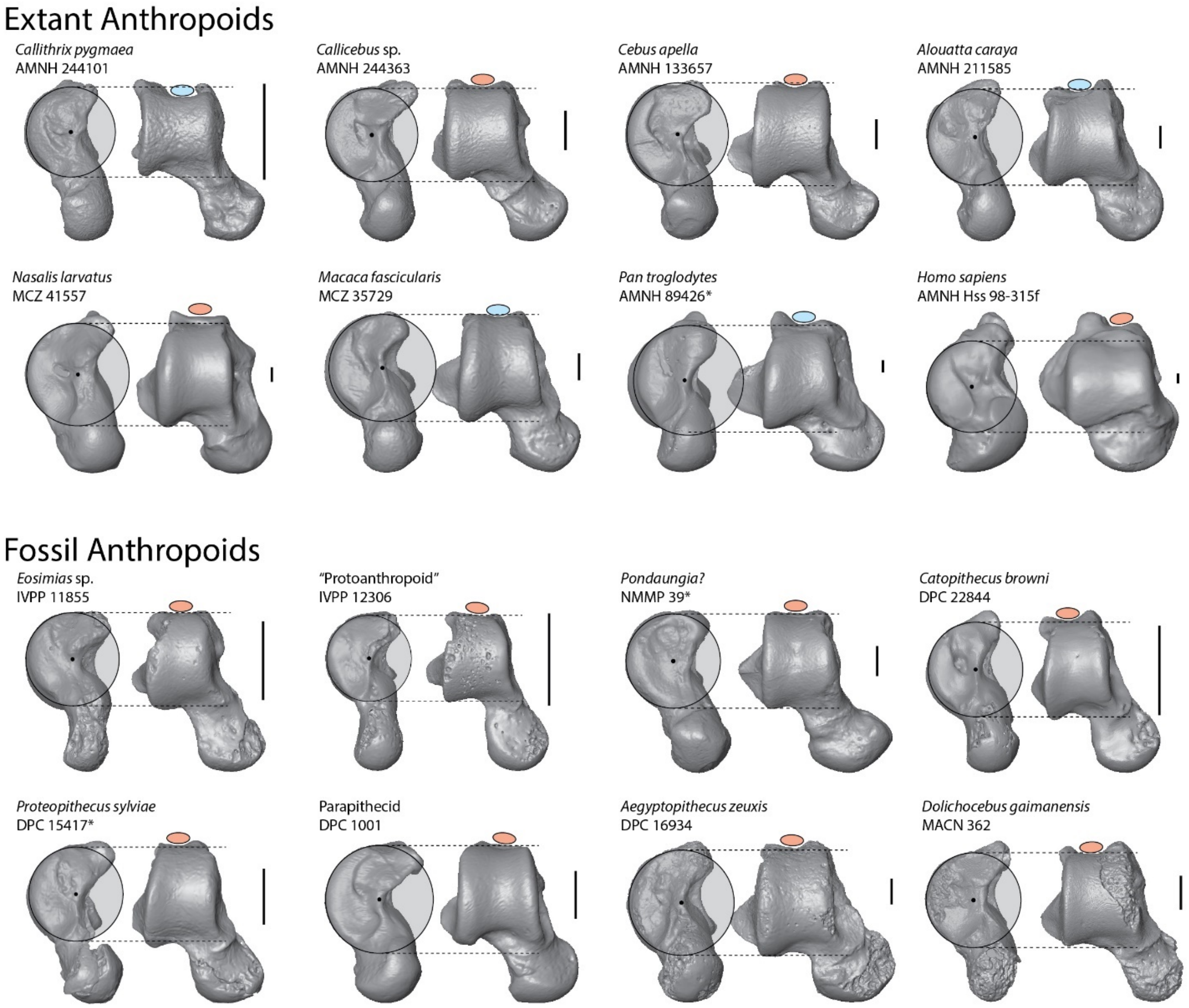

Fig. S3.

Comparative plates of posterior trochlear shelf morphology for extant and extinct anthropoids. Additional detail in caption of Fig. S2. Scale bars equal 3 mm.

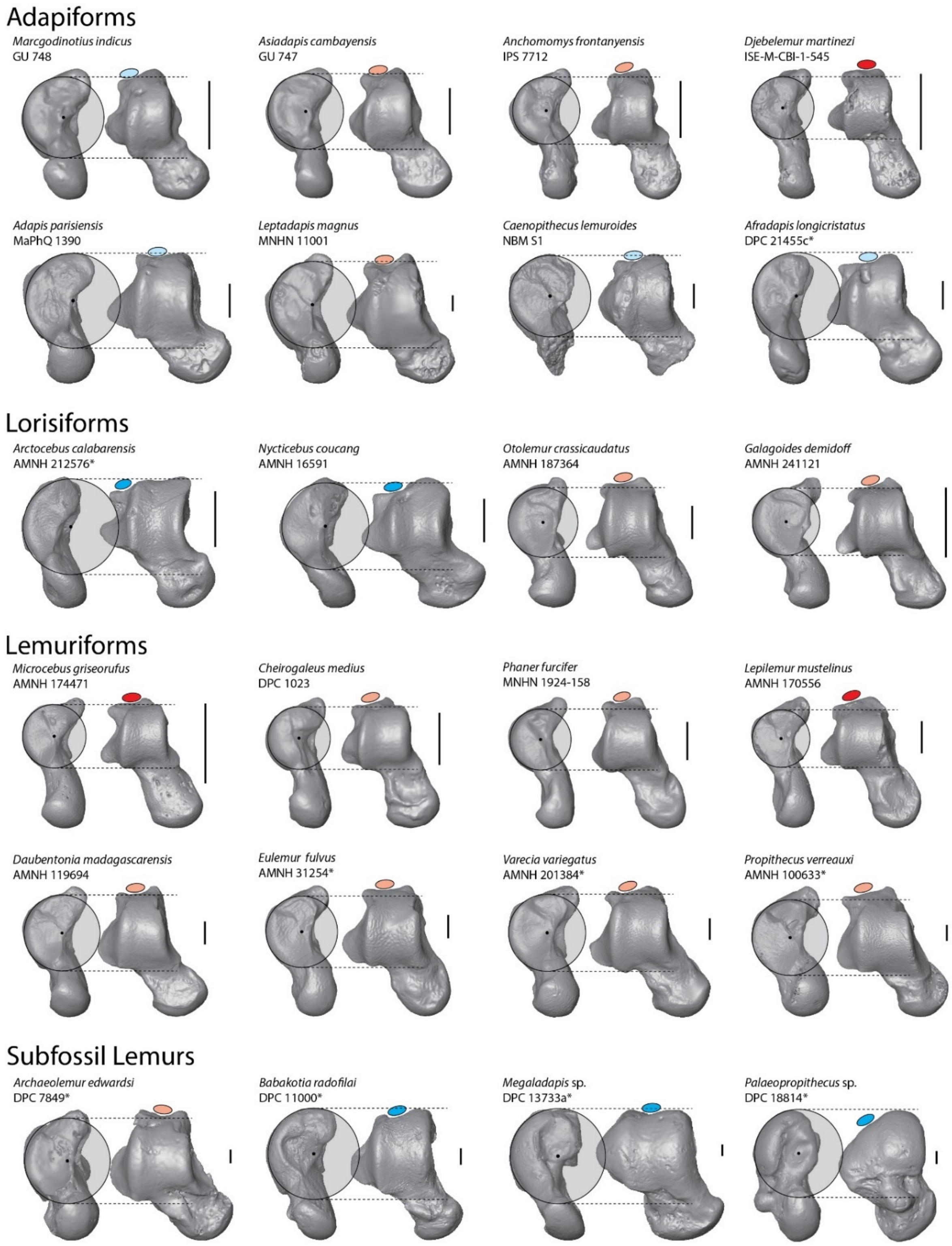

Fig. S4.

Comparative plates of posterior trochlear shelf morphology for non-notharctid adapiforms, lorisiforms, and lemuriforms. Additional detail in caption of Fig. S2. Scale bars equal 3 mm.

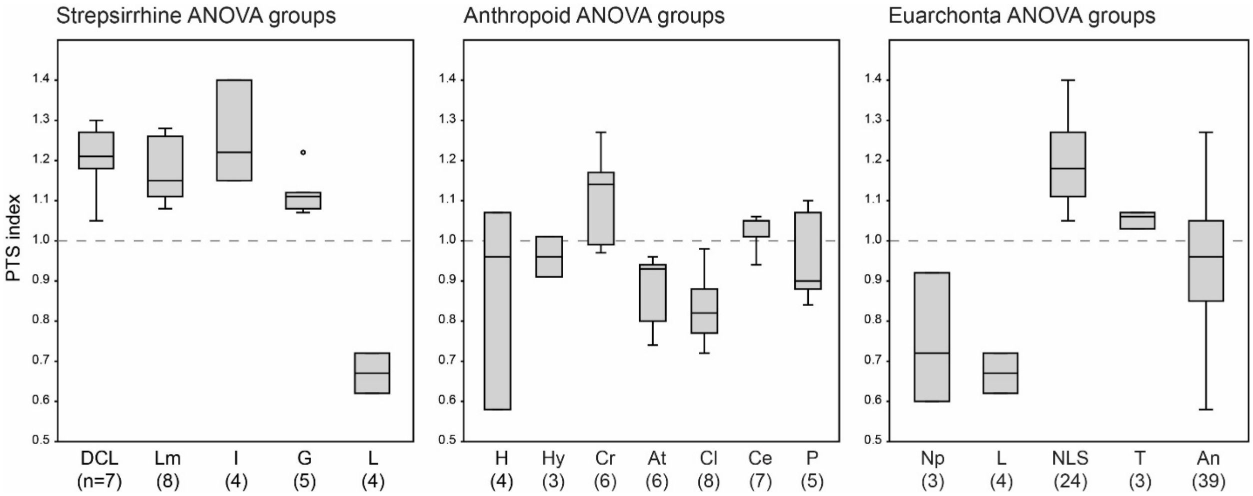

Fig. S5.

Boxplots of group means for PTS index compared with ANOVA and reported in Table S1. Number in parentheses indicates number of species in each group. Abbreviations are: DCL, *Daubentonia*+cheirogaleids+lepilemurids; Lm, lemurids; I, indriids; G, galagids; L, lorisids; H, hominids; Hy, hylobatids; Cr, cercopithecoids; At, atelids; Cl, callitrichines; Ce, cebines/aotines; P, pitheciines; Np, non-primates; NLS, non-lorisid strepsirrhines; T, tarsiers; An, anthropoids.

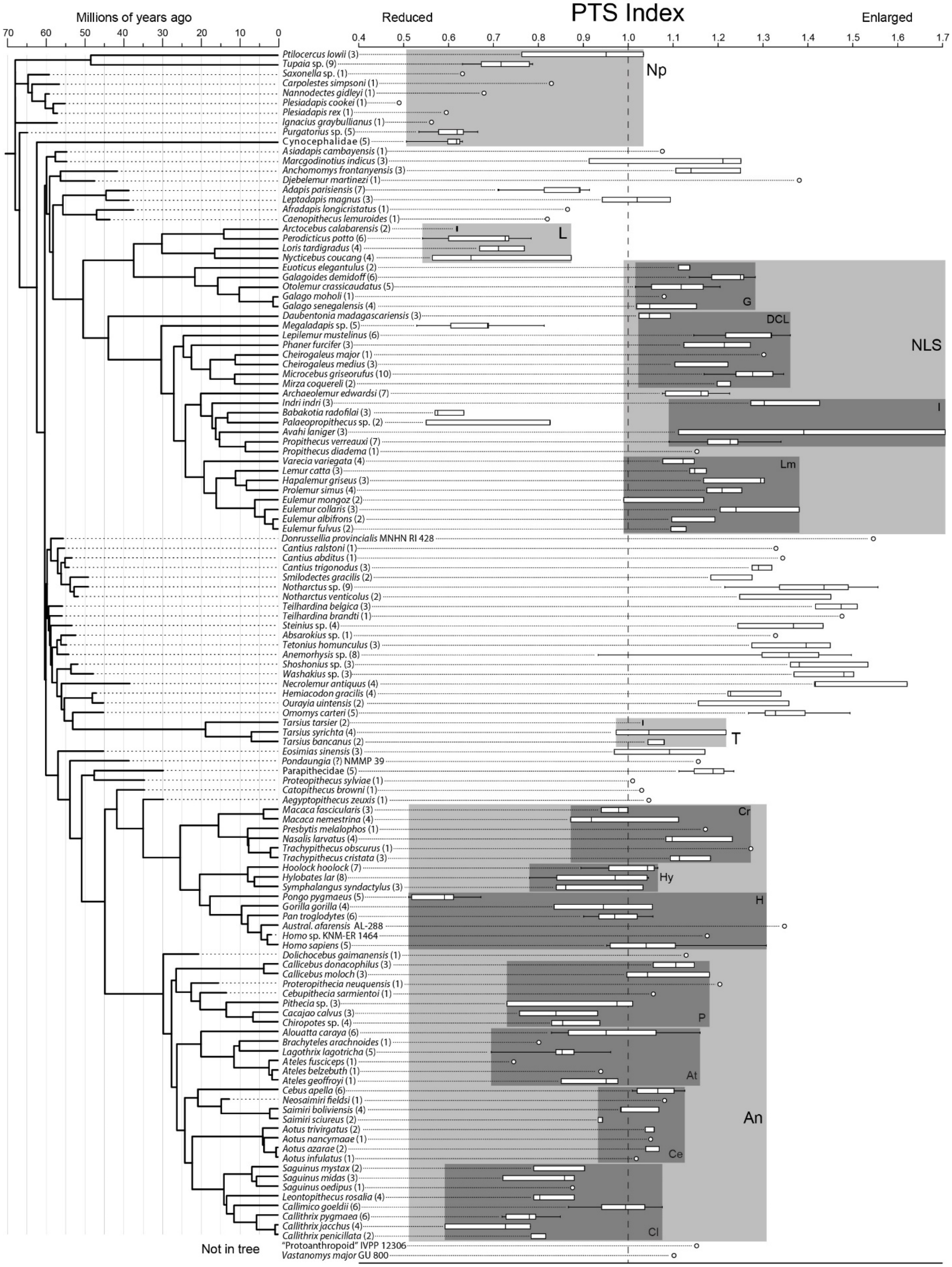

Millions of years ago
70 60 50 40 30 20 10 0
Reduced
PTS Index
Enlarged
0.4 0.5 0.6 0.7 0.8 0.9 1.0 1.1 1.2 1.3 1.4 1.5 1.6 1.7
Ptilocercus lowii (3)
Tupaia sp. (9)
Saxonella sp. (1)
Carpolestes simpsoni (1)
Nannodectes gidleyi (1)
Plesiadapis cookei (1)
Plesiadapis rex (1)
Ignacius graybullianus (1)
Purgatorius sp. (5)
Cynocephalidae (5)
Np
Asiadapis cambayensis (1)
Marcgodinotius indicus (3)
Anchomomys frontanyensis (3)
Djebelemur martinezi (1)
Adapis parisiensis (7)
Leptadapis magnus (3)
Afradapis longicristatus (1)
Caenopithecus lemuroides (1)
Arctocebus calabarensis (2)
Perodicticus potto (6)
Loris tardigradus (4)
Nycticebus coucang (4)
L
Euoticus elegantulus (2)
Galagoides demidoff (6)
Otolemur crassicaudatus (5)
Galago moholi (1)
Galago senegalensis (4)
G
Daubentonia madagascariensis (3)
Megaladapis sp. (5)
Lepilemur mustelinus (6)
Phaner furcifer (3)
Cheirogaleus major (1)
Cheirogaleus medius (3)
Microcebus griseorufus (10)
Mirza coquereli (2)
DCL
NLS
Archaeolemur edwardsi (7)
Indri indri (3)
Babakotia radofilai (3)
Palaeopropithecus sp. (2)
Avahi laniger (3)
Propithecus verreauxi (7)
Propithecus diadema (1)
I
Varecia variegata (4)
Lemur catta (3)
Hapalemur griseus (3)
Prolemur simus (4)
Eulemur mongoz (2)
Eulemur collaris (3)
Eulemur albifrons (2)
Eulemur fulvus (2)
Lm
Donrussellia provincialis MNHN RI 428
Cantius ralstoni (1)
Cantius abditus (1)
Cantius trigonodus (3)
Smilodectes gracilis (2)
Notharctus sp. (9)
Notharctus venticolus (2)
Teilhardina belgica (3)
Teilhardina brandti (1)
Steinius sp. (4)
Absarokius sp. (1)
Tetonius homunculus (3)
Anemorhysis sp. (8)
Shoshonius sp. (3)
Washakius sp. (3)
Necrolemur antiquus (4)
Hemiacodon gracilis (4)
Ourayia uintensis (2)
Omomys carteri (5)
Tarsius tarsier (2)
Tarsius syrichta (4)
Tarsius bancanus (2)
T
Eosimias sinensis (3)
Pondaungia (?) NMMP 39
Parapithecidae (5)
Proteopithecus sylviae (1)
Catopithecus browni (1)
Aegyptopithecus zeuxis (1)
Macaca fascicularis (3)
Macaca nemestrina (4)
Presbytis melalophos (1)
Nasalis larvatus (4)
Trachypithecus obscurus (1)
Trachypithecus cristata (3)
Cr
Hoolock hoolock (7)
Hylobates lar (8)
Symphalangus syndactylus (3)
Hy
Pongo pygmaeus (5)
Gorilla gorilla (4)
Pan troglodytes (6)
Austral. afarensis AL-288
Homo sp. KNM-ER 1464
Homo sapiens (5)
H
Dolichocebus gaimanensis (1)
Callicebus donacophilus (3)
Callicebus moloch (3)
Proteropithecia neuquensis (1)
Cebupithecia sarmientoi (1)
Pithecia sp. (3)
Cacajao calvus (3)
Chiropotes sp. (4)
P
Alouatta caraya (6)
Brachyteles arachnoides (1)
Lagothrix lagotricha (5)
Ateles fusciceps (1)
Ateles belzebuth (1)
Ateles geoffroyi (1)
At
Cebus apella (6)
Neosaimiri fieldsi (1)
Saimiri boliviensis (4)
Saimiri sciureus (2)
Aotus trivirgatus (2)
Aotus nancymaae (1)
Aotus azarae (2)
Aotus infulatus (1)
Ce
An
Saguinus mystax (2)
Saguinus midas (3)
Saguinus oedipus (1)
Leontopithecus rosalia (4)
Callimico goeldii (6)
Callithrix pygmaea (6)
Callithrix jacchus (4)
Callithrix penicillata (2)
Cl
Not in tree
"Protoanthropoid" IVPP 12306
Vastanomys major GU 800

Fig. S6. (previous page)

Boxplots of PTS indices with phylogenetic tree showing all included taxa. Boxes include 25-75% quartiles; horizontal lines in boxes indicate species means; whiskers extend to the farthest points <1.5 times the interquartile range. Groups of extant species used in within-strepsirrhine and within-haplorhine ANOVAs are indicated by dark gray boxes, while groups used in the within-Euarchonta ANOVAs are indicated by the light gray boxes (Fig. S5, Table S7). Abbreviations for taxonomic groups are defined in the caption of Fig. S5.

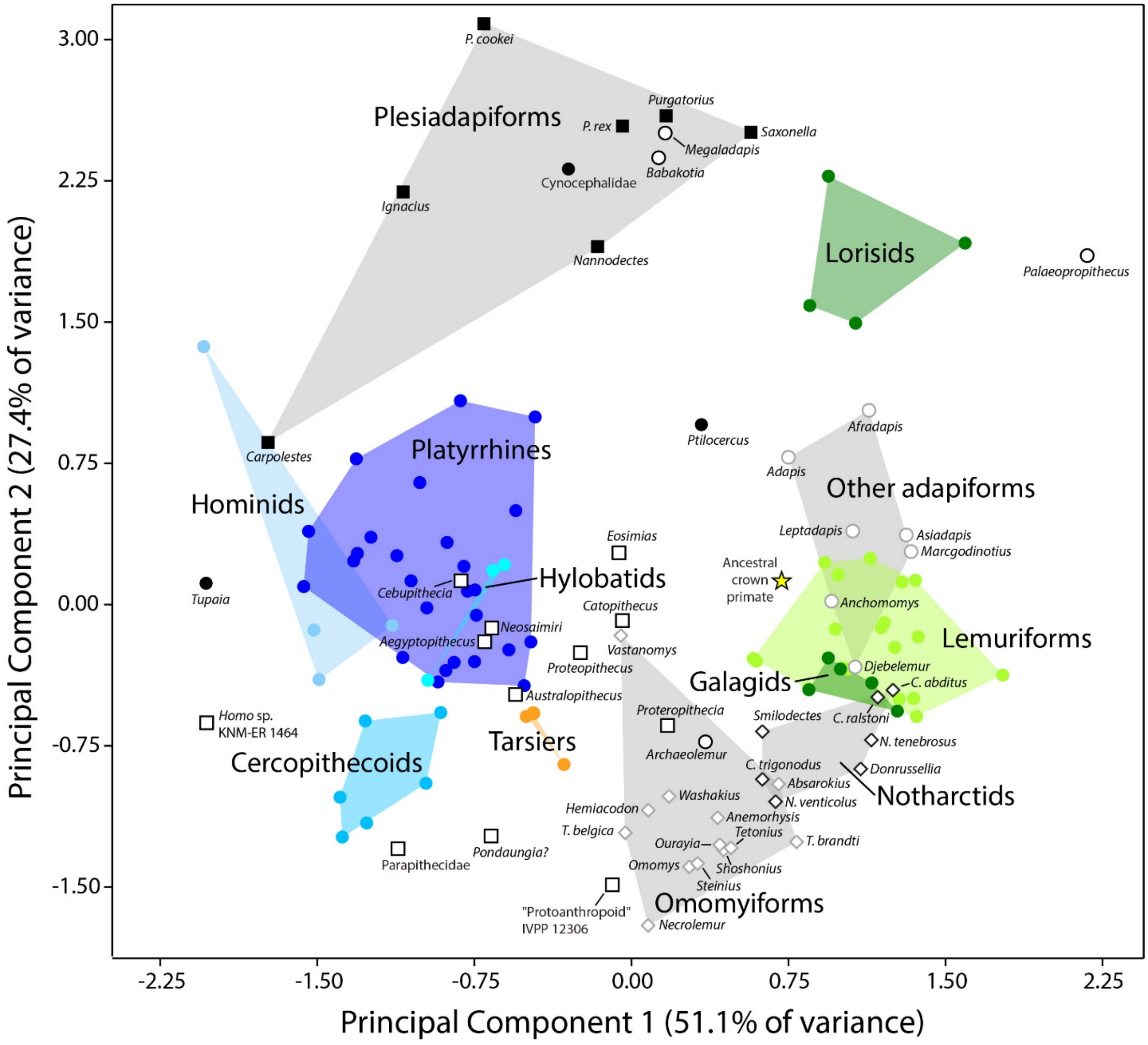

Fig. S7.

Principal components analysis of PTS index, FFG Position, fibular facet angle, and medial tibial facet morphology (the first principal component of three MTF variables in Boyer et al. [9]). Species mean values can be found in Data S2. Black circles indicate non-primate euarchontans; black open squares, fossil anthropoids; black open circles, subfossil lemurs; yellow star, mean value for ancestral crown primate node. All other groups are labeled. Extant taxa closest to ancestral crown primate include *Daubentonia madagascarensis*, *Eulemur mongoz*, and *Eulemur fulvus*.

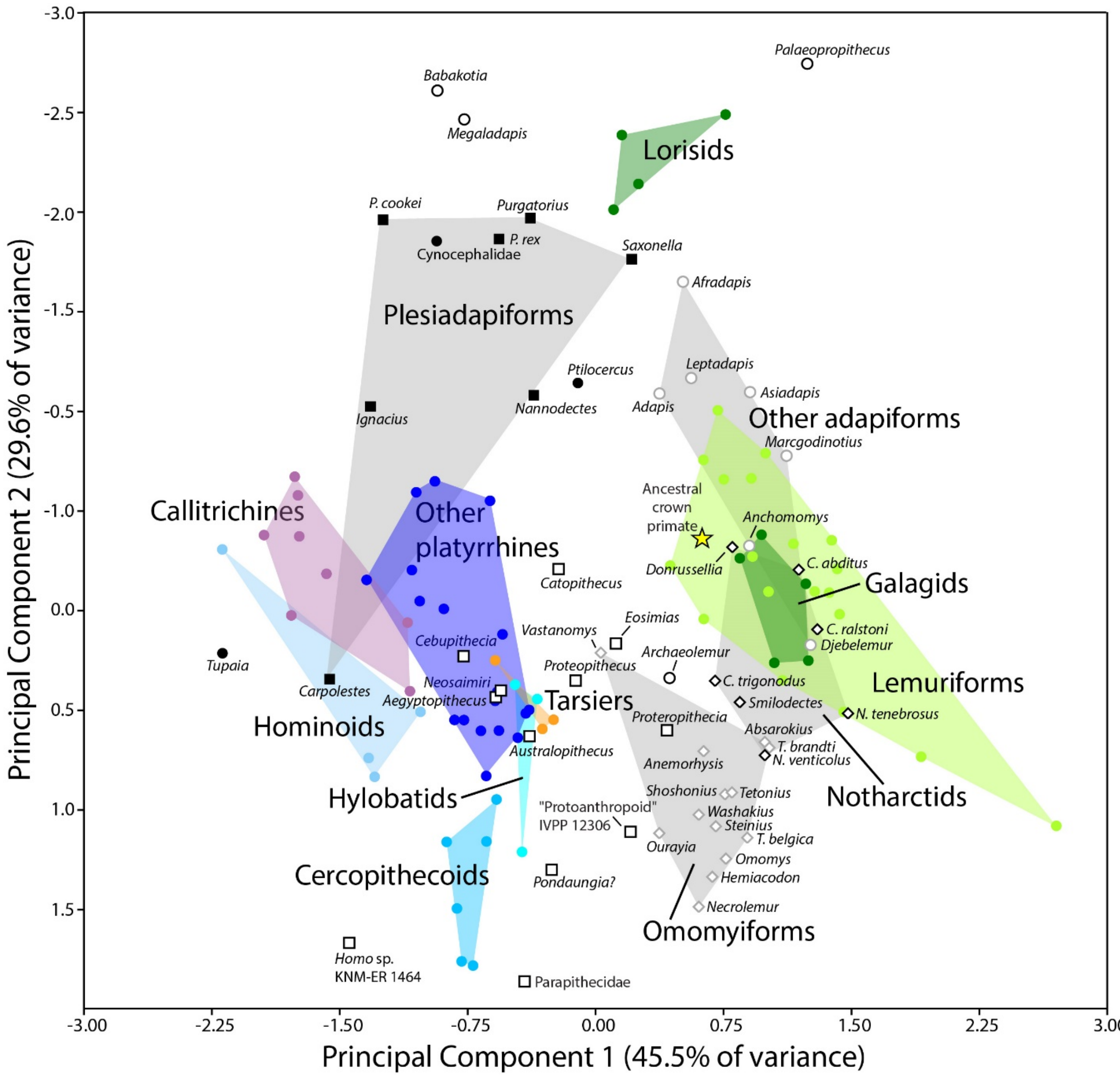

Fig. S8.

Principal components analysis of PTS index, FFG Position, FFG Ellipse, fibular facet angle, and medial tibial facet morphology (the first principal component of three MTF variables in Boyer et al. [9]). The axis for PC2 has been flipped to maintain consistency with morphospace of Fig. S7. Black circles indicate non-primate euarchontans; black open squares, fossil anthropoids; black open circles, subfossil lemurs; yellow star, mean value for crown primate ancestral node. All other groups are labeled. Extant taxa closest to ancestral crown primate include *Phaner furcifer*, *Galago senegalensis*, and *Euoticus elegantulus*.

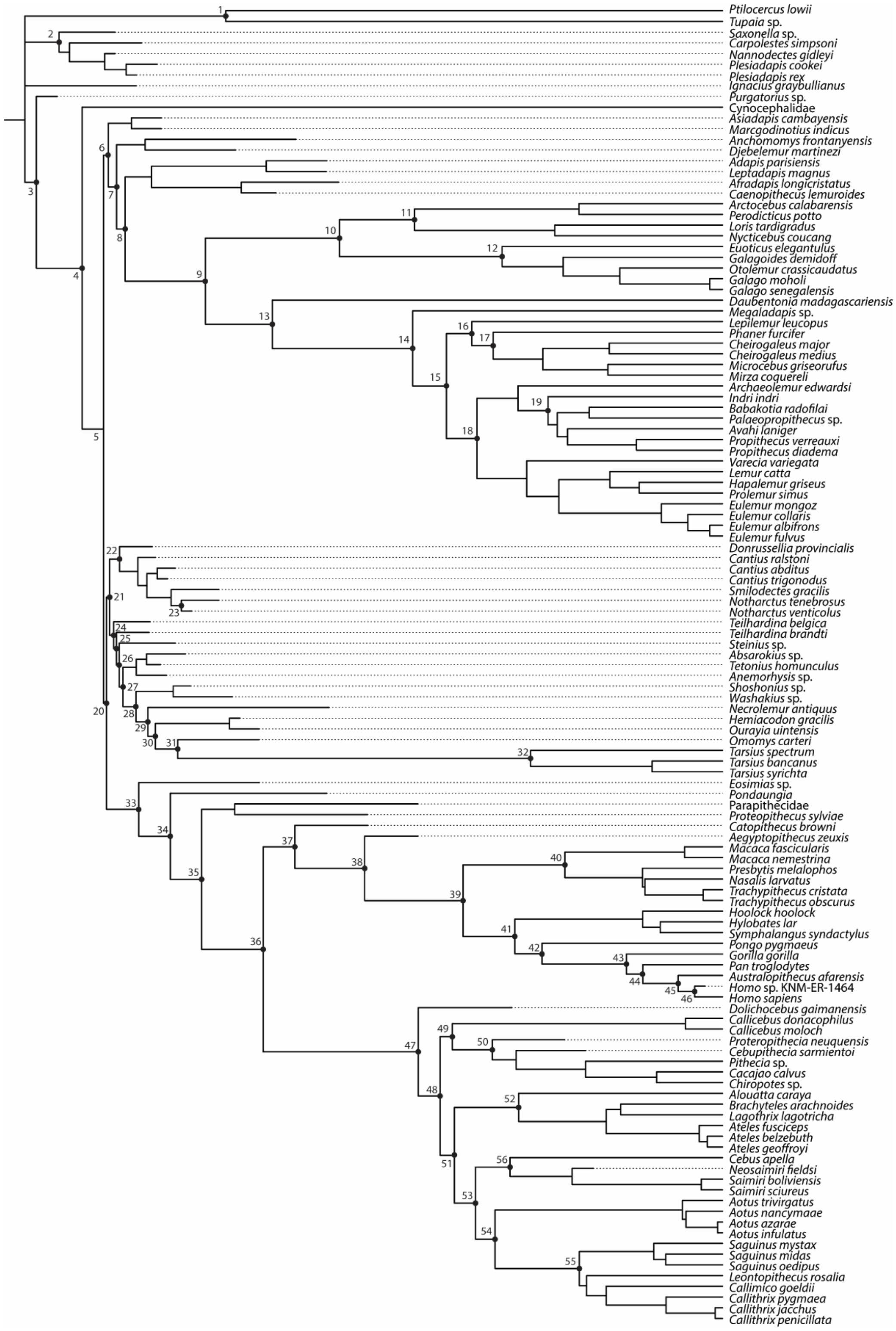

Fig. S9.

Phylogenetic tree (Tree S2) with ASR node numbers.

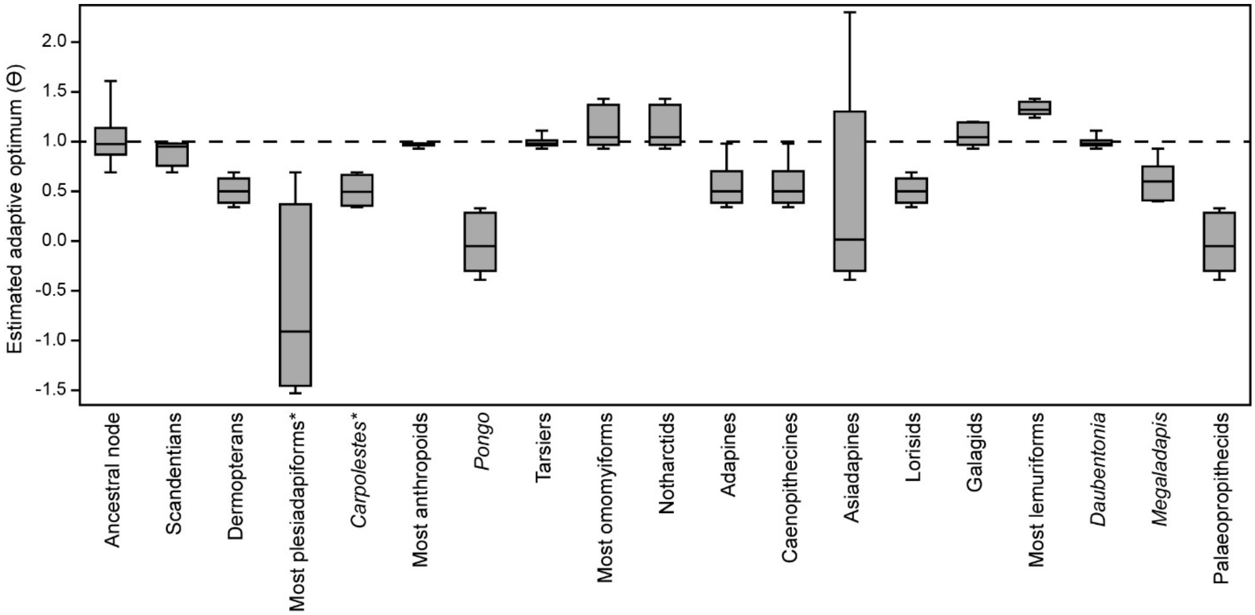

Fig. S10.
Boxplots of estimated adaptive optima (Θ) for the PTS index for select clades in six SURFACE analyses that include fossil taxa. Plesiadapiforms were excluded from the third pair of analyses, so there are only four estimated optima for taxa indicated with *.

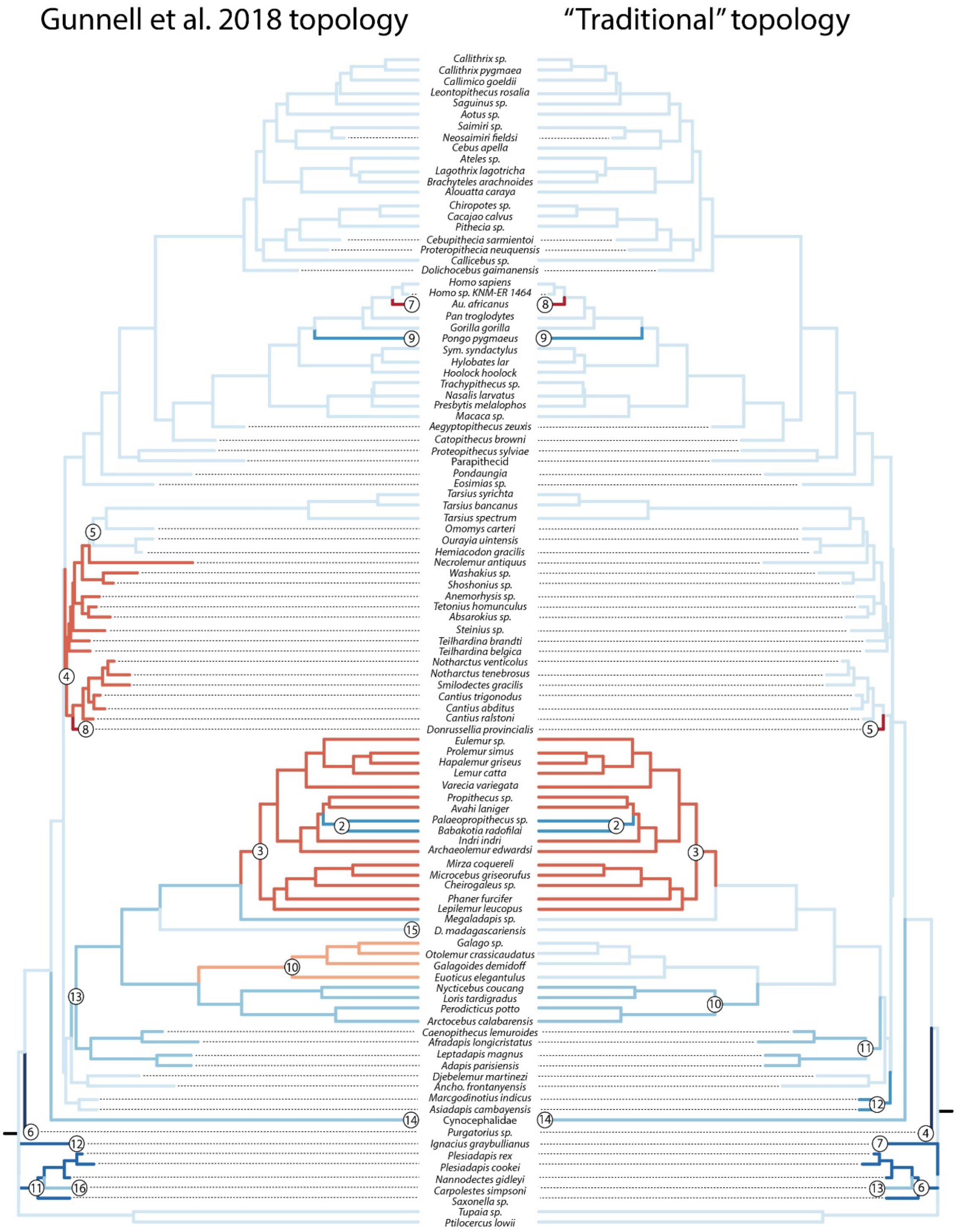

Fig. S11.

Adaptive regimes identified with SURFACE using two different topologies. All taxa included. Nodes with regime shifts are numbered and detailed in Table S18. Branch colors indicate regime shifts (red: Θ > 1.0, blue Θ < 1.0); color intensity indicates rank order of Θ values (darker colors indicate more extreme optima).

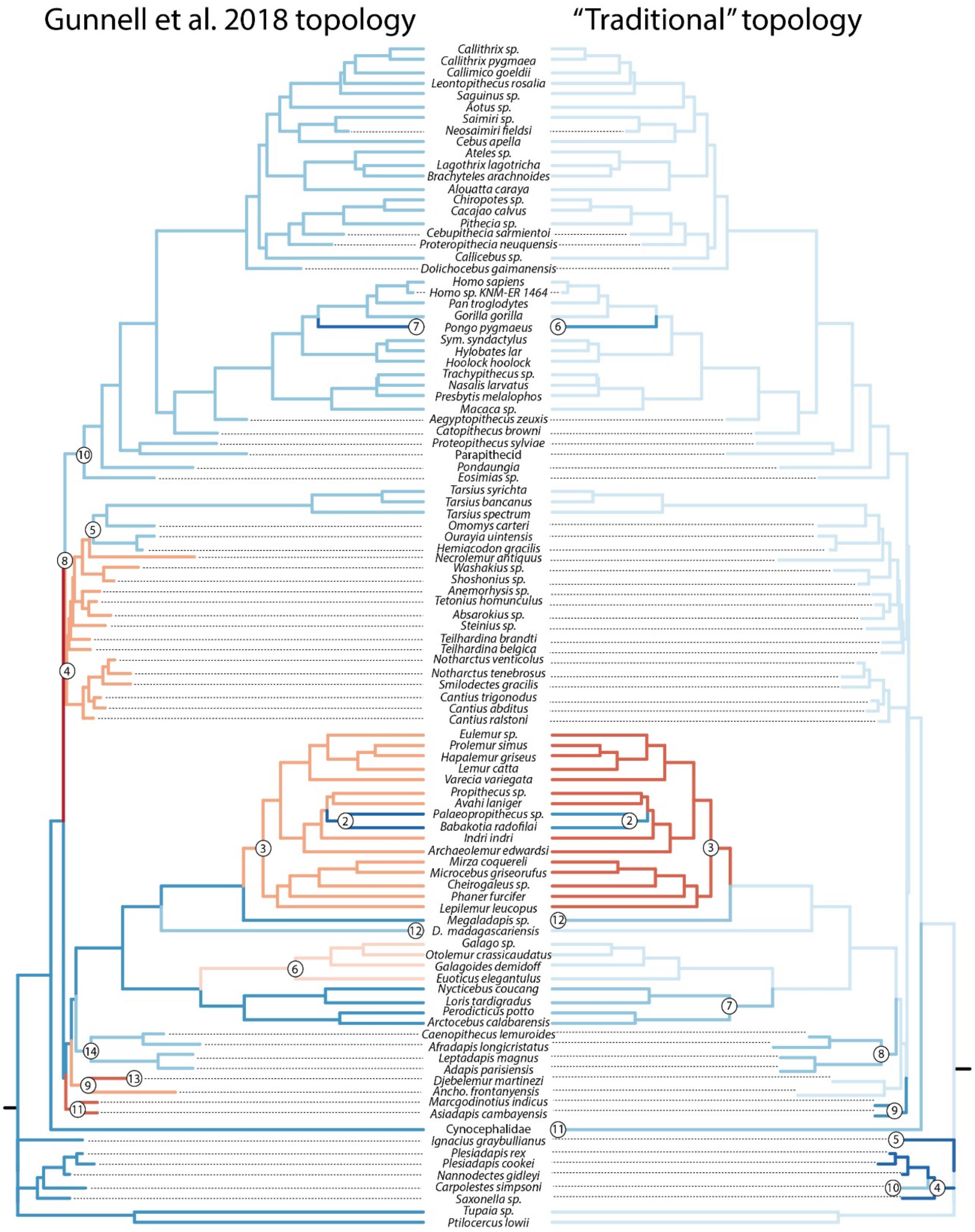

Fig. S12.

Adaptive regimes identified with SURFACE with two different topologies. Those taxa with taxon-specific regimes in Fig. S11 have been removed. Nodes with regime shifts are numbered and detailed in Table S19. Branch colors indicate regime shifts (red: $\Theta > 1.0$, blue $\Theta < 1.0$); color intensity indicates rank order of Θ values (darker colors indicate more extreme optima).

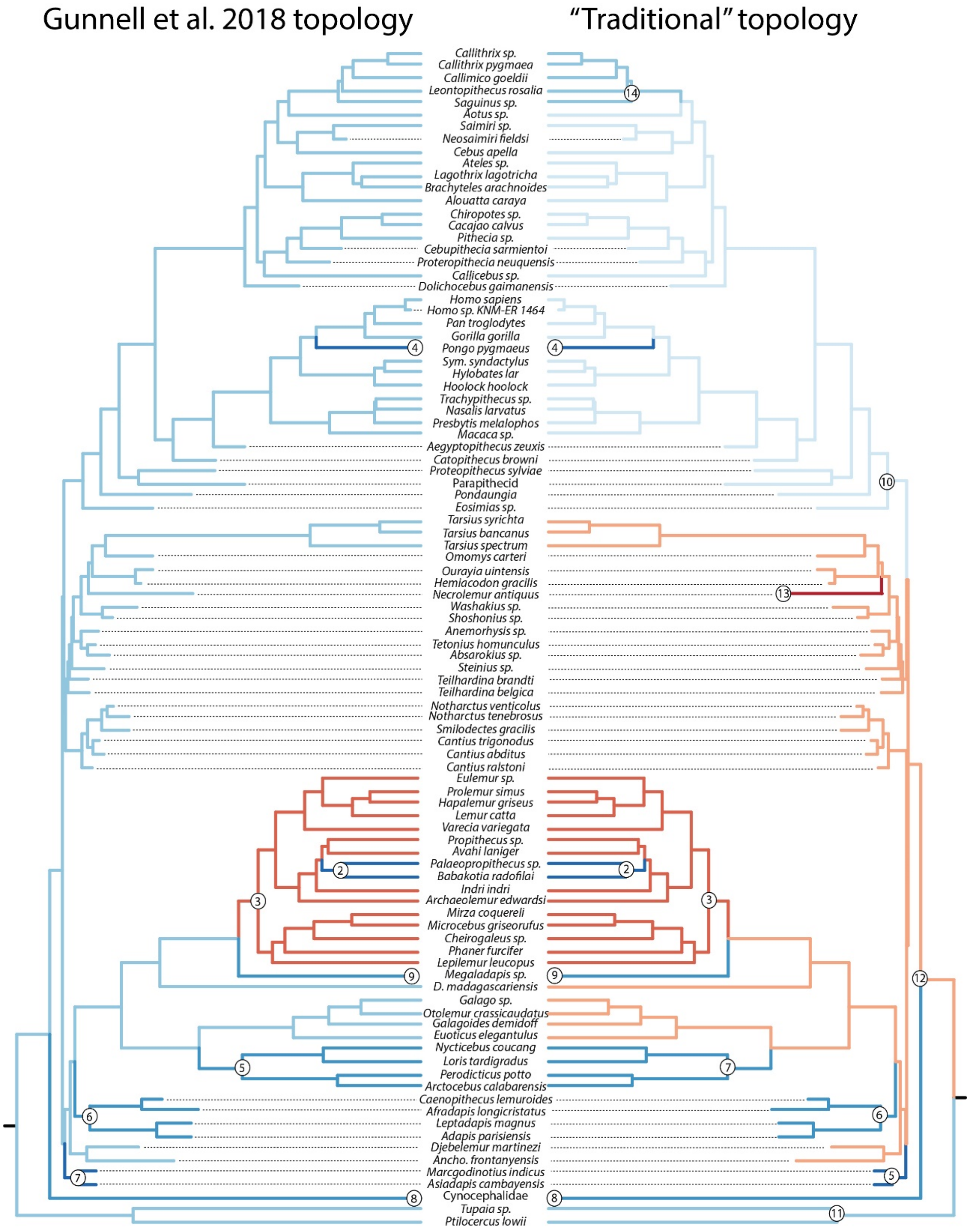

Fig. S13.

Adaptive regimes identified with SURFACE with two different topologies. Plesiadapiforms and taxa with taxon-specific regimes in Fig. S11 have been removed. Nodes with regime shifts are numbered and detailed in Table S20. Branch colors indicate regime shifts (red: ϴ > 1.0, blue ϴ < 1.0); color intensity indicates rank order of ϴ values (darker colors indicate more extreme optima).

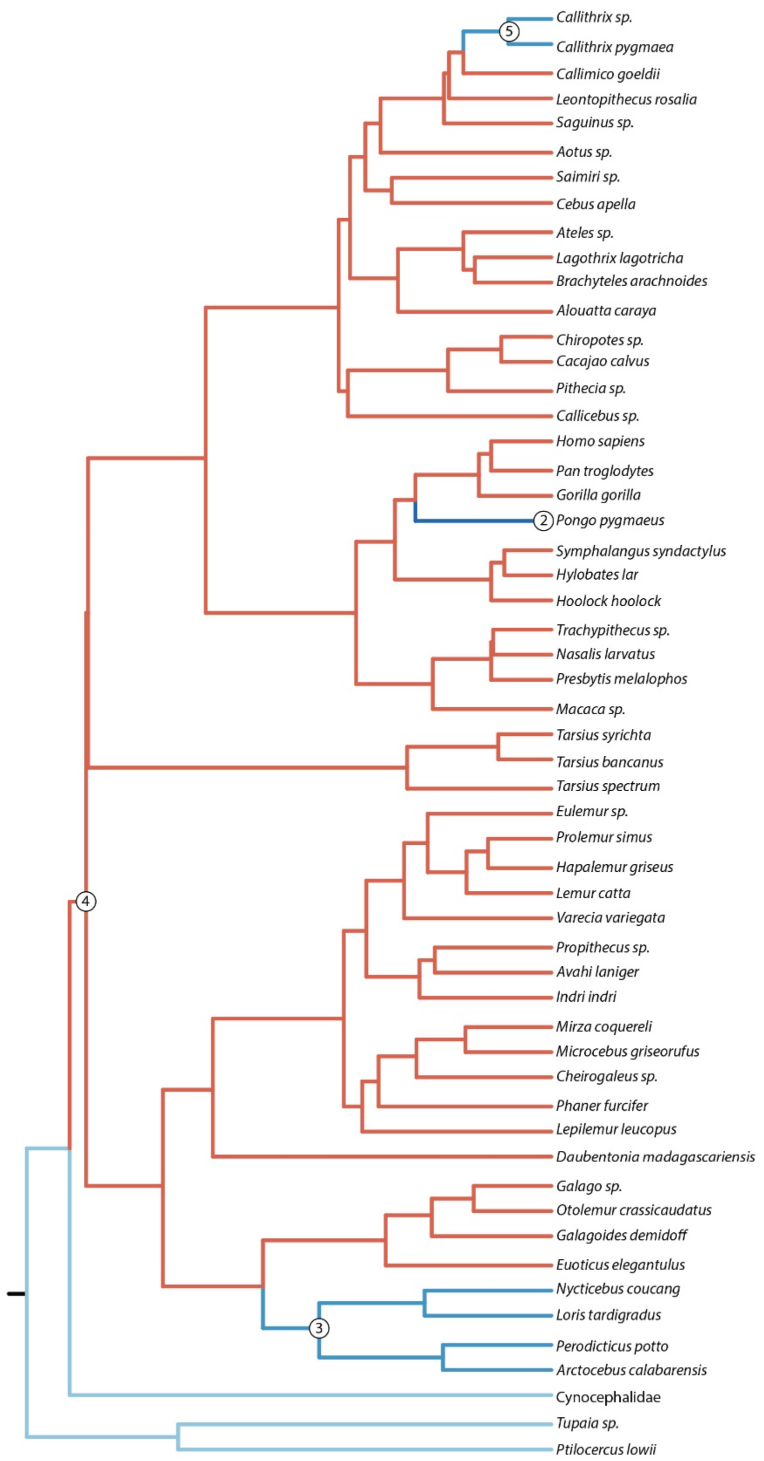

Fig. S14.

Adaptive regimes identified with SURFACE. Extant taxa only. Nodes with regime shifts are numbered and detailed in Table S21. Branch colors indicate regime shifts (red: Θ > 1.0, blue Θ < 1.0); color intensity indicates rank order of Θ values (darker colors indicate more extreme optima).

Table S1.

Mean measurements, computed indices, standard deviations (SD), and ranges of PTS measurements in extant taxa. Raw measurements for each individual specimen can be found in Data S1.

Taxon	n	Axis to Groove (mm)	SD	Range	Radius (mm)	SD	Range	PTS index	SD	Range
**Hominidae**										
Gorilla gorilla	4	20.95	4.55	14.46, 25.07	21.66	3.03	17.32, 23.79	**0.96**	0.09	0.83, 1.05
Homo sapiens	5	20.86	2.06	18.79, 23.10	19.70	2.97	14.65, 22.09	**1.07**	0.15	0.95, 1.31
Pan troglodytes	6	13.62	1.35	11.63, 15.43	13.99	1.32	12.16, 15.70	**0.97**	0.06	0.90, 1.05
Pongo pygmaeus	5	6.54	0.91	5.53, 7.82	11.26	0.73	10.29, 11.99	**0.58**	0.07	0.51, 0.67
**Hylobatidae**										
Hoolock hoolock	7	6.61	0.32	6.05, 7.09	6.53	0.34	6.08, 6.95	**1.01**	0.06	0.89, 1.07
Hylobates lar	8	5.93	1.23	3.09, 7.03	6.17	1.07	3.67, 7.22	**0.96**	0.10	0.78, 1.04
Symphalangus syndactylus	3	6.26	0.54	5.93, 6.88	6.95	1.14	5.74, 8.00	**0.91**	0.11	0.84, 1.03
**Cercopithecoidea**										
Macaca fascicularis	3	5.40	0.35	5.00, 5.66	5.55	0.19	5.32, 5.66	**0.97**	0.03	0.94, 1.00
Macaca nemestrina	4	6.35	0.87	5.10, 6.97	6.44	0.73	5.85, 7.51	**0.99**	0.11	0.87, 1.11
Nasalis larvatus	4	11.30	0.79	10.17, 12.01	9.89	0.53	9.26, 10.55	**1.14**	0.07	1.08, 1.23
Presbytis melalophos	1	7.27	-	-	6.21	-	-	**1.17**	-	-
Trachypithecus cristata	3	6.72	0.25	6.53, 7.00	5.96	0.40	5.61, 6.40	**1.13**	0.05	1.09, 1.18
Trachypithecus obscurus	1	7.75	-	-	6.09	-	-	**1.27**	-	-
**Atelidae**										
Alouatta sp.	6	6.54	0.70	5.87, 7.54	6.85	0.52	6.30, 7.74	**0.96**	0.13	0.83, 1.16
Ateles belzebuth	1	7.82	-	-	8.32	-	-	**0.94**	-	-
Ateles fusciceps	1	6.83	-	-	9.18	-	-	**0.74**	-	-
Ateles geoffroyi	4	7.65	0.91	6.53, 8.75	8.22	0.99	6.86, 8.97	**0.93**	0.06	0.85, 0.98
Brachyteles arachnoides	1	6.79	-	-	8.48	-	-	**0.80**	-	-
Lagothrix lagotricha	5	5.16	0.18	5.00, 5.45	6.17	0.77	5.67, 7.52	**0.85**	0.10	0.69, 0.96
**Callitrichinae**										
Callimico goeldii	6	2.57	0.25	2.15, 2.82	2.61	0.13	2.48, 2.84	**0.98**	0.07	0.87, 1.08
Callithrix jacchus	4	1.41	0.25	1.11, 1.62	1.96	0.15	1.78, 2.09	**0.72**	0.09	0.59, 0.78
Callithrix penicillata	2	1.50	0.07	1.45, 1.55	1.87	0.03	1.85, 1.90	**0.80**	0.02	0.78, 0.82
Callithrix pygmaea	6	1.05	0.07	0.87, 1.05	1.24	0.04	1.20, 1.32	**0.85**	0.05	0.72, 0.85
Leontopithecus rosalia	4	2.02	0.20	1.75, 2.24	2.44	0.18	2.18, 2.55	**0.83**	0.04	0.79, 0.88
Saguinus midas	3	2.08	0.12	1.95, 2.18	2.54	0.14	2.45, 2.71	**0.82**	0.09	0.72, 0.88
Saguinus mystax	2	1.72	0.10	1.66, 1.79	2.04	0.08	1.99, 2.09	**0.85**	0.08	0.79, 0.90
Saguinus oedipus	1	2.04	-	-	2.32	-	-	**0.88**	-	-
**Cebinae/Aotidae**										
Aotus azarae	2	4.29	0.05	4.25, 4.32	4.07	0.03	4.05, 4.09	**1.05**	0.02	1.04, 1.07
Aotus infulatus	1	3.34	-	-	3.28	-	-	**1.05**	-	-
Aotus nancymaae	1	3.40	-	-	3.24	-	-	**1.04**	-	-
Aotus trivirgatus	2	3.56	0.14	3.46, 3.66	3.40	0.09	3.34, 3.46	**1.05**	0.01	1.04, 1.06
Cebus apella	6	5.25	0.45	4.91, 5.85	4.97	0.38	4.36, 5.49	**1.06**	0.05	1.01, 1.13
Saimiri boliviensis	4	3.24	0.19	3.08, 3.49	3.21	0.12	3.08, 3.35	**1.01**	0.04	0.98, 1.07
Saimiri sciureus	2	3.04	0.02	3.03, 3.05	3.24	0.04	3.21, 3.27	**0.94**	0.01	0.93, 0.94
**Pitheciidae**										
Cacajao calvus	3	4.86	0.32	4.58, 5.21	5.78	0.24	5.59, 6.05	**0.84**	0.09	0.76, 0.93
Callicebus donacophilus	3	3.83	0.20	3.71, 4.05	3.47	0.10	3.36, 3.53	**1.10**	0.05	1.06, 1.15
Callicebus moloch	3	3.95	0.23	3.69, 4.13	3.69	0.28	3.41, 3.96	**1.07**	0.10	1.00, 1.18
Chiropotes sp.	4	4.54	0.50	3.92, 5.15	5.14	0.42	4.59, 5.49	**0.88**	0.05	0.83, 0.94
Pithecia sp.	3	4.16	0.32	3.95, 4.52	4.67	0.67	4.11, 5.41	**0.90**	0.15	0.73, 1.01
**Tarsiidae**										
Tarsius bancanus	2	1.79	0.03	1.77, 1.81	1.68	0.01	1.68, 1.69	**1.06**	0.03	1.04, 1.08
Tarsius syrichta	4	1.81	0.06	1.72, 1.85	1.70	0.12	1.52, 1.77	**1.07**	0.10	0.97, 1.22
Tarsius tarsier	2	1.75	0.05	1.71, 1.78	1.69	0.05	1.66, 1.73	**1.03**	0.00	-
**Cheirogaleidae**										
Cheirogaleus major	1	3.34	-	-	2.56	-	-	**1.30**	-	-
Cheirogaleus medius	3	2.12	0.13	2.01, 2.26	1.79	0.08	1.70, 1.85	**1.18**	0.07	1.10, 1.22
Microcebus griseorufus	10	1.43	0.06	1.35, 1.55	1.12	0.04	1.07, 1.17	**1.27**	0.05	1.17, 1.35
Mirza coquereli	2	2.45	0.04	2.42, 2.48	2.02	0.00	-	**1.21**	0.02	1.20, 1.23
Phaner furcifer	3	3.33	0.32	3.01, 3.65	2.77	0.26	2.48, 2.97	**1.20**	0.07	1.12, 1.27
**Lepilemuridae**										
Lepilemur mustelinus	6	3.48	0.24	3.25, 3.86	2.75	0.14	2.51, 2.93	**1.27**	0.08	1.15, 1.36
**Daubentoniidae**										
Daubentonia madagascariensis	3	5.24	0.34	5.05, 5.63	4.97	0.16	4.82, 5.14	**1.05**	0.04	1.02, 1.09

Taxon	n	Axis to Groove (mm)	SD	Range	Radius (mm)	SD	Range	PTS index	SD	Range
**Indriidae**										
Avahi laniger	3	5.61	1.42	4.72, 7.25	4.01	0.53	3.39, 4.37	**1.40**	0.30	1.11, 1.71
Indri indri	3	9.32	0.43	9.02, 9.81	6.99	0.11	6.88, 7.09	**1.33**	0.08	1.27, 1.43
Propithecus diadema	1	8.47	-	-	7.35	-	-	**1.15**	-	-
Propithecus verreauxi	7	6.27	0.42	5.39, 6.64	5.15	0.27	4.72, 5.50	**1.22**	0.08	1.09, 1.34
**Lemuridae**										
Eulemur albifrons	2	5.11	0.13	5.02, 5.21	4.48	0.38	4.21, 4.75	**1.15**	0.07	1.10, 1.19
Eulemur collaris	3	5.46	0.09	5.37, 5.54	4.30	0.36	3.89, 4.55	**1.28**	0.09	1.21, 1.38
Eulemur fulvus	2	5.19	0.28	5.00, 5.39	4.67	0.15	4.56, 4.77	**1.11**	0.02	1.10, 1.13
Eulemur mongoz	2	4.50	0.16	4.39, 4.61	4.19	0.34	3.95, 4.43	**1.08**	0.13	0.99, 1.17
Hapalemur griseus	3	4.14	0.38	3.75, 4.52	3.29	0.15	3.20, 3.47	**1.26**	0.08	1.17, 1.30
Lemur catta	3	5.11	0.17	4.92, 5.23	4.43	0.11	4.33, 4.56	**1.15**	0.02	1.14, 1.17
Prolemur simus	4	5.61	0.21	5.47, 5.91	4.60	0.22	4.36, 4.89	**1.22**	0.04	1.18, 1.25
Varecia variegata	4	6.38	0.75	5.77, 7.48	5.69	0.60	5.31, 6.58	**1.12**	0.03	1.08, 1.15
**Galagidae**										
Euoticus elegantulus	2	2.71	0.14	2.61, 2.81	2.41	0.09	2.35, 2.47	**1.12**	0.02	1.11, 1.14
Galago moholi	1	2.07	-	-	1.92	-	-	**1.08**	-	-
Galago senegalensis	4	2.08	0.06	2.00, 2.14	1.94	0.09	1.86, 2.07	**1.07**	0.06	1.02, 1.15
Galagoides demidoff	6	1.70	0.09	1.60, 1.80	1.39	0.04	1.34, 1.43	**1.22**	0.05	1.14, 1.28
Otolemur crassicaudatus	5	4.07	0.40	3.55, 4.52	3.69	0.54	3.14, 4.29	**1.11**	0.08	1.02, 1.21
**Lorisidae**										
Arctocebus calabarensis	2	1.46	0.21	1.31, 1.61	2.35	0.34	2.11, 2.60	**0.62**	0.00	-
Loris tardigradus	4	1.63	0.32	1.35, 2.05	2.25	0.40	1.90, 2.67	**0.72**	0.04	0.67, 0.77
Nycticebus coucang	4	2.16	0.21	1.91, 2.42	3.11	0.39	2.53, 3.39	**0.71**	0.13	0.56, 0.87
Perodicticus potto	6	2.41	0.26	1.95, 2.66	3.62	0.38	3.07, 4.25	**0.67**	0.09	0.54, 0.78
**Euarchonta**										
Cynocephalidae	5	2.42	0.41	1.89, 2.88	4.04	0.44	3.51, 4.66	**0.60**	0.05	0.51, 0.63
Ptilocercus lowii	3	0.94	0.06	0.88, 1.01	1.04	0.10	0.97, 1.16	**0.92**	0.14	0.76, 1.03
Tupaia sp.	9	1.03	0.09	0.90, 1.18	1.42	0.06	1.34, 1.52	**0.72**	0.05	0.63, 0.79

Table S2.

Mean measurements, computed indices, standard deviations (SD), and ranges for extinct taxa. Raw measurements for each individual specimen can be found in Data S1.

Higher taxon	Taxon	n	Axis to Groove (mm)	SD	Range	Radius (mm)	SD	Range	**PTS index**	SD	Range
**Anthropoidea: *incertae sedis* & stem**											
Eosimiidae	*Eosimias sinensis*	3	1.73	0.01	1.72, 1.75	1.62	0.17	1.48, 1.80	**1.08**	0.10	0.97, 1.17
incertae sedis	"Protoanthropoid" IVPP 12306	1	1.47	-	-	1.29	-	-	**1.14**	-	-
incertae sedis	*Pondaungia* (?) NMMP 39	1	5.29	-	-	4.57	-	-	**1.16**	-	-
Parapithecidae	Parapithecidae	5	3.74	0.36	3.41, 4.23	3.16	0.18	3.00, 3.43	**1.18**	0.05	1.11, 1.24
Parapithecidae	*Proteopithecus sylviae*	1	2.63	-	-	2.60	-	-	**1.01**	-	-
**Catarrhini**											
Hominoidea	*Australopithecus afarensis* AL-288	1	14.44	-	-	10.71	-	-	**1.35**	-	-
Hominoidea	*Homo sp.* KNM ER 1464	1	18.38	-	-	15.62	-	-	**1.18**	-	-
Oligopithecidae	*Aegyptopithecus zeuxis*	1	6.44	-	-	6.15	-	-	**1.05**	-	-
Oligopithecidae	*Catopithecus browni*	1	3.22	-	-	3.12	-	-	**1.03**	-	-
**Platyrrhini**											
incertae sedis	*Cebupithecia sarmientoi*	1	4.17	-	-	3.95	-	-	**1.06**	-	-
incertae sedis	*Dolichocebus gaimanensis*	1	4.11	-	-	3.64	-	-	**1.13**	-	-
Cebinae	*Neosaimiri fieldsi*	1	3.20	-	-	2.96	-	-	**1.08**	-	-
incertae sedis	*Proteropithecia neuquensis*	1	4.85	-	-	4.03	-	-	**1.20**	-	-
**Omomyiformes**											
Microchoerinae	*Necrolemur antiquus*	4	2.64	0.17	2.44, 2.84	1.76	0.08	1.67, 1.85	**1.50**	0.10	1.42, 1.62
Omomyidae	*Absarokius* sp.	1	1.97	-	-	1.49	-	-	**1.33**	-	-
Omomyidae	*Anemorhysis* sp.	8	1.37	0.16	1.04, 1.56	1.04	0.08	0.94, 1.15	**1.33**	0.17	0.93, 1.50
Omomyidae	*Hemiacodon gracilis*	4	2.55	0.15	2.44, 2.76	2.01	0.11	1.89, 2.15	**1.27**	0.06	1.22, 1.34
Omomyidae	*Omomys carteri*	5	2.28	0.12	2.13, 2.43	1.68	0.07	1.60, 1.75	**1.36**	0.09	1.27, 1.49
Omomyidae	*Ourayia uintensis*	2	4.52	0.70	4.03, 5.01	3.59	0.15	3.48, 3.69	**1.26**	0.14	1.16, 1.36
Omomyidae	*Shoshonius* sp.	3	1.82	0.29	1.54, 2.12	1.28	0.14	1.12, 1.38	**1.43**	0.09	1.36, 1.53
Omomyidae	*Steinius* sp.	4	1.87	0.15	1.73, 2.06	1.37	0.12	1.24, 1.51	**1.36**	0.08	1.24, 1.43
Omomyidae	*Teilhardina belgica*	3	1.35	0.01	1.34, 1.36	0.92	0.04	0.89, 0.96	**1.47**	0.05	1.42, 1.51
Omomyidae	*Teilhardina brandti*	1	1.38	-	-	0.93	-	-	**1.48**	-	-
Omomyidae	*Tetonius homunculus*	3	2.09	1.00	1.48, 3.24	1.56	0.86	1.06, 2.54	**1.37**	0.09	1.27, 1.45
Omomyidae	*Vastanomys major* GU 800	1	2.29	-	-	2.08	-	-	**1.10**	-	-
Omomyidae	*Washakius* sp.	3	1.94	0.21	1.74, 2.15	1.34	0.16	1.16, 1.45	**1.45**	0.07	1.37, 1.50
**Adapiformes**											
Notharctidae	*Cantius abditus*	1	6.55	-	-	4.88	-	-	**1.34**	-	-
Notharctidae	*Cantius ralstoni*	1	4.32	-	-	3.25	-	-	**1.33**	-	-
Notharctidae	*Cantius trigonodus*	3	5.43	0.31	5.08, 5.64	4.19	0.24	3.94, 4.42	**1.30**	0.02	1.28, 1.32
Notharctidae	*Notharctus* sp.	9	7.02	0.79	5.87, 8.45	4.98	0.50	4.47, 5.84	**1.41**	0.10	1.21, 1.56
Notharctidae	*Notharctus venticolus*	2	7.24	0.76	6.70, 7.77	5.36	0.01	5.36, 5.37	**1.35**	0.14	1.25, 1.45
Notharctidae	*Smilodectes gracilis*	2	6.33	0.02	6.31, 6.34	5.15	0.26	4.97, 5.33	**1.23**	0.07	1.18, 1.28
Notharctidae	*Donrussellia provincialis*	1	2.32	-	-	1.50	-	-	**1.55**	-	-
Adapinae	*Adapis parisiensis*	7	3.09	0.28	2.65, 3.44	3.63	0.30	3.19, 4.11	**0.85**	0.07	0.71, 0.91
Adapinae	*Leptadapis magnus*	3	8.01	1.63	6.97, 9.89	7.83	1.12	6.84, 9.05	**1.02**	0.08	0.94, 1.09
Asiadapinae	*Asiadapis cambayensis* GU 747	1	2.55	-	-	2.37	-	-	**1.08**	-	-
Asiadapinae	*Marcgodinotius indicus*	3	1.89	0.18	1.73, 2.07	1.70	0.20	1.49, 1.89	**1.12**	0.18	0.91, 1.25
Caenopithecinae	*Afradapis longicristatus*	1	3.36	-	-	3.88	-	-	**0.86**	-	-
Caenopithecinae	*Caenopithecus lemuroides*	1	3.82	-	-	4.66	-	-	**0.82**	-	-
Anchomomyini	*Anchomomys frontanyensis*	3	2.08	0.13	1.93, 2.19	1.78	0.06	1.75, 1.85	**1.17**	0.08	1.11, 1.25
Djebelemuridae	*Djebelemur martinezi*	1	1.62	-	-	1.17	-	-	**1.38**	-	-
**Lemuriformes**											
Megaladapidae	*Megaladapis* sp.	7	8.06	0.70	7.25, 9.05	12.13	1.22	10.83, 13.70	**0.67**	0.09	0.53, 0.81
Archaeolemuridae	*Archaeolemur edwardsi*	7	9.87	0.25	9.58, 10.33	8.57	0.40	8.04, 9.07	**1.15**	0.05	1.08, 1.23
Palaeopropithecidae	*Babakotia radofilai*	3	4.76	0.38	4.53, 5.20	8.02	0.16	7.91, 8.20	**0.59**	0.04	0.57, 0.63
Palaeopropithecidae	*Palaeopropithecus* sp.	2	4.72	0.52	4.36, 5.09	7.05	1.24	6.17, 7.93	**0.69**	0.20	0.55, 0.83
**Plesiadapiformes**											
Carpolestidae	*Carpolestes simpsoni*	1	1.11	-	-	1.33	-	-	**0.83**	-	-
Paromomyidae	*Ignacius graybullianus*	1	0.98	-	-	1.75	-	-	**0.56**	-	-
Plesiadapidae	*Nannodectes gidleyi*	1	1.54	-	-	2.27	-	-	**0.68**	-	-
Plesiadapidae	*Plesiadapis cookei*	1	2.67	-	-	5.46	-	-	**0.49**	-	-
Plesiadapidae	*Plesiadapis rex*	1	1.76	-	-	2.95	-	-	**0.60**	-	-
Purgatoriidae	*Purgatorius* sp.	5	0.68	0.07	0.58, 0.76	1.13	0.04	1.08, 2.37	**0.61**	0.05	0.53, 0.66
Saxonellidae	*Saxonella* sp.	1	0.68	-	-	1.07	-	-	**0.63**	-	-

Table S3.

Clade-specific phylogenetic generalized least squares (PGLS) and ordinary least squares (OLS) regressions between PTS index and ln(body mass). Regressions conducted with Tree S1.

Sample	Method	*n*	Slope	Slope 95% CI	Intercept	Intercept 95% CI	adjusted r^2	*p*	Lambda	Lambda 95% CI	Allometry
Euarchontans	PGLS	73	-0.008	(-0.040, 0.024)	0.946	(0.675, 1.216)	-0.010	0.608	0.950	(0.862, 0.987)	-
Primates	PGLS	70	-0.009	(-0.041, 0.023)	1.075	(0.799, 1.351)	-0.010	0.592	0.938	(0.828, 0.984)	-
Haplorhines	PGLS	42	-0.004	(-0.049, 0.041)	1.028	(0.644, 1.412)	-0.024	0.864	0.931	(0.720, 0.989)	-
Anthropoids	PGLS	39	-0.001	(-0.052, 0.050)	0.982	(0.514, 1.450)	-0.027	0.954	0.917	(0.675, 0.990)	-
Platyrrhines	PGLS	26	0.006	(-0.051, 0.063)	0.894	(0.471, 1.317)	-0.040	0.828	0.765	(0.374, 0.953)	-
Catarrhines	PGLS	13	-0.015	(-0.144, 0.114)	1.134	(-0.112, 2.380)	-0.084	0.802	0.985	(NA, NA)	-
Prosimians	PGLS	31	-0.015	(-0.061, 0.031)	1.124	(0.787, 1.461)	-0.020	0.525	0.934	(0.746, 0.999)	-
Strepsirrhines	PGLS	28	-0.014	(-0.064, 0.036)	1.125	(0.743, 1.507)	-0.025	0.560	0.934	(0.725, NA)	-
Lemuriforms	OLS	19	-0.015	(-0.043, 0.012)	1.317	(1.131, 1.492)	0.046	0.378	-	-	-
Lorisiforms	PGLS	9	-0.041	(-0.153, 0.071)	1.092	(0.397, 1.787)	-0.025	0.399	1.000	(0.745, NA)	-

Table S4.

PGLS regressions for extant euarchontans between component distances of the PTS index and ln(body mass). Regressions conducted with Tree S1.

Dependent	*n*	Slope	Slope 95% CI	Intercept	Intercept 95% CI	Adjusted r^2	*p*	Lambda	Lambda 95% CI	Allometry
ln(Radius)	73	0.386	(0.365, 0.407)	-1.426	(-1.583, -1.269)	0.952	**0.000**	0.660	(0.332, 0.865)	**Positive**
ln(Axis to Groove)	73	0.366	(0.320, 0.413)	-1.420	(-1.793, -1.046)	0.779	**0.000**	0.939	(0.823, 0.980)	-

Table S5.

PGLS regressions for extant and extinct euarchontans between PTS index, component distances of the PTS index, and ln(trochlear width). Regressions conducted with Tree S2.

Dependent	*n*	Slope	Slope 95% CI	Intercept	Intercept 95% CI	Adjusted r^2	*p*	Lambda	Lambda 95% CI	Allometry
PTS index	115	-0.094	(-0.172, -0.016)	0.763	(-0.749, 2.275)	0.039	**0.018**	1.000	(0.980, NA)	**Negative**
ln(Radius)	115	1.036	(0.971, 1.101)	-0.276	(-0.374, -0.178)	0.894	**0.000**	0.991	(0.897, NA)	-
ln(Axis to Groove)	115	0.948	(0.865, 1.031)	-0.615	(-0.739, -0.490)	0.814	**0.000**	0.994	(0.962, NA)	-

Table S6.
PGLS regressions for PTS index and other talar metrics. Regressions conducted with Tree S2.

Independent	n	Slope	Slope 95% CI	Intercept	Intercept 95% CI	Adjusted r2	p	Lambda	Lambda 95% CI	Allometry
FFA	115	-3.162	(-10.332, 4.008)	102.998	(95.343, 110.653)	-0.002	0.384	0.923	(0.798, 0.983)	-
MTF1	115	0.049	(-0.049, 0.147)	-0.280	(-0.385, -0.175)	0.000	0.320	0.933	(0.824, 0.981)	-
MTF2	115	-0.069	(-0.148, 0.010)	1.689	(1.606, 1.772)	0.017	0.084	0.868	(0.689, 0.951)	-
MTF3	115	-0.085	(-0.256, 0.086)	0.304	(0.115, 0.493)	0.000	0.330	0.989	(0.916, NA)	-
FFG Position	115	0.111	(-0.123, 0.345)	-0.463	(-0.725, -0.201)	-0.001	0.351	1.000	(0.986, NA)	-
FFG Ellipse	115	1.157	(0.740, 1.574)	0.221	(-0.229, 0.671)	0.202	**0.000**	0.953	(0.686, 0.994)	**Positive**

Table S7.
ANOVA and post hoc comparison tests for PTS index. Abbreviations are: DCL, *Daubentonia*+cheirogaleids+lepilemurids; Lm, lemurids; I, indriids; G, galagids; L, lorisids; H, hominids; Hy, hylobatids; Cr, cercopithecoids; At, atelids; Cl, callitrichines; Cb, cebines/aotines; P, pitheciines; Np, non-primates; NLS, non-lorisid strepsirrhines; T, tarsiers; An, anthropoids.

		Tukey's Q					
Strepsirrhines							
df (B,W)	4,23	DCL/Lm	DCL/I	DCL/G	DCL/L	Lm/I	
MSE (B,W)	0.24, 0.01	0.92	0.68	0.34	**0.00**	0.23	
F	39.78	Lm/G	Lm/L	I/G	I/L	G/L	
P(same)	**0.00**	0.82	**0.00**	**0.03**	**0.00**	**0.00**	
Anthropoids							
df (B,W)	6,32	H/Hy	H/Cr	H/At	H/Cl	H/Cb	H/P
MSE (B,W)	0.06, 0.01	0.96	**0.04**	1.00	0.96	0.45	0.96
F	5.393	Hy/Cr	Hy/At	Hy/Cl	Hy/Cb	Hy/P	Cr/At
P(same)	**0.00**	0.28	0.82	0.47	0.95	1.00	**0.02**
		Cr/Cl	Cr/Cb	Cr/P	At/Cl	At/Cb	At/P
		**0.00**	0.85	0.27	1.00	0.25	0.84
		Cl/Cb	Cl/P	Cb/P			
		0.08	0.49	0.95			
Euarchontans							
df (B,W)	4,68	Np/T	Np/A	Np/NLS	Np/L	T/A	
MSE (B,W)	0.39, 0.01	**0.00**	0.07	**0.00**	0.90	0.62	
F	27.65	T/NLS	T/L	A/NLS	A/L	NLS/L	
P(same)	**0.00**	0.37	**0.00**	**0.02**	**0.01**	**0.00**	

Table S8.
One-way t-tests that PTS index is significantly different from 1. DCL = *Daubentonia*, cheirogaleids, and lepilemurids.

Clade	n	Mean	95% CI	t	p
DCL	7	1.211	(1.134, 1.289)	6.695	**0.001**
Lemuridae	8	1.171	(1.110, 1.233)	6.601	**0.000**
Indriidae	4	1.275	(1.110, 1.452)	4.933	**0.016**
Galagidae	5	1.120	(1.046, 1.194)	4.504	**0.011**
Lorisidae	4	0.680	(0.608, 0.752)	-14.078	**0.001**
Hominidae	4	0.895	(0.552, 1.238)	-0.973	0.402
Hylobatidae	3	0.960	(0.836, 1.084)	-1.386	0.300
Cercopithecoidea	6	1.112	(0.992, 1.231)	2.409	0.061
Atelidae	6	0.870	(0.778, 0.962)	-3.615	**0.015**
Callitrichinae	8	0.831	(0.766, 0.896)	-6.156	**0.000**
Cebinae	7	1.026	(0.987, 1.065)	1.621	0.156
Pitheciidae	5	0.958	(0.811, 1.105)	-0.793	0.472
Tarsiidae	3	1.053	(1.002, 1.105)	4.438	**0.047**
Non-primate euarchontans	3	0.747	(0.345, 1.148)	-2.714	0.113

Table S9.
Summary of four variable (FFA, MTF PC1, ln(FFG Position), ln(PTS index)) principal components analysis.

PC	Eigenvalue	% variance
1	2.04587	51.147
2	1.09481	27.37
3	0.492635	12.316
4	0.36668	9.167

Table S10.
PC correlations by variable for four variable PCA.

	PC 1	PC 2	PC 3	PC 4
FFA	0.79886	0.33881	-0.3602	0.34244
MTF PC1	-0.83839	0.26007	0.21139	0.42987
ln(FFG Position)	0.77046	0.32106	0.55073	0.00268
ln(PTS index)	0.33346	-0.89961	0.12199	0.2542

Table S11.
Summary of five variable (FFA, MTF PC1, ln(FFG Position), ln(FFG Ellipse), ln(PTS index)) principal components analysis.

PC	Eigenvalue	% variance
1	2.27668	45.534
2	1.47957	29.591
3	0.493012	9.8602
4	0.459801	9.196
5	0.290933	5.8187

Table S12.
PC correlations by variable for five variable PCA.

	PC 1	PC 2	PC 3	PC 4	PC 5
FFA	0.65986	-0.55133	-0.331	0.33078	0.20402
MTF PC1	-0.82949	0.11743	0.25529	0.44014	0.19815
ln(FFG Position)	0.62075	-0.55476	0.55379	-0.0085	-0.0123
ln(FFG Ellipse)	0.6423	0.60948	0.04311	0.36914	-0.279
ln(PTS index)	0.5961	0.69468	0.09855	-0.1426	0.36338

Table S13.

Mean estimates, standard deviation (SD), variance, median, and 95% highest probability density (HPD) intervals of ancestral state reconstruction for ln(PTS index) using Tree S2 and delta model. Reconstructions based on natural log-transformed PTS index values in Tables S1 and S2. Node numbers indicated in Fig. S9.

Node	Node #	Age (mya)	Mean of Runs	Mean	SE of mean	SD	Variance	Median	95% HPD Interval
crown Scandentia	1	-48.24	-0.268	-0.269	0.001	0.177	0.031	-0.270	[-0.6136, 0.0822]
				-0.267	0.001	0.178	0.032	-0.269	[-0.6123, 0.0841]
Plesiadapiforms (- *Purgatorius*)	2	-64.42	-0.386	-0.386	0.001	0.090	0.008	-0.387	[-0.5614, -0.207]
				-0.386	0.001	0.089	0.008	-0.386	[-0.5636, -0.2126]
Purgatorius + Primatomorpha	3	-66.63	-0.338	-0.338	0.001	0.089	0.008	-0.339	[-0.5136, -0.1608]
				-0.338	0.001	0.089	0.008	-0.339	[-0.5162, -0.1646]
Primatomorpha	4	-62.19	0.019	0.020	0.001	0.093	0.009	0.019	[-0.1675, 0.1983]
				0.019	0.001	0.093	0.009	0.019	[-0.1661, 0.1982]
crown Primates	5	-60.11	0.171	0.170	0.000	0.054	0.003	0.170	[0.0598, 0.2726]
				0.171	0.000	0.054	0.003	0.171	[0.0638, 0.2745]
crown + stem Strepsirrhini	6	-59.65	0.150	0.150	0.000	0.057	0.003	0.150	[0.0366, 0.2603]
				0.150	0.000	0.057	0.003	0.150	[0.0383, 0.26]
Anchomomyini + Adapidae + crown Strepsirrhini	7	-58.85	0.129	0.129	0.000	0.066	0.004	0.129	[-0.001, 0.2589]
				0.129	0.000	0.066	0.004	0.129	[2.4406E-3, 0.2593]
Adapidae + crown Strepsirrhini	8	-58.01	0.092	0.092	0.001	0.076	0.006	0.092	[-0.0578, 0.2401]
				0.092	0.001	0.076	0.006	0.093	[-0.0585, 0.2396]
crown Strepsirrhini	9	-50.21	0.017	0.016	0.001	0.116	0.013	0.017	[-0.2091, 0.2439]
				0.017	0.001	0.115	0.013	0.017	[-0.2111, 0.236]
Lorisiformes	10	-37.19	-0.095	-0.096	0.001	0.119	0.014	-0.096	[-0.3267, 0.1397]
				-0.094	0.001	0.118	0.014	-0.093	[-0.3316, 0.1318]
Lorisidae	11	-29.91	-0.224	-0.223	0.001	0.114	0.013	-0.223	[-0.4486, -0.001]
				-0.225	0.001	0.114	0.013	-0.225	[-0.4532, -0.0066]
Galagidae	12	-21.4	0.062	0.062	0.001	0.102	0.010	0.062	[-0.1366, 0.2581]
				0.063	0.001	0.101	0.010	0.063	[-0.1386, 0.2588]
Chiromyiformes + Lemuriformes	13	-43.72	0.024	0.025	0.001	0.123	0.015	0.025	[-0.2102, 0.2716]
				0.024	0.001	0.123	0.015	0.024	[-0.2106, 0.2737]
Lemuriformes	14	-30.09	0.028	0.029	0.001	0.095	0.009	0.028	[-0.1585, 0.2102]
				0.028	0.001	0.096	0.009	0.029	[-0.1629, 0.2149]
Lemuridae	15	-19.02	0.114	0.115	0.001	0.078	0.006	0.115	[-0.0388, 0.267]
				0.114	0.001	0.077	0.006	0.114	[-0.0352, 0.27]
Lepilemur + Cheirogaleidae	16	-24.35	0.120	0.120	0.001	0.080	0.006	0.121	[-0.0428, 0.2704]
				0.119	0.001	0.080	0.006	0.119	[-0.0348, 0.2792]
Cheirogaleidae	17	-22.27	0.140	0.140	0.001	0.081	0.006	0.140	[-0.0221, 0.2925]
				0.140	0.001	0.082	0.007	0.139	[-0.0207, 0.2984]
Indrioidea + Lemuridae	18	-23.83	0.087	0.087	0.001	0.074	0.005	0.087	[-0.0605, 0.2273]
				0.087	0.001	0.074	0.006	0.087	[-0.0607, 0.232]
crown Indriidae	19	-16.95	0.046	0.045	0.000	0.067	0.004	0.045	[-0.0863, 0.1769]
				0.047	0.000	0.068	0.005	0.047	[-0.0857, 0.1802]
crown Haplorhini	20	-59.82	0.203	0.203	0.000	0.051	0.003	0.203	[0.1043, 0.3019]
				0.203	0.000	0.051	0.003	0.203	[0.1008, 0.2998]
Tarsiiforms	21	-59.51	0.243	0.243	0.000	0.047	0.002	0.243	[0.149, 0.3338]
				0.243	0.000	0.047	0.002	0.243	[0.1534, 0.3374]
Notharctidae	22	-58.55	0.289	0.289	0.000	0.054	0.003	0.288	[0.1825, 0.3924]
				0.290	0.000	0.054	0.003	0.290	[0.1883, 0.3983]
Notharctus	23	-52.55	0.297	0.297	0.000	0.043	0.002	0.296	[0.2142, 0.3804]
				0.297	0.000	0.042	0.002	0.297	[0.2157, 0.3813]
T. belgica + other tarsiiforms	24	-59.13	0.274	0.274	0.000	0.046	0.002	0.274	[0.1811, 0.3633]
				0.274	0.000	0.046	0.002	0.274	[0.1815, 0.3625]
T. brandti + other tarsiiforms	25	-58.86	0.286	0.286	0.000	0.046	0.002	0.286	[0.1944, 0.3766]
				0.286	0.000	0.046	0.002	0.286	[0.1969, 0.3773]
Steinius + other tarsiiforms	26	-58.58	0.289	0.289	0.000	0.047	0.002	0.289	[0.1975, 0.3809]
				0.289	0.000	0.048	0.002	0.289	[0.1992, 0.3869]
Anaptomorphinae + other tarsiiforms	27	-58.22	0.291	0.291	0.000	0.049	0.002	0.292	[0.1987, 0.3928]
				0.291	0.000	0.050	0.002	0.292	[0.1987, 0.3927]
Washakiini + other tarsiiforms	28	-56.94	0.298	0.297	0.000	0.061	0.004	0.298	[0.174, 0.4148]
				0.298	0.000	0.062	0.004	0.299	[0.1784, 0.419]
Microchoerinae + other tarsiiforms	29	-55.81	0.293	0.292	0.000	0.070	0.005	0.292	[0.1569, 0.4312]
				0.293	0.000	0.069	0.005	0.293	[0.1579, 0.4308]

Node	Node #	Age (mya)	Mean of Runs	Mean	SE of mean	SD	Variance	Median	95% HPD Interval
Ourayini + other tarsiiforms	30	-55.08	0.285	0.285	0.001	0.072	0.005	0.284	[0.1421, 0.4251]
				0.285	0.001	0.072	0.005	0.285	[0.1474, 0.4305]
Omomyini + other tarsiiforms	31	-52.91	0.276	0.276	0.001	0.086	0.007	0.276	[0.1065, 0.4432]
				0.275	0.001	0.086	0.007	0.275	[0.1056, 0.4452]
crown Tarsiidae	32	-18.64	0.084	0.083	0.001	0.108	0.012	0.083	[-0.1321, 0.2919]
				0.085	0.001	0.108	0.012	0.085	[-0.1241, 0.2968]
Eosimiidae + Anthropoidea	33	-56.69	0.148	0.148	0.001	0.083	0.007	0.148	[-0.0088, 0.3175]
				0.148	0.001	0.084	0.007	0.147	[-0.0229, 0.3053]
Amphipithecidae + Anthropoidea	34	-53.63	0.123	0.123	0.001	0.090	0.008	0.124	[-0.0595, 0.2954]
				0.122	0.001	0.091	0.008	0.122	[-0.0579, 0.2998]
Parapithecidae + Anthropoidea	35	-50.59	0.095	0.095	0.001	0.090	0.008	0.095	[-0.081, 0.2716]
				0.095	0.001	0.090	0.008	0.095	[-0.0812, 0.2696]
crown Anthropoidea	36	-44.6	0.059	0.060	0.001	0.091	0.008	0.060	[-0.1122, 0.2424]
				0.059	0.001	0.091	0.008	0.060	[-0.1163, 0.2381]
Catopithecus + Catarrhini	37	-41.54	0.045	0.045	0.001	0.081	0.007	0.044	[-0.1175, 0.1978]
				0.045	0.001	0.081	0.007	0.045	[-0.1114, 0.2041]
Aegyptopithecus + Catarrhini	38	-34.74	0.028	0.028	0.001	0.076	0.006	0.028	[-0.1199, 0.175]
				0.028	0.001	0.076	0.006	0.028	[-0.1215, 0.1767]
crown Catarrhini	39	-25.2	-0.018	-0.018	0.001	0.087	0.008	-0.018	[-0.1892, 0.1557]
				-0.017	0.001	0.089	0.008	-0.018	[-0.1977, 0.15]
crown Cercopithecoidea	40	-15.3	0.046	0.045	0.001	0.083	0.007	0.045	[-0.1168, 0.2088]
				0.046	0.001	0.083	0.007	0.046	[-0.1142, 0.214]
Hominoidea	41	-20.17	-0.072	-0.072	0.001	0.083	0.007	-0.072	[-0.2371, 0.0886]
				-0.072	0.001	0.082	0.007	-0.072	[-0.2331, 0.091]
Hominidae	42	-17.53	-0.106	-0.106	0.001	0.082	0.007	-0.106	[-0.2648, 0.056]
				-0.105	0.001	0.082	0.007	-0.106	[-0.2673, 0.0539]
Homininae	43	-9.33	0.012	0.012	0.000	0.063	0.004	0.012	[-0.1133, 0.1341]
				0.012	0.000	0.064	0.004	0.012	[-0.113, 0.1379]
Hominini	44	-7.74	0.045	0.045	0.000	0.058	0.003	0.044	[-0.0699, 0.1583]
				0.045	0.000	0.058	0.003	0.045	[-0.0711, 0.1573]
Hominina	45	-4.3	0.147	0.147	0.000	0.044	0.002	0.147	[0.0599, 0.2349]
				0.147	0.000	0.044	0.002	0.147	[0.0637, 0.2355]
Homo	46	-2.7	0.137	0.137	0.000	0.031	0.001	0.137	[0.0767, 0.1988]
				0.137	0.000	0.031	0.001	0.137	[0.0762, 0.1972]
Dolichocebus + Platyrrhini	47	-29.55	0.052	0.052	0.001	0.074	0.005	0.052	[-0.0897, 0.1963]
				0.052	0.001	0.074	0.005	0.053	[-0.0953, 0.1915]
crown Platyrrhini	48	-27.43	0.035	0.035	0.000	0.064	0.004	0.035	[-0.0949, 0.1579]
				0.036	0.000	0.065	0.004	0.035	[-0.0952, 0.1602]
Pitheciidae	49	-26.24	0.045	0.045	0.000	0.068	0.005	0.045	[-0.0886, 0.1775]
				0.045	0.000	0.068	0.005	0.045	[-0.0818, 0.1831]
crown Pitheciinae	50	-13.3	-0.058	-0.058	0.001	0.077	0.006	-0.058	[-0.2102, 0.0894]
				-0.058	0.001	0.078	0.006	-0.058	[-0.2107, 0.093]
Atelidae + Ceboidea	51	-26.04	0.014	0.014	0.000	0.065	0.004	0.013	[-0.1158, 0.14]
				0.014	0.000	0.066	0.004	0.014	[-0.1152, 0.1454]
crown Atelidae	52	-19.8	-0.055	-0.056	0.001	0.084	0.007	-0.056	[-0.2172, 0.1095]
				-0.054	0.001	0.085	0.007	-0.054	[-0.2196, 0.1134]
crown Ceboidea	53	-23.99	0.005	0.005	0.000	0.067	0.004	0.005	[-0.1219, 0.1387]
				0.005	0.000	0.068	0.005	0.005	[-0.1266, 0.1372]
Aotidae + Callithricidae	54	-22.09	-0.016	-0.016	0.001	0.074	0.005	-0.016	[-0.1565, 0.131]
				-0.016	0.001	0.074	0.005	-0.015	[-0.163, 0.1302]
crown Callithricidae	55	-13.9	-0.128	-0.128	0.000	0.065	0.004	-0.128	[-0.2518, 4.5847E-3]
				-0.127	0.000	0.066	0.004	-0.127	[-0.2582, 8.0388E-4]
crown Cebidae	56	-20.63	0.028	0.028	0.001	0.073	0.005	0.028	[-0.1137, 0.1723]
				0.028	0.001	0.074	0.006	0.028	[-0.1143, 0.1762]

Table S14.

Mean estimates, standard deviation (SD), variance, median, and 95% highest probability density (HPD) intervals of ancestral state reconstruction for ln(PTS index) using Tree S2 and kappa model. Reconstructions based on natural log-transformed PTS index values in Tables S1 and S2. Node numbers indicated in Fig. S9.

Node	Node #	Age (mya)	Mean of Runs	Mean	SE of mean	SD	Variance	Median	95% HPD Interval
crown Scandentia	1	-48.24	-0.300	-0.300	0.001	0.099	0.010	-0.299	[-0.4856, -0.0986]
				-0.300	0.001	0.098	0.010	-0.300	[-0.5, -0.1135]
Plesiadapiforms (- *Purgatorius*)	2	-64.42	-0.399	-0.399	0.000	0.062	0.004	-0.399	[-0.5212, -0.2788]
				-0.399	0.000	0.062	0.004	-0.399	[-0.5173, -0.2745]
Purgatorius + Primatomorpha	3	-66.63	-0.383	-0.383	0.000	0.056	0.003	-0.383	[-0.4957, -0.2756]
				-0.383	0.000	0.057	0.003	-0.383	[-0.4956, -0.2752]
Primatomorpha	4	-62.19	-0.166	-0.166	0.001	0.081	0.006	-0.165	[-0.3271, -0.0146]
				-0.167	0.001	0.081	0.006	-0.166	[-0.3229, -0.0092]
crown Primates	5	-60.11	0.056	0.056	0.000	0.065	0.004	0.058	[-0.0749, 0.1799]
				0.056	0.000	0.064	0.004	0.057	[-0.0712, 0.1796]
crown + stem Strepsirrhini	6	-59.65	0.066	0.066	0.000	0.062	0.004	0.066	[-0.0592, 0.1861]
				0.065	0.000	0.062	0.004	0.066	[-0.0568, 0.1861]
Anchomomyini + Adapidae + crown Strepsirrhini	7	-58.85	0.067	0.067	0.000	0.067	0.004	0.067	[-0.0675, 0.1936]
				0.067	0.000	0.067	0.005	0.067	[-0.0658, 0.1978]
Adapidae + crown Strepsirrhini	8	-58.01	0.015	0.015	0.001	0.075	0.006	0.016	[-0.1299, 0.1631]
				0.015	0.001	0.076	0.006	0.016	[-0.1324, 0.1625]
crown Strepsirrhini	9	-50.21	-0.027	-0.027	0.001	0.093	0.009	-0.027	[-0.2136, 0.1539]
				-0.027	0.001	0.092	0.008	-0.028	[-0.1997, 0.1612]
Lorisiformes	10	-37.19	-0.091	-0.091	0.001	0.098	0.010	-0.091	[-0.2902, 0.0945]
				-0.091	0.001	0.098	0.010	-0.091	[-0.283, 0.1008]
Lorisidae	11	-29.91	-0.239	-0.239	0.001	0.097	0.009	-0.240	[-0.4298, -0.0495]
				-0.239	0.001	0.097	0.009	-0.239	[-0.4275, -0.0468]
Galagidae	12	-21.4	0.051	0.051	0.001	0.090	0.008	0.052	[-0.1281, 0.2257]
				0.050	0.001	0.090	0.008	0.050	[-0.135, 0.2206]
Chiromyiformes + Lemuriformes	13	-43.72	-0.021	-0.020	0.001	0.096	0.009	-0.021	[-0.2032, 0.1738]
				-0.021	0.001	0.098	0.010	-0.022	[-0.2215, 0.1604]
Lemuriformes	14	-30.09	-0.055	-0.055	0.001	0.091	0.008	-0.054	[-0.2278, 0.1303]
				-0.055	0.001	0.091	0.008	-0.056	[-0.2343, 0.1233]
Lemuridae	15	-19.02	0.109	0.109	0.001	0.082	0.007	0.108	[-0.052, 0.2709]
				0.109	0.001	0.081	0.007	0.109	[-0.0455, 0.274]
Lepilemur + Cheirogaleidae	16	-24.35	0.124	0.124	0.001	0.080	0.006	0.125	[-0.037, 0.2779]
				0.123	0.001	0.081	0.007	0.124	[-0.0331, 0.2853]
Cheirogaleidae	17	-22.27	0.154	0.154	0.001	0.082	0.007	0.153	[-0.0097, 0.3111]
				0.154	0.001	0.082	0.007	0.154	[-0.0075, 0.3102]
Indrioidea + Lemuridae	18	-23.83	0.082	0.083	0.001	0.081	0.006	0.083	[-0.0756, 0.2391]
				0.082	0.001	0.080	0.006	0.082	[-0.0807, 0.2342]
crown Indriidae	19	-16.95	0.081	0.080	0.001	0.075	0.006	0.080	[-0.0612, 0.2315]
				0.081	0.001	0.075	0.006	0.081	[-0.065, 0.2274]
crown Haplorhini	20	-59.82	0.139	0.139	0.000	0.058	0.003	0.140	[0.0253, 0.2535]
				0.138	0.000	0.059	0.003	0.139	[0.0198, 0.2516]
Tarsiiforms	21	-59.51	0.232	0.232	0.000	0.054	0.003	0.232	[0.1226, 0.3342]
				0.231	0.000	0.054	0.003	0.232	[0.1271, 0.3406]
Notharctidae	22	-58.55	0.300	0.300	0.000	0.057	0.003	0.300	[0.1899, 0.4125]
				0.300	0.000	0.057	0.003	0.300	[0.1878, 0.4113]
Notharctus	23	-52.55	0.298	0.298	0.000	0.051	0.003	0.298	[0.1969, 0.3975]
				0.298	0.000	0.051	0.003	0.298	[0.194, 0.3928]
T. belgica + other tarsiiforms	24	-59.13	0.289	0.290	0.000	0.052	0.003	0.289	[0.1888, 0.392]
				0.289	0.000	0.052	0.003	0.288	[0.1837, 0.388]
T. brandti + other tarsiiforms	25	-58.86	0.311	0.311	0.000	0.052	0.003	0.311	[0.2108, 0.4143]
				0.311	0.000	0.052	0.003	0.310	[0.2088, 0.4128]
Steinius + other tarsiiforms	26	-58.58	0.309	0.309	0.000	0.054	0.003	0.308	[0.2029, 0.4139]
				0.309	0.000	0.054	0.003	0.309	[0.2021, 0.4163]
Anaptomorphinae + other tarsiiforms	27	-58.22	0.306	0.305	0.000	0.057	0.003	0.305	[0.1981, 0.4205]
				0.306	0.000	0.058	0.003	0.306	[0.1954, 0.4223]
Washakiini + other tarsiiforms	28	-56.94	0.310	0.310	0.000	0.064	0.004	0.310	[0.1839, 0.4325]
				0.311	0.000	0.064	0.004	0.310	[0.1876, 0.4388]
Microchoerinae + other tarsiiforms	29	-55.81	0.306	0.306	0.000	0.068	0.005	0.306	[0.1719, 0.4386]
				0.305	0.000	0.068	0.005	0.306	[0.1681, 0.4321]

Node	Node #	Age (mya)	Mean of Runs	Mean	SE of mean	SD	Variance	Median	95% HPD Interval
Ourayini + other tarsiiforms	30	-55.08	0.281	0.281	0.000	0.070	0.005	0.281	[0.1446, 0.4199]
				0.281	0.001	0.070	0.005	0.281	[0.1431, 0.4205]
Omomyini + other tarsiiforms	31	-52.91	0.260	0.260	0.001	0.077	0.006	0.261	[0.1148, 0.4142]
				0.260	0.001	0.077	0.006	0.260	[0.1074, 0.4098]
crown Tarsiidae	32	-18.64	0.107	0.107	0.001	0.092	0.008	0.107	[-0.0788, 0.2827]
				0.107	0.001	0.091	0.008	0.108	[-0.0707, 0.2853]
Eosimiidae + Anthropoidea	33	-56.69	0.111	0.111	0.001	0.072	0.005	0.112	[-0.0271, 0.2541]
				0.111	0.001	0.071	0.005	0.111	[-0.025, 0.254]
Amphipithecidae + Anthropoidea	34	-53.63	0.107	0.107	0.001	0.076	0.006	0.106	[-0.0361, 0.2609]
				0.107	0.001	0.077	0.006	0.107	[-0.0403, 0.2622]
Parapithecidae + Anthropoidea	35	-50.59	0.087	0.087	0.001	0.080	0.006	0.088	[-0.074, 0.2416]
				0.086	0.001	0.079	0.006	0.087	[-0.0727, 0.2396]
crown Anthropoidea	36	-44.6	0.061	0.060	0.001	0.083	0.007	0.060	[-0.1046, 0.2216]
				0.061	0.001	0.082	0.007	0.062	[-0.0998, 0.2229]
Catopithecus + Catarrhini	37	-41.54	0.041	0.041	0.001	0.076	0.006	0.041	[-0.1025, 0.1981]
				0.041	0.001	0.075	0.006	0.041	[-0.1076, 0.1866]
Aegyptopithecus + Catarrhini	38	-34.74	0.023	0.023	0.001	0.075	0.006	0.023	[-0.1236, 0.1731]
				0.023	0.001	0.076	0.006	0.022	[-0.1208, 0.176]
crown Catarrhini	39	-25.2	-0.023	-0.023	0.001	0.087	0.008	-0.022	[-0.1942, 0.1454]
				-0.023	0.001	0.088	0.008	-0.023	[-0.2002, 0.1442]
crown Cercopithecoidea	40	-15.3	0.034	0.033	0.001	0.085	0.007	0.032	[-0.1242, 0.2095]
				0.034	0.001	0.086	0.007	0.035	[-0.1319, 0.2044]
Hominoidea	41	-20.17	-0.097	-0.097	0.001	0.085	0.007	-0.097	[-0.2595, 0.0726]
				-0.097	0.001	0.084	0.007	-0.096	[-0.2544, 0.0745]
Hominidae	42	-17.53	-0.174	-0.174	0.001	0.084	0.007	-0.175	[-0.3387, -0.011]
				-0.175	0.001	0.084	0.007	-0.175	[-0.3312, -0.0022]
Homininae	43	-9.33	-0.043	-0.043	0.001	0.075	0.006	-0.043	[-0.1931, 0.0982]
				-0.044	0.001	0.074	0.005	-0.044	[-0.192, 0.0983]
Hominini	44	-7.74	0.019	0.019	0.000	0.070	0.005	0.019	[-0.1204, 0.157]
				0.019	0.000	0.070	0.005	0.019	[-0.1145, 0.1619]
Hominina	45	-4.3	0.142	0.143	0.000	0.062	0.004	0.142	[0.0187, 0.262]
				0.142	0.000	0.062	0.004	0.142	[0.0199, 0.2631]
Homo	46	-2.7	0.128	0.129	0.000	0.051	0.003	0.129	[0.0296, 0.2277]
				0.128	0.000	0.050	0.003	0.128	[0.0269, 0.2239]
Dolichocebus + Platyrrhini	47	-29.55	0.068	0.068	0.001	0.077	0.006	0.068	[-0.0783, 0.2235]
				0.068	0.001	0.077	0.006	0.068	[-0.0855, 0.2151]
crown Platyrrhini	48	-27.43	0.044	0.044	0.001	0.074	0.005	0.044	[-0.0937, 0.1943]
				0.045	0.001	0.074	0.005	0.045	[-0.0976, 0.1916]
Pitheciidae	49	-26.24	0.062	0.062	0.001	0.077	0.006	0.062	[-0.0907, 0.2083]
				0.062	0.001	0.077	0.006	0.061	[-0.0853, 0.2189]
crown Pitheciinae	50	-13.3	-0.061	-0.061	0.001	0.080	0.006	-0.062	[-0.2213, 0.0943]
				-0.061	0.001	0.079	0.006	-0.061	[-0.2132, 0.0973]
Atelidae + Ceboidea	51	-26.04	0.008	0.008	0.001	0.076	0.006	0.008	[-0.1406, 0.1573]
				0.007	0.001	0.077	0.006	0.008	[-0.1484, 0.1511]
crown Atelidae	52	-19.8	-0.059	-0.059	0.001	0.084	0.007	-0.059	[-0.228, 0.1018]
				-0.059	0.001	0.084	0.007	-0.059	[-0.2201, 0.108]
crown Ceboidea	53	-23.99	0.002	0.002	0.001	0.077	0.006	0.002	[-0.1468, 0.157]
				0.001	0.001	0.077	0.006	0.002	[-0.1506, 0.1509]
Aotidae + Callithricidae	54	-22.09	-0.024	-0.024	0.001	0.081	0.007	-0.024	[-0.1781, 0.1382]
				-0.023	0.001	0.082	0.007	-0.024	[-0.1838, 0.1396]
crown Callithricidae	55	-13.9	-0.115	-0.115	0.001	0.077	0.006	-0.115	[-0.265, 0.0356]
				-0.115	0.001	0.076	0.006	-0.115	[-0.2629, 0.0343]
crown Cebidae	56	-20.63	0.028	0.028	0.001	0.079	0.006	0.029	[-0.1232, 0.1881]
				0.029	0.001	0.079	0.006	0.028	[-0.1304, 0.1792]

Table S15.

Estimated marginal likelihoods for different evolutionary models and scaling parameters using Tree S2. Bold text indicates model and scaling parameter with highest likelihood.

Scaling parameter	Directional model Harmonic mean	Random walk Harmonic mean
none	26.068	35.757
delta	**36.000**	**40.691**
kappa	30.436	39.469
lambda	21.453	30.818

Table S16.

Contrasts of mean ln(PTS index) ancestral state reconstruction values recovered with Tree S2 and Tree S3. Mean ASR values derived from two separate runs with a delta model. Nodes with >0.01 difference are in bold. Compared to the results presented in the manuscript, the alternative tree generates a higher PTS index at the base of crown primates.

Node	Mean lnPTS index (Tree S2)	Mean lnPTS index (Tree S3)	Contrast of models
crown Scandentia	-0.268	-0.267	-0.001
Plesiadapiforms (- *Purgatorius*)	-0.386	-0.386	-0.001
Purgatorius + Primatomorpha	-0.338	-0.332	-0.006
**Primatomorpha**	**0.019**	**0.035**	**-0.016**
**crown Primates**	**0.171**	**0.191**	**-0.020**
**crown + stem Strepsirrhini**	**0.150**	**0.177**	**-0.027**
**Anchomomyini + Adapidae + crown Strepsirrhini**	**0.129**	**0.149**	**-0.021**
**Adapidae + crown Strepsirrhini**	**0.092**	**0.109**	**-0.017**
crown Strepsirrhini	0.017	0.026	-0.009
Lorisiformes	-0.095	-0.092	-0.003
Lorisidae	-0.224	-0.223	-0.001
Galagidae	0.062	0.063	-0.001
Chiromyiformes + Lemuriformes	0.024	0.030	-0.006
Lemuriformes	0.028	0.030	-0.001
Lemuridae	0.114	0.116	-0.001
Lepilemur + Cheirogaleidae	0.120	0.121	-0.001
Cheirogaleidae	0.140	0.141	-0.001
Indrioidea + Lemuridae	0.087	0.089	-0.002
crown Indriidae	0.046	0.046	0.000
crown Haplorhini	0.203	0.211	-0.007
**Notharctidae**	**0.289**	**0.273**	**0.016**
Notharctus	0.297	0.296	0.001
T. belgica + other tarsiiforms	0.274	0.270	0.004
T. brandti + other tarsiiforms	0.286	0.283	0.003
Steinius + other tarsiiforms	0.289	0.286	0.003
Anaptomorphinae + other tarsiiforms	0.291	0.289	0.002
Washakiini + other tarsiiforms	0.298	0.297	0.001
Microchoerinae + other tarsiiforms	0.293	0.292	0.001
Ourayini + other tarsiiforms	0.285	0.283	0.001
Omomyini + other tarsiiforms	0.276	0.275	0.001
crown Tarsiidae	0.084	0.083	0.001
Eosimiidae + Anthropoidea	0.148	0.150	-0.003
Amphipithecidae + Anthropoidea	0.123	0.124	-0.002
Parapithecidae + Anthropoidea	0.095	0.096	-0.001
crown Anthropoidea	0.059	0.060	0.000
Catopithecus + Catarrhini	0.045	0.044	0.000
Aegyptopithecus + Catarrhini	0.028	0.027	0.001
crown Catarrhini	-0.018	-0.017	0.000
crown Cercopithecoidea	0.046	0.046	0.000
Hominoidea	-0.072	-0.072	0.000
Hominidae	-0.106	-0.105	0.000
Homininae	0.012	0.012	0.000
Hominini	0.045	0.044	0.000
Hominina	0.147	0.148	-0.001
Homo	0.137	0.138	0.000
Dolichocebus + Platyrrhini	0.052	0.053	0.000
crown Platyrrhini	0.035	0.035	0.000
Pitheciidae	0.045	0.045	0.000
crown Pitheciinae	-0.058	-0.057	-0.001
Atelidae + Ceboidea	0.014	0.014	0.000
crown Atelidae	-0.055	-0.054	0.000
crown Ceboidea	0.005	0.006	0.000
Aotidae + Callithricidae	-0.016	-0.015	0.000
crown Callithricidae	-0.128	-0.128	0.000
crown Cebidae	0.028	0.029	-0.001

Table S17.
SURFACE model summary statistics.

	All taxa		No singular regimes		No singular regimes, no polytomies		
	Gunnell et al. 2018	Traditional	Gunnell et al. 2018	Traditional	Gunnell et al. 2018	Traditional	Extant only
Number of regimes	8	7	7	5	4	7	4
Number of shifts	16	14	14	12	9	14	5
Regimes w/convergence	6	4	4	3	2	4	1
Alpha (α)	0.041	0.027	0.057	0.031	0.031	0.061	0.008
Phylogenetic half-life	17.009	25.923	12.178	22.595	22.273	11.321	83.516
Sigma squared (σ^2)	0.0010	0.0010	0.0011	0.0010	0.0010	0.0009	0.0003
AICc	-131.409	-127.082	-146.923	-136.961	-129.896	-152.146	-88.078

Table S18.

Regime shifts and adaptive optima recovered by SURFACE analyses using two different topologies. All taxa included. Convergent lineages are indicated through cell shading. Regimes are presented graphically in Fig. S11. Problematic optima are indicated with *.

Gunnell et al. 2018 topology (based on Tree S2)

**Shift #**	**Regime optimum (ϴ)**	**Included taxa**
1	0.97	Ancestral node, anthropoids, *Djebelemur, Anchomomys,* asiadapines, scandentians
5		Tarsiers, *Omomys, Ourayia, Hemiacodon*
15		*Daubentonia*
2	0.14	*Babakotia, Palaeopropithecus*
9		*Pongo*
3	1.43	Most lemuriforms
4		Notharctids, omomyiforms (except *Omomys, Ourayia, Hemiacodon*)
6	-6.41*	*Purgatorius*
7	2.83	*Australopithecus*
8		*Donrussellia*
10	1.20	Galagids
11	-0.59*	Other plesiadapiforms (except *Carpolestes*)
12		*Ignacius*
13	0.59	Adapines, caenopithecines, lorisids, *Megaladapis*
14		Dermopterans
16		*Carpolestes*

“Traditional” topology (based on Tree S3)

**Shift #**	**Regime optimum (ϴ)**	**Included taxa**
1	0.93	Ancestral node, galagids, anthropoids, tarsiers, omomyiforms, notharctids, *Daubentonia*, *Megaladapis*, *Djebelemur*, *Anchomomys*, scandentians
2	-0.39*	*Babakotia, Palaeopropithecus*
9		*Pongo*
12		Asiadapines
3	1.39	Most lemuriforms
4	-11.44*	*Purgatorius*
5	3.40	*Donrussellia*
8		*Australopithecus*
6	-1.53*	Other plesiadapiforms (except *Carpolestes*)
7		*Ignacius*
10	0.34	Lorisids
11		Adapines, caenopithecines
13		*Carpolestes*
14		Dermopterans

Table S19.

Regime shifts and adaptive optima recovered by analyses with two different topologies. Taxa with taxon-specific regimes are removed. Convergent lineages are indicated through cell shading. Regimes are presented graphically in Fig. S12. Problematic optima are indicated with *.

Gunnell et al. 2018 topology (based on Tree S2)

Shift #	Regime optimum (Θ)	Included taxa
1	0.69	Ancestral node, scandentians, plesiadapiforms, dermopterans, *Megaladapis*, lorisids
2<br>7	0.33	*Babakotia, Palaeopropithecus*<br>*Pongo*
3<br>4<br>9	1.35	Most lemuriforms<br>Omomyiforms, notharctids<br>*Anchomomys, Djebelemur*
5<br>10<br>12<br>14	0.98	Tarsiers, *Omomys, Ourayia, Hemiacodon*<br>Anthropoids<br>*Daubentonia*<br>Adapines, caenopithecines
6	1.19	Galagids
8	45.12*	Haplorhines
11<br>13	2.30	Asiadapines<br>*Djebelemur*

"Traditional" topology (based on Tree S3)

Shift #	Regime optimum (Θ)	Included taxa
1	0.98	Ancestral node, scandentians, *Djebelemur*, *Anchomomys*, galagids, *Daubentonia*, omomyiforms, notharctids, anthropoids, tarsiers
2<br>6<br>9	-0.27*	*Babakotia, Palaeopropithecus*<br>*Pongo*<br>Asiadapines
3	1.29	Most lemuriforms
4<br>5	-1.23*	Other plesiadapiforms (except *Carpolestes*)<br>*Ignacius*
7<br>8<br>10<br>11<br>12	0.40	Lorisids<br>Adapines, caenopithecines<br>*Carpolestes*<br>Dermopterans<br>*Megaladapis*

Table S20.

Regime shifts and adaptive optima recovered by analyses with two different topologies. Taxa with taxon-specific regimes and plesiadapiforms have been removed. Convergent lineages are indicated through cell shading. Regimes are presented graphically in Fig. S13. Problematic optima are indicated with *.

Gunnell et al. 2018 topology (based on Tree S2)

Shift #	Regime optimum (ϴ)	Included taxa
1	0.98	Ancestral node, scandentians, omomyiforms, notharctids, anthropoids, tarsiers, galagids, *Daubentonia*, *Anchomomys*, *Djebelemur*
2<br>4<br>7	-0.24*	*Babakotia, Palaeopropithecus*<br>*Pongo*<br>Asiadapines
3	1.29	Most lemuriforms
6<br>5<br>8<br>9	0.41	Adapines, caenopithecines<br>Lorisids<br>Dermopterans<br>*Megaladapis*

"Traditional" topology (based on Tree S3)

Shift #	Regime optimum (ϴ)	Included taxa
1<br>13	1.61	Ancestral node<br>*Necrolemur*
2<br>4<br>5	0.27	*Babakotia, Palaeopropithecus*<br>*Pongo*<br>Asiadapines
3	1.24	Most lemuriforms
6<br>7<br>8<br>9	0.61	Adapines, caenopithecines<br>Lorisids<br>Dermopterans<br>*Megaladapis*
10	0.98	Anthropoids
11<br>14	0.78	Scandentians<br>Callitrichines
12	1.11	Primatomorpha (including tarsiers, galagids, notharctids, omomyiforms, *Daubentonia*, *Djebelemur*, *Anchomomys*)

Table S21.

Regime shifts and adaptive optima recovered by SURFACE analysis. Only extant taxa are included. Convergent lineages are indicated through cell shading. Regimes are presented graphically in Fig. S14. Problematic optima are indicated with *.

Shift #	Regime optimum (Θ)	Included taxa
1	0.74	Ancestral node, scandentians, dermopterans
2	-1.58*	*Pongo*
3	-0.08*	Lorisids
5		*Callithrix*
4	1.59	Primates

Database S1. (separate file)
Raw measurements and computed PTS indices for individual specimens.

Database S2. (separate file)
Species mean measurements and computed PTS indices.

References

1. Gunnell GF, Boyer DM, Friscia AR, Heritage S, Manthi FK, Miller ER, Sallam HM, Simmons NB, Stevens NJ, Seiffert ER (2018) Fossil lemurs from Egypt and Kenya reveal an African origin for Madagascar's aye-aye. *Nature Commun* 9:3193.
2. Herrera JP, Dávalos LM (2016) Phylogeny and divergence times of lemurs inferred with recent and ancient fossils in the tree. *Syst Biol* 65:772–791.
3. Kay RF (2015) Biogeography in deep time – What do phylogenetics, geology, and paleoclimate tell us about early platyrrhine evolution? *Mol Phylogenet Evol* 82:358–374.
4. Gunnell GF (2002) Notharctine primates (Adapiformes) from the early to middle Eocene (Wasatchian–Bridgerian) of Wyoming: transitional species and the origins of *Notharctus* and *Smilodectes*. *J Hum Evol* 43:353–380.
5. Ronquist F, Teslenko M, van der Mark P, Ayres DL, Darling A, Höhna S, Larget B, Liu L, Suchard MA, Huelsenbeck JP (2012) MrBayes 3.2: efficient Bayesian phylogenetic inference and model choice across a large model space. *Syst Biol* 61:539-542.
6. Springer MS, Meredith RW, Gatesy J, Emerling CA, Park J, Rabosky DL, Stadler T, Steiner C, Ryder OA, Janečka JE, Fisher CA (2012) Macroevolutionary dynamics and historical biogeography of primate diversification inferred from a species supermatrix. *PLoS One* **7**, e49521.
7. Yapuncich GS, Seiffert ER, Boyer DM (2017) Quantification of the position and depth of the flexor hallucis longus groove in euarchontans, with implications for the evolution of primate positional behavior. *Am J Phys Anthropol* 163:367-406.
8. Boyer DM, Seiffert ER (2013) Patterns of astragalar fibular facet orientation in extant and fossil primates and their evolutionary implications. *Am J Phys Anthropol* 151:420-447.
9. Boyer DM, Yapuncich GS, Butler JE, Dunn RH, Seiffert ER (2015) Evolution of postural diversity in primates as reflected by the size and shape of the medial tibial facet of the talus. *Am J Phys Anthropol* 157:134-177.
10. Tornow MA (2008) Systematic analysis of the Eocene primate family Omomyidae using gnathic and postcranial data. *Bull Peabody Mus Nat Hist* 49:43-129.
11. Beard KC, Qi T, Dawson MR, Wang B, Li C (1994) A diverse new primate fauna from middle Eocene fissure-fillings in southeastern China. *Nature* 368:604-609.
12. Stevens NJ, Seiffert ER, O'Connor PM, Roberts EM, Schmitz MD, Krause C, Gorscak E, Ngasala S, Hieronymus TL, Temu J (2017) Palaeontological evidence for an Oligocene divergence between Old World monkeys and apes. *Nature* 497:611-614.
13. Parr WCH, Soligo C, Smaers J, Chatterjee HJ, Ruto A, Cornish L, Wroe S (2014) Three-dimensional shape variation of talar surface morphology in hominoid primates. *J Anat* 225:42-59.
14. Yapuncich GS, Boyer DM (2015) Allometric shape change in the talar articular surfaces of euarchontans. *Am J Phys Anthropol* 156:328.
15. Cartmill M, Milton K (1977) The lorisiform wrist joint and the evolution of "brachiating" adaptations in the Hominoidea. *Am J Phys Anthropol* 47:249-272.
16. Fleagle JG (1985) Size and adaptation in primates. *Size and Scaling in Primate Biology*, ed Fleagle JG (Springer, Boston), pp 1-19.
17. Hammer Ø, Harper DAT, Ryan PD (2001) PAST: Paleontological statistics software package for education and data analysis. *Paleontol Electron* 4:1–9.

18. Decker RL, Szalay FS (1974) Origin and function of the pes in the Eocene Adapidae (Lemuriformes, Primates). *Primate Locomotion*, ed Jenkins FA (Academic Press, New York), pp 261-291.
19. Gebo DL (1986) Anthropoid origins–the foot evidence. *J Hum Evol* 15:421-430.
20. Gebo DL (1988) Foot morphology and locomotor adaptation in Eocene primates. *Folia primatol* 50:3-41.
21. Gebo DL, Dagosto M, Beard KC, Qi T, Wang J (2000) The oldest known anthropoid postcranial fossils and the early evolution of higher primates. *Nature* 404:276-278.
22. Gebo DL, Dagosto M, Beard KC, Qi T (2001) Middle Eocene primate tarsals from China: Implications for haplorhine evolution. *Am J Phys Anthropol* 116:83-107.
23. Marivaux L, Chaimanee Y, Ducrocq S, Marandat B, Sudre J, Soe AN, Tun ST, Htoon W, Jaeger JJ (2003) The anthropoid status of a primate from the late middle Eocene Pondaung Formation (Central Myanmar): Tarsal evidence. *Proc Nat Acad Sci USA* 100:13173-13178.
24. Dagosto M, Marivaux L, Gebo DL, Beard KC, Chaimanee Y, Jaeger JJ, Marandat B, Soe AN, Kyaw AA (2010) The phylogenetic affinities of the Pondaung tali. *Am J Phys Anthropol* 143:223-234.
25. Dagosto M (1988) Implications of postcranial evidence for the origin of euprimates. *J Hum Evol* 17:35-56.
26. Sargis EJ (2002) The postcranial morphology of *Ptilocercus lowii* (Scandentia, Tupaiidae): An analysis of primatomorphan and volitantian characters. *J Mamm Evol* 9:137–160.
27. Sargis EJ (2007) The postcranial morphology of *Ptilocercus lowii* (Scandentia, Tupaiidae) and its implications for primate supraordinal relationships. Primate Origins: Adaptations and Evolution, eds Ravosa M, Dagosto M (Springer, New York), pp 51–82.
28. Bloch JI, Silcox MT, Boyer DM, Sargis EJ (2007) New Paleocene skeletons and the relationship of plesiadapiforms to crown-clade primates. *Proc Nat Acad Sci USA* 104:1159-1164.
29. Boyer DM, Bloch JI (2008) Evaluating the mitten-gliding hypothesis for Paromomyidae and Micromomyidae (Mammalia,"Plesiadapiformes") using comparative functional morphology of new Paleogene skeletons. *Mammalian Evolutionary Morphology*, eds Sargis EJ, Dagosto M (Springer, Dordrecht), pp 233-284.
30. Boyer DM, Toussaint S, Godinot M (2017) Postcrania of the most primitive euprimate and implications for primate origins. *J Hum Evol* 111:202-215.
31. Dagosto M (1983) Postcranium of *Adapis parisiensis* and *Leptadapis magnus* (Adapiformes, Primates). *Folia primatol* 41:49-101.
32. Seiffert ER, Costeur L, Boyer DM (2015) Primate tarsal bones from Egerkingen, Switzerland, attributable to the middle Eocene adapiform *Caenopithecus lemuroides*. *PeerJ* 3:e1036.
33. Boyer DM, Seiffert ER, Simons EL (2010) Astragalar morphology of *Afradapis*, a large adapiform primate from the earliest late Eocene of Egypt. *Am J Phys Anthropol* 143:383-402.
34. Rose KD, Rana RS, Sahni A, Kumar K, Missiaen P, Singh L, Smith T (2009) Early Eocene primates from Gujarat, India. *J Hum Evol* 56:366–404.

35. Dunn RH, Rose KD, Rana RS, Kumar K, Sahni A, Smith T (2016) New euprimate postcrania from the early Eocene of Gujarat, India, and the strepsirrhine-haplorhine divergence. *J Hum Evol* 99:25–51.
36. Boyer DM, Seiffert ER, Gladman JT, Bloch JI (2013) Evolution and allometry of calcaneal elongation in living and extinct primates. *PLoS One* 8:e67792.
37. Schmitt D (1994) Forelimb mechanics as a function of substrate type during quadrupedalism in two anthropoid primates. *J Hum Evol* 26:441-457.
38. Patel BA, Larson SG, Stern Jr. JT (2015) Electromyography of crural and pedal muscles in tufted capuchin monkeys (*Sapajus apella*): implications for hallucal grasping behavior and first metatarsal morphology in euprimates. *Am J Phys Anthropol* 156:553-564.
39. Gray EG, Basmajian JV (1968) Electromyography and cinematography of leg and foot ("normal" and flat) during walking. *Anat Rec* 161:1-16
40. Stern Jr. JT, Susman RL (1983) The locomotor anatomy of *Australopithecus afarensis*. *Am J Phys Anthropol* 60:279-317.
41. Lovejoy CO, Latimer B, Suwa G, Asfaw B, White TD (2009) Combining prehension and propulsion: the foot of *Ardipithecus ramidus*. *Science*, 326, 72.
42. Langdon JH (1990) Variations in cruropedal musculature. *Int J Primatol* 11:575-606.
43. Edama M, Kubo M, Onishi H, Takabayashi T, Yokoyama E, Inai T, Watanabe H, Nashimoto S, Kageyama I (2016) Anatomical study of toe flexion by flexor hallucis longus. *Annals Anat* 204:80-85.
44. Beard KC (1990) Gliding behaviour and palaeoecology of the alleged primate family Paromomyidae (Mammalia, Dermoptera). *Nature* 345:340.
45. Beard KC (1993) Phylogenetic systematics of the Primatomorpha, with special reference to Dermoptera. *Mammal Phylogeny: Placentals*, eds Szalay FS, Novacek MJ, McKenna MC, (Springer, New York), pp 129–150.
46. Beard KC (1993) Origin and evolution of gliding in early Cenozic Dermoptera (Mammalia, Primatomorpha). *Primates and their Relatives in Phylogenetic Perspective*, ed MacPhee R, (Plenum Press, New York), pp 63–90.
47. Bloch JI, Boyer DM (2007) New skeletons of Paleocene-Eocene Plesiadapiformes: a diversity of arboreal positional behaviors in early primates. *Primate Origins: Adaptations and Evolution*, eds Ravosa MJ, Dagosto M (Springer, Boston), pp 535-581.
48. Krause DW (1991) Were paromomyids gliders? Maybe, maybe not. *J Hum Evol* 21:177-188.
49. Runestad JA, Ruff CB (1995) Structural adaptations for gliding in mammals with implications for locomotor behavior in paromomyids. *Am J Phys Anthropol* 98:101-119.
50. Szalay FS, Lucas SG (1996) The postcranial morphology of Paleocene *Chriacus* and *Mixodectes* and the phylogenetic relationships of archontan mammals. *Bull New Mexico Mus Nat Hist Sci* 7:1-47.
51. Hamrick MW, Rosenman BA, Brush JA (1999) Phalangeal morphology of the Paromomyidae (?Primates, Plesiadapiformes): the evidence for gliding behavior reconsidered. *Am J Phys Anthropol* 109:397-413.
52. Kirk EC, Lemelin P, Hamrick MW, Boyer DM, Bloch JI (2008) Intrinsic hand proportions of euarchontans and other mammals: implications for the locomotor behavior of plesiadapiforms. *J Hum Evol* 55:278-299.
53. Dagosto M (1986) The joints of the tarsus in the strepsirhine primates: functional, adaptive, and evolutionary implications (dissertation) City University of New York.

54. Gebo DL (1988) Foot morphology and locomotor adaptation in Eocene primates. *Folia primatol* 50:3–41.
55. Jungers WL, Godfrey LR, Simons EL, Chatrath PS (1997) Phalangeal curvature and positional behavior in extinct sloth lemurs (Primates, Palaeopropithecidae). *Proc Nat Acad Sci USA* 94:11998-12001.
56. Shapiro LJ, Seiffert CV, Godfrey LR, Jungers WL, Simons EL, Randria GF (2005) Morphometric analysis of lumbar vertebrae in extinct Malagasy strepsirrhines. *Am J Phys Anthropol* 128:823-839.
57. Marchi D, Ruff CB, Capobianco A, Rafferty KL, Habib MB, Patel BA (2016) The locomotion of *Babakotia radofilai* inferred from epiphyseal and diaphyseal morphology of the humerus and femur. *J Morphol* 277:1199-1218.
